# Light-induced assembly and repeatable actuation in Ca^2+^-driven chemomechanical protein networks

**DOI:** 10.1038/s41467-026-69651-2

**Published:** 2026-02-21

**Authors:** Xiangting Lei, Carlos Floyd, Laura Casas-Ferrer, Tuhin Chakrabortty, Nithesh Chandrasekharan, Aaron R. Dinner, Scott Coyle, Jerry Honts, Saad Bhamla

**Affiliations:** 1 School of Chemical and Biomolecular Engineering, Georgia Institute of Technology, Atlanta, GA USA; 2 Department of Chemistry and James Franck Institute, University of Chicago, Chicago, IL USA; 3 Department of Biochemistry, University of Wisconsin Madison, Madison, WI USA; 4 Department of Biology, Drake University, Des Moines, IA USA; 5 BioFrontiers Institute and Department of Chemical and Biological Engineering, University of Colorado Boulder, Boulder, CO USA

**Keywords:** Biomaterials - proteins, Biophysics

## Abstract

Programming rapid, repeatable motions in soft materials has remained a challenge in active matter and biomimetic design. Here, we present a light-controlled chemomechanical network based on *Tetrahymena thermophila* calcium-binding protein 2 (Tcb2), a Ca^2+^-sensitive contractile protein. These networks—driven by Ca^2+^-triggered structural rearrangements—exhibit dynamic self-assembly, spatiotemporal growth, and contraction rates comparable to actomyosin systems. By coupling light-sensitive chelators for optically triggered Ca^2+^ release, we achieve precise growth and repeatable mechanical contractility of Tcb2 networks, revealing emergent phenomena such as boundary-localized active regions and density gradient-driven reversals in motion. A coupled reaction-diffusion and elastic model explains these dynamics, highlighting the interplay between chemical network assembly and mechanical response. We further demonstrate active transport of particles via network-mediated forces in vitro and implement reinforcement learning to program seconds-scale spatiotemporal actuation in silico. These results establish a platform for designing responsive active materials with rapid chemomechanical dynamics and tunable optical control, with applications in synthetic cells, sub-cellular force generation, and programmable biomaterials.

## Abstract (web summary)

Programming ultrafast, reversible motions at the micron scale in subcellular soft materials and active matter requires precise spatiotemporal control over molecular processes. Here, the authors investigate elastic cortical protein networks that assemble rapidly and demonstrate repeatable force generation that actuates using light-sensitive Ca2+ release.

## Introduction

In recent years, there has been significant research focus on chemomechanical materials in which chemical or optical cues drive mechanical and structural changes^1^. Both purely synthetic and bio-inspired materials have been developed with design goals such as reversible self-assembly, on-demand force generation, and spatial programmability. For example, synthetic approaches have coupled pattern-forming chemical reactions with hydrogels to induce periodic swelling and force generation^2–6^; however, such motions are often slow and difficult to direct, as they rely on intrinsic chemical oscillations. Light-driven strategies, including the covalent assembly of polymer networks^7,8^, offer greater external control but tend to form long-lived structures unsuitable for dynamic force production. Reversible self-assembly has also been demonstrated in polymeric and liquid crystalline materials^9–12^, though these systems typically respond to uniform chemical triggers and lack fine spatial control.

As an alternative to purely synthetic systems, bio-derived chemomechanical materials offer evolved platforms for non-equilibrium force generation. These include cellular and bacterial suspensions^13,14^, cilia carpets^15,16^, and, notably, assemblies of cytoskeletal filaments and motor proteins like actomyosin or microtubule-kinesin mixtures^17–27^. Recent bioengineering of light-sensitive molecular motors enables dynamical control over force generation using spatiotemporal light patterning in cytoskeletal materials^21–23,28–35^. However, light-controllable cytoskeletally-derived materials face limitations such as flows constrained to filament orientations and reliance on complex molecular bioengineering. To our knowledge no synthetic or bio-derived material has yet demonstrated the simultaneous combination of self-assembly triggered by spatial light patterning, on-demand repeatable and reorientable force generation, fast (seconds) assembly rates, a bioorthogonal energy source, and minimal fabrication complexity. A material with these properties would be an excellent candidate for application in synthetic cell biology, in which light-activated rapid force-generation could be used to guide intracellular dynamics.

While most biomechanical processes are powered by a direct coupling between hydrolysis of adenosine triphosphate (ATP) and molecular motion, nature has also developed alternative paradigms for generating biological motion^36–38^. In various heterotrich ciliates, contractile motion arises from specialized Ca^2+^-binding protein assemblies which in different organisms have been termed myonemes^36,39^ and spasmonemes^38,40^. Our comparative genomics analyses reveal sequence similarities among these various contractile proteins (SI I A). Unlike traditional cytoskeletal systems, these ciliate contractile systems generate motion through direct, Ca^2+^-induced structural rearrangements of the polymers^41^, rather than through relative sliding of filament pairs. These systems generate repeatable forces for ultrafast in vivo contractions—the fastest known motions in biology^36^—and are roughly ten or more times faster than actomyosin-based networks (see Discussion). As a result, these Ca^2+^-driven protein systems hold promise for deriving new force-generating biomaterials with faster dynamics than traditional cytoskeletal components.

To begin theoretically understanding their dynamics, in Ref. ^42^ we modeled ciliates’ myoneme-based contraction in vivo, which is triggered by a propagating wave of Ca^2+^ in the cytosol. However, despite this preliminary modeling and related studies^43–48^, our quantitative understanding of these naturally evolved protein networks remains limited compared to cytoskeletal systems. Furthermore, experimental methods akin to those used for reconstituting and controlling cytoskeletal materials in vitro using spatiotemporal light patterning are currently unavailable for Ca^2+^-triggered proteins. Finally, while theoretical and computational advances have facilitated the design of control strategies for light-triggered cytoskeletal systems^49–56^, such methods are currently lacking from our toolkit to study Ca^2+^-triggered protein dynamics.

Here, we purify and reconstitute polymeric networks of *Tetrahymena* calcium-binding protein 2 (Tcb2), a Ca^2+^-sensitive contractile protein from the ciliate *Tetrahymena thermophila*^57,58^. Although its physiological function is not fully known, it is thought to play a role in Ca^2+^-dependent processes such as ciliary movement, pronuclear exchange, and signaling in the cortex.^57,59,60^. This protein is homologous to the Ca^2+^-binding proteins that underlie ultrafast contraction in other ciliates, and we focus on it here because there are available molecular tools for its expression and it does not require co-synthesis with a scaffold protein (SI I A).

We develop an experimental methodology to reconstitute a contractile Tcb2 protein network in vitro and spatiotemporally control it using light-triggered Ca^2+^ release by diffusing chelators. We show that this system exhibits rich dynamical behaviors, including a Ca^2+^-driven active region (CAR) near the boundary and a reversal of contractility direction, from radially inward to outward. We explain these dynamics by introducing a continuum model of an overdamped elastic solid with inhomogeneous density and an elastic rest length dependent on the local Ca^2+^ concentration, which we couple with a reaction-diffusion-based description of the chemical components. Additionally, we demonstrate preliminary control over the transport of external particles by the Tcb2 networks in vitro, as well as fine-grained control over network contraction in silico using reinforcement learning. This system represents a new biologically derived soft material for producing repeatable and rapidly responsive forces at subcellular scales. We conclude by discussing its potential applications in synthetic biology.

## Results

### Spatiotemporal light patterning controls Tcb2 protein network dynamics

We first aim to develop an experimental platform for studying Ca^2+^-driven contractile proteins in vitro. The ultrafast contractile motion of ciliates involves assemblies such as myonemes, and it is driven by rapid waves of Ca^2+^ released from internal stores such as the endoplasmic reticulum (ER)^36,42,48^. After the organism contracts, ATP is hydrolyzed to pump Ca^2+^ back into the ER to reset the process. Several ciliate species in the “atlas” of ultrafast cells exhibit high-speed contraction^47^, but they are not model biological systems and, hence, we currently lack sufficient experimental protocols to study them using protein reconstitution. However, homologs of centrin (one of the main components of myonemes) which also interact with Ca^2+^, though not observed to produce whole-cell ultrafast cellular contractions, have been identified in other model ciliates (SI I A). Here, we focus on the homologous protein Tcb2 from *Tetrahymena thermophila*, which we show exhibits a marked structural response upon binding to Ca^2+^^41^, making it a valuable model for studying Ca^2+^-driven contractile proteins. Using available molecular tools we express this protein in ample quantities, allowing for straightforward reconstitution in vitro.

Tcb2 is a 25 kDa Ca^2+^-binding protein found in the cortical layer of *Tetrahymena*^59^. Tcb2 has four EF-hand domains with putative Ca^2+^-binding sites^41,57,61^. In the absence of a solved crystal structure, we use AlphaFold3^62^ to predict the structure of Tcb2 monomers and their conformational changes after binding Ca^2+^. The predicted conformational changes are consistent with previous NMR data^41^ (SI I A), and these conformational changes underscore the potential of using this protein in a chemomechanical material with contractile dynamics.

**Fig. 1:**
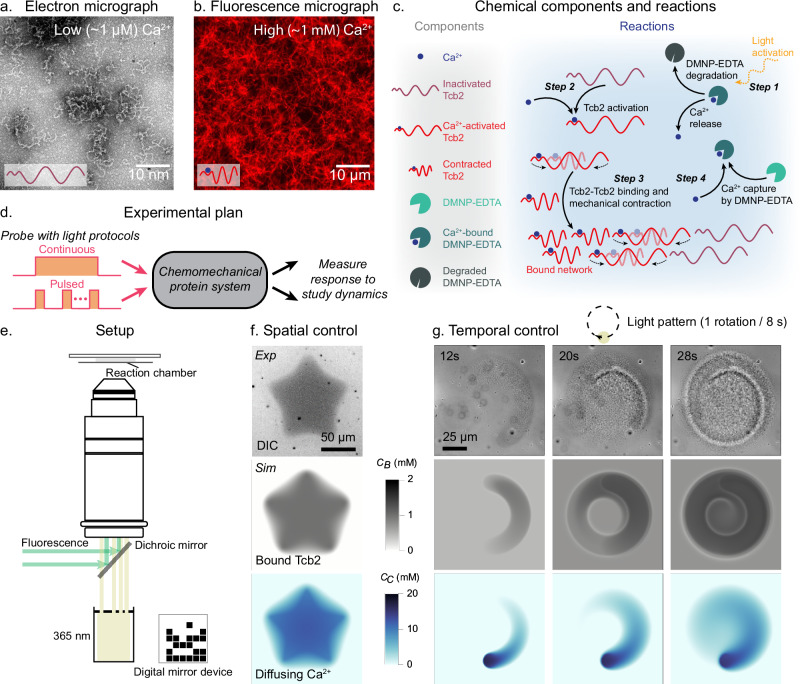
Experimental control of Tcb2 network assembly. **a** Tcb2 filaments observed at ~ 1 μM Ca^2+^ concentrations using electron microscopy, showing typical filament lengths of approximately 250–500 nm. **b** Tcb2/Tcb2-mCherry filaments in the presence of ~ 1 mM Ca^2+^ observed via fluorescence microscopy. **c** Chemical components and reactions involving Tcb2 and Ca^2+^, including light activation, DMNP-EDTA binding/unbinding, and Tcb2 binding/unbinding. **d** Schematic illustration of the experimental plan in this paper. **e** Schematic illustration of the experimental setup. **f** Tcb2 network formation, imaged using DIC, for a star-shaped DMD pattern. Simulated bound Tcb2 (*C*_*B*_) and diffusing Ca^2+^ (*C*_*C*_) concentrations are shown below. **g** Dynamic Tcb2 network formation using a cyclic light pattern, with accompanying simulations of bound Tcb2 and diffusing Ca^2+^ concentrations. The light moves in a circle at 0.125 Hz, shown at different cycles.

We clone DNA encoding a synthetic Tcb2 gene that was codon-optimized for bacterial expression into an expression plasmid. We then express and purify the protein from *E. coli* as in Ref. ^63^ (Methods and SI I C). We observe that purified Tcb2 alone, without additional scaffolding proteins, readily forms fibrous protein networks in response to Ca^2+^ ions and subsequently contracts along the network boundary (SI Video Part 1, Section I). Electron microscopy reveals fine, irregular filaments around 250-500 nm at low Ca^2+^ concentrations (Fig. 1a). At higher Ca^2+^ concentrations we observe denser and large-scale networks of filamentous protein as shown by micrographs with fluorescent mCherry-tagged Tcb2 at a larger spatial scale (Fig. 1b). This propensity to self-assemble into a cortex-like^21^ network distinguishes Tcb2 from other Ca^2+^-binding proteins like calmodulin^64^.

We employ an experimental methodology that releases Ca^2+^ in a spatiotemporally controlled manner using photolyzable Ca^2+^ chelators. The DMNP-EDTA Ca^2+^ chelator has a photolabile bond, which, when severed upon illumination by 365 nm light, releases Ca^2+^ in milliseconds^65^ (SI I D). This complex has been used extensively in optogenetics^66–68^, and we adapt it here to achieve precise optical control of Ca^2+^ release in Tcb2 solutions. We note that in contrast to the situation in vivo, in which energy is provided to the system via ATP-driven pumping of Ca^2+^ ions and their subsequent rapid release from intracellular stores, here energy is provided by photons which cleave Ca^2+^ chelators. These are different implementations of the same underlying logic: Ca^2+^ storage through intracellular stores or DMNP-EDTA binding, and release through calcium channel opening or photo-cleavage of chelating groups. In Fig. 1c we schematically show the chemical components and reactions of the system, including chelators, Ca^2+^ ions, Tcb2 proteins, and externally applied light. Our goal in this work is to use canonical light protocols, such as step functions and periodic pulses, to probe the unknown dynamical properties of this material (Fig. 1d)^69^.

To spatiotemporally control DMNP-EDTA photolysis we use a custom optical setup, integrating a digital micromirror device (DMD) pattern illuminator into a microscope (SI I D). This setup uses dual optical pathways via a multi-port illuminator, enabling both fluorescence and 365 nm illumination (Fig. 1e). This system provides sub-micrometer spatial resolution and millisecond temporal precision^70^. To illustrate dynamical control using this system, we project specific spatial and temporal patterns of illumination onto the Tcb2 solution. We illuminate a sharp star shape using the DMD and, within one second, observe a corresponding pattern in the differential interference contrast (DIC) images of the Tcb2 solution, indicating the formation of a Ca^2+^-bound Tcb2 network at the corresponding location (Fig. 1f). These patterns can also be programmed into time series, enabling more complex manipulation of the network. We demonstrate this using a circular illumination pattern traveling in a circular trajectory at 0.125 Hz (Fig. 1g and SI Video Part 1 Section II). We note that the timescale of the observed assembly of Tcb2 networks is seconds. This is faster than those observed in actomyosin or microtubule-kinesin systems, which typically require minutes to hours to assemble and achieve similar dynamics^30,71^.

We augment this experimental setup with a continuum reaction-diffusion model of the chemical system illustrated in Fig. 1c (Methods and the SI I E). Under reasonable estimates for the unknown model parameters we observe qualitative agreement between the predictions of the model and key experimentally observed features of Tcb2 network dynamics (Figs. 1f,g). We elaborate on details of this model as they become relevant below.

### A Ca^2+^-driven active region results from the contraction dynamics of diffusion-limited growth

**Fig. 2:**
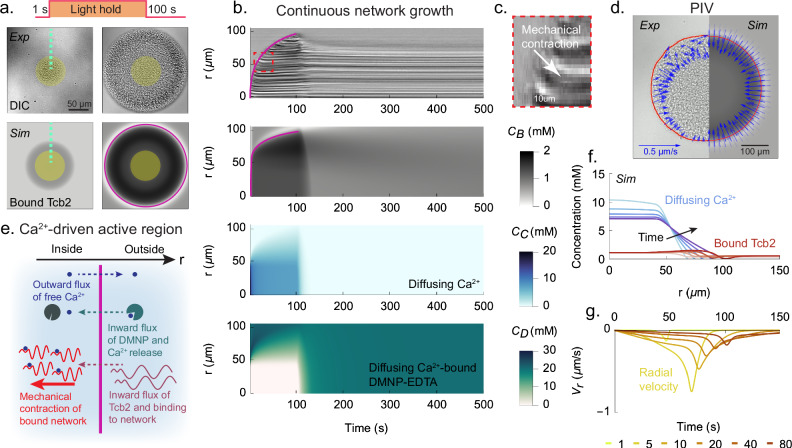
Continuous growth of the network produces an active region. **a** *Top row*: DIC images of Tcb2 network with circular illumination at 1 s and 100 s in a continuous light protocol, using an illumination diameter of 75 μm. Experiments are repeated three times. *Bottom row*: Corresponding Tcb2 simulation images. **b** *First row:* Kymograph constructed from DIC images for the continuous light experiment in (**a**), taken along the green dashed line. *Second row*: Simulated kymograph, showing bound Tcb2 concentration as a function of radial position. *Third row*: Same as second row, except showing diffusing Ca^2+^ concentration. *Fourth row*: Same as second row, except showing Ca^2+^-DMNP-EDTA concentration. **c** Magnified view of contracting region (red dashed box in the top row of **b**). **d** Experimental PIV field and corresponding simulation image at 100 s. Minor angular asymmetries occasionally visible in experimental PIV arise from stochastic experimental non-uniformities and are not reproducible across replicates. **e** Schematic illustration of the CAR. **f** Concentration of Ca^2+^ (blue) and bound Tcb2 (red) over time. The colors range from light to dark and match the times in the next panel. **g** Radial velocity profiles *V*_*r*_(*r*, *t*) of the network over time.

Having established the experimental and computational setup, we next seek to characterize the chemomechanical dynamics of the Tcb2 solution systematically using standard light activation protocols such as step functions and pulses (Fig. 1d). We first characterize the growth dynamics of the Tcb2 protein network by projecting a sustained circular light pattern on the solution (SI Video Part 1 Section II). Unless otherwise indicated, all results in this paper use a 75 μm diameter for the illuminated circle, but in SI II A we show results using different illumination diameters. During 100 s of illumination we observe that first, upon illumination, Tcb2 quickly forms a connected network within the illuminated region (Figs. 2a,b), where the Ca^2+^ concentration increases suddenly due to chelator photolysis. Over time the network grows radially outward (Fig. 2a at 100 s and Fig. 2b). In SI Fig. S11 we show that the detected radius of the network scales approximately as the square root of time, which indicates diffusion-limited growth dynamics. The growth rate also increases with the diameter of the illumination region. We additionally observe outward diffusion of Ca^2+^ using rhodamine-2, a Ca^2+^ sensitive dye (SI Fig. S20). In ref. ^72^ we further develop calibrated rhodamine assays to quantify growth dynamics of Tcb2 networks.

We observe that in the growing network the region near the boundary is mechanically active. The newly formed network at the boundary contracts radially inward, as shown by a kymograph (Figs. 2b,c) and particle image velocimetry (PIV, Fig. 2d). We refer to this boundary as a Ca^2+^-driven active region (CAR, Fig. 2e) and summarize the processes within the CAR as follows. Chemically, excess Ca^2+^ ions released from photolyzed DMNP-EDTA that have not yet bound to Tcb2 diffuse outward, while unphotolyzed Ca^2+^-DMNP-EDTA complexes diffuse inward, both following their respective concentration gradients. As these complexes reach the illuminated zone they photolyze and release additional Ca^2+^, sustaining a loop that fuels network growth by continuously supplying Ca^2+^ from the inside. Tcb2 proteins diffuse inward at the CAR, where they bind to both other Tcb2 proteins and available Ca^2+^, joining the growing network. Based on molecular size, we estimate the diffusion constant of Tcb2 monomers to be 10 μm^2^/*s*, and we show in SI Fig. S27 that accounting for Tcb2 diffusion is necessary to explain the network’s growth dynamics. Upon Ca^2+^ binding, Tcb2’s rest length shortens, prompting rapid, inward contraction. Since the network’s interior has reached mechanical equilibrium, contraction occurs mainly at the periphery.

To describe the contractile dynamics of this system computationally, we augment the chemical variables in the continuum model with a two-dimensional displacement field that obeys the dynamics of an overdamped linearly elastic gel^73^. We couple this mechanical representation to the chemical fields in two ways. First, because in a typical Ashby plot^74^ the elastic moduli of materials increase with their density, we make the elastic moduli of the gel proportional to the local concentration of bound Tcb2. We explore non-linear dependencies between density and stiffness in SI Fig. S7, showing a qualitative insensitivity to this modeling choice. Second, following our previous model^42^ for in vivo ultrafast myoneme contraction in ciliates, we decrease the local rest length of the material linearly with the fraction of bound Tcb2 (SI I E). This decrease of local rest length, which can be viewed as the equilibrium end-to-end length of the Tcb2 monomers, reflects the conformational change in Tcb2 monomers suggested by NMR data and AlphaFold3 predictions mentioned above. We assume that this conformational change underlies the mechanical contractility of the macroscopic network although at a coarse-grained modeling level the molecular details of contraction are not critical^41^ (SI I A).

Using the model, which allows us to spatially resolve all of the chemical components (SI Fig. S18), we observe that the boundary of the Tcb2 network closely follows the leading edge of the diffusing Ca^2+^ profile as shown in Fig. 2f. This supports the picture that the outward growth of the Tcb2 network is driven by the diffusion of Ca^2+^ out from the illuminated region and of Tcb2 into the bound network region. The model reproduces the CAR, such that the contracting radial velocity peak is located near the periphery of the Tcb2 network, moving radially outward as the network grows (Fig. 2g). We further find that, to prevent the growth from stalling at finite radius due to the balance of reaction-diffusion fluxes, it is necessary to account for the degradation of DMNP-EDTA chelators in the model (SI Fig. S22).

After the light is turned off in the experiments, Ca^2+^ rapidly unbinds from the network and is resequestered by the available DMNP-EDTA chelator. Upon unbinding of Ca^2+^ and re-elongation of the Tcb2 rest lengths (via conformational change of Tcb2 monomers back to the Ca^2+^-free state with a longer equilibrium end-to-end distance), the network quickly relaxes from its contracted state and expands outward (SI Video Part 1 Section III). It then slowly dissolves via the unbinding of Tcb2 from other Tcb2 proteins. The slow timescale of chemical growth and dissolution is distinct from the fast timescale of mechanical contraction and expansion (SI Fig. S21). A similar interplay between rapid mechanical responses and slower chemical reorganization has been observed in simulations of actomyosin networks under tensile force^75^. We next aim to leverage these two contrasting timescales to overcome the size limitation which arises from the actively contracting region advancing outward in a single sweep and leaving most of the internal region mechanically equilibrated in its path.

### Temporally pulsed illumination yields a larger and faster Ca^2+^-driven active region

**Fig. 3:**
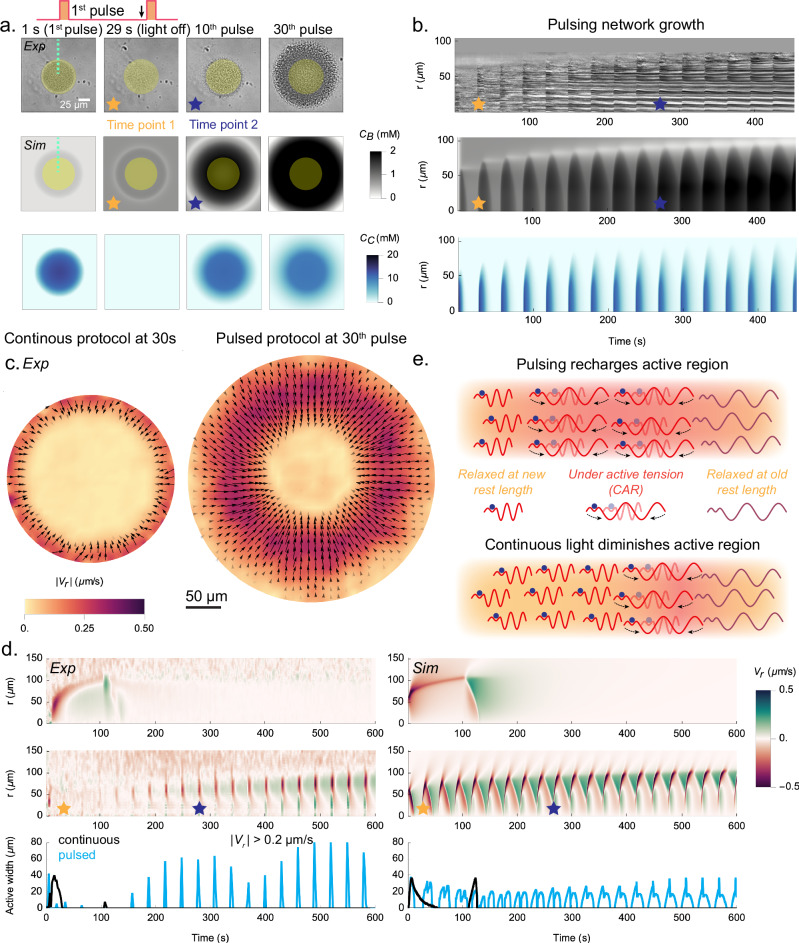
Pulsed light allows the CAR to recharge. **a** DIC images of a pulsed illumination experiment at 0 s, 29 s (right before the 2nd pulse), the 10th pulse, and the 30th pulse. Corresponding simulation images of the bound Tcb2 and diffusing Ca^2+^ profiles are shown below. The blue and yellow stars indicate the same pulses (time points) throughout the figure. The dashed green line indicates the region used to generate the kymograph shown in (**b**). **b** Kymographs of the DIC images shown above corresponding simulation results. **c** PIV of continuous light at 30 s (left) and the 30th cycle of the pulsed protocol, with arrows indicating the direction of network motion. The background color represents velocity magnitude and the arrows represent the local velocity vector. **d** *Top row*: Experimental and simulated velocity kymographs for the continuous protocol. *Middle row*: Experimental and simulated velocity kymographs for the pulsed protocol. *Bottom row*: Experimental and simulated plots of the CAR width, defined as the radial extent of the system for which ∣*V*_*r*_∣ > 0.2 μm/s, as a function of time for the continuous and pulsed protocols. **e** Schematic illustration of the Tcb2 recharging mechanism. See Fig. 1c for a legend of the molecular components. Experiments are repeated three times.

In an effort to “recharge” the system, we next investigate the use of a pulsed, or ratcheting, illumination protocol: a 1 s flash of illumination followed by 29 s of darkness, repeated in cycles. During the initial few cycles, the network formed in the 1 s flash mostly dissolves back into solution during the light-off phase (Fig. 3a at 1 s and 29 s). After these initial cycles, a persistent network begins to form and then grow in size with each subsequent pulse (Fig. 3a at 10th and 30th pulse and Fig. 3b). In SI II A, II I, and II J, we study the dependence of growth dynamics on illumination size, pulse frequency, and DMNP-EDTA concentration. In SI II B and SI Video Part 2 Section VI, we demonstrate sustained contractile responses over  ~ 150 cycles without significant performance degradation. The system maintains consistent contraction speeds of  ~ 0.4 *μ*m/s across illumination diameters ranging from 50 to 100 μm, highlighting its potential for applications requiring repeated mechanical actuation.

In Fig. 3c we show PIV measurements comparing the continuous illumination protocol, evaluated at 30 s, with the pulsed protocol, evaluated at the 30th cycle (i.e., the same total time of illumination). In the continuous protocol the CAR is relatively slender and has low average radial velocity (width:  ~ 13 μm, $\overline{{V}_{r}}$: 0.21 μm/s), while in the pulsed protocol the CAR is significantly larger and faster (width:  ~ 62 μm, $\overline{{V}_{r}}$: 0.52 μm/s); see SI Fig. S23. Importantly, pulsing also causes the CAR to remain large over many repeated cycles (middle row Fig. 3d). To quantify this we measure the width of the radial domain for which the norm of the radial velocity exceeds 0.2 μm/s (chosen as an arbitrary cutoff value), which we plot as a function of time at the bottom of Fig. 3d. These measurements demonstrate that, in contrast to the continuous protocol, pulsed light effectively recharges the system and allows for repeated generation of large contractile forces that act over nearly the whole network.

Simulations qualitatively reproduce these findings, allowing closer investigation of the recharging mechanism (Fig. 3d,e). Each Tcb2 filament has a rest length that shrinks upon binding Ca^2+^, supported by earlier NMR data^41^ and consistent with structural changes suggested by AlphaFold3 (SI I A). Each filament can thus be viewed as being in one of two states with different rest lengths, depending on whether Ca^2+^ is bound to the filament. Upon Ca^2+^-binding, but before the filament contracts toward the shorter rest length, there is a build up of mechanical potential energy in the system (SI Fig. S19). The Ca^2+^-bound filaments then contract in length to reduce this energy. In the pulsed light protocol, the dark phase allows Ca^2+^ to quickly unbind from contracted filaments, restoring their longer rest lengths. The filaments then mechanically relax to these lengths, but due to the slower unbinding of Tcb2 molecules from each other the network does not fully dissolve during the dark phase. In this way, the bound Tcb2 state is “ratcheted” while the mechanical state is “reset.” The extended Tcb2 filaments are then ready to contract again during the subsequent pulse of light. By contrast, in the continuous protocol the network grows steadily, binding to new Ca^2+^ at the periphery while the interior remains static and stress-free once mechanically equilibrated under the shortened rest lengths. We emphasize that these networks demonstrate rapid and repeatable mechanical force generation through rapid Ca^2+^ binding and unbinding kinetics. However, the Tcb2 network assembly itself is not rapidly reversible due to the slow dissolution process governing Tcb2-Tcb2 unbinding.

The recharging mechanism in the pulsed protocol enables repeatable seconds-scale contractile dynamics, offering potential for Tcb2 networks to generate controlled micron-scale mechanical forces using light-based protocols.

### Spatially heterogeneous patterns of simultaneous contraction and expansion

**Fig. 4:**
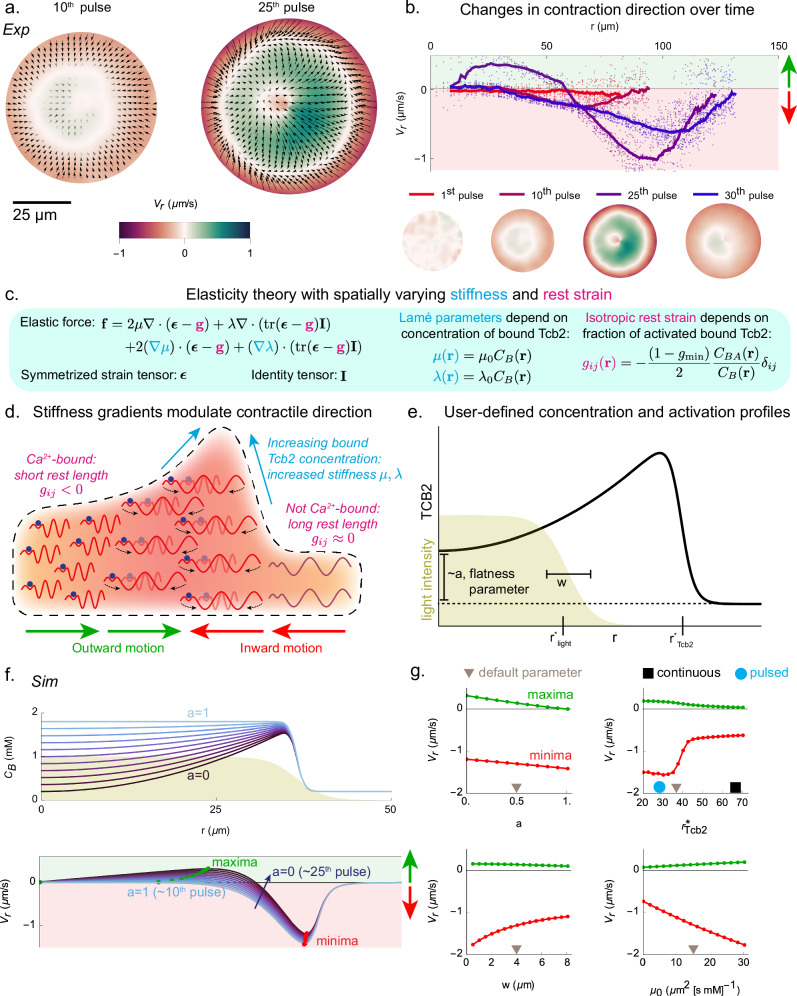
Reversal of contraction direction depends on inhomogeneous density. **a** Experimental PIV images at the 10th and 25th pulses. **b** *Top row*: Velocity profiles at the 1st, 10th, 25th, and 30th pulses plotted against radial distance. *Bottom row*: Experimental PIV fields at the corresponding time points, with the same velocity scale as in (**a**). We omit the arrows here for visual clarity and show only color. **c** Summary of the elastic theory used in our model (SI I E for more details). **d** Schematic illustration of the mechanism by which contraction direction reverses due to gradients of stiffness. See Fig. 1c for a legend of the molecular components. **e** Schematic depiction of the user-defined density profile used in the remaining panels. **f** *Top row*: Illustration of the bound Tcb2 profile as the user-defined flatness parameter *a* (which is small for large relative accumulation at the boundary) is varied. *Bottom row*: Radial velocity profiles during light activation for the corresponding density profiles in the top row. Qualitative correspondences to the pulses in (**b**) are indicated parenthetically. **g** The maximal and minimal radial velocities (green and red dots in **f**) plotted as *a* (the flatness parameter), ${r}_{\,Tcb2}^{*}$ (the boundary of the Tcb2 network), *w* (the width of the light profile), and *μ*_0_ (the slope of stiffness dependence on *C*_*B*_) are varied. Default values used in the plots are indicated with a brown triangle. The black square and cyan circle symbols suggest correspondences to the continuous and pulsed light protocol.

We observe that during network growth in the pulsed protocol the system not only contracts inward but also exhibits regions that contract radially outward, as shown in the PIV images from the 10th and 25th pulses in Fig. 4a. This is also illustrated in Fig. 4b, which further shows that at even later times (30th pulse) the contraction direction is uniformly radially inward again. This reversal of contraction direction is also observed in the simulations (Fig. 3d).

What causes the change in direction of radial contraction? Analysis of the simulations reveals that a key element is a relative accumulation of bound Tcb2 near the periphery of the growing network, which appears as a density profile that peaks near the network boundary. In SI Figs. S26 and S27, we show peaked experimental and simulated concentration profiles and demonstrate that boundary accumulation largely depends on the diffusion of Tcb2 inward across the CAR. These new Tcb2 monomers first have a chance to bind when they encounter the network periphery, leading to a buildup of density there. The dynamical implications of these density inhomogeneities can be understood by considering the new qualitative features of our mechanical model beyond standard linear elasticity, as summarized in Fig. 4c; see SI I E for details. The density of the material is inhomogeneous, which causes the first and second Lamé parameters *λ*(**r**) and *μ*(**r**) to be inhomogeneous as well, and the instantaneous rest strain tensor **g**(**r**) is also inhomogeneous due to uneven binding of Ca^2+^ to Tcb2. These two modifications produce additional contributions to the total elastic force beyond those in a homogeneous material, and these additional forces depend on spatial gradients in the material density and bound Ca^2+^ concentration. In the presence of a peak in the density of bound Tcb2, these additional forces can overcome the inward contractile force and cause the material to move up the gradient of material density, which is radially outward for interior parts of the network (Fig. 4d).

To illustrate this picture, we perform reduced simulations in which we turn off chemical reactions, impose a user-defined concentration profile of bound Tcb2 and light-patterned Ca^2+^ activation, and simulate the dynamics of contraction. This allows us to vary the width and accumulation of the Tcb2 profile as an input. The relevant parameters of this setup are illustrated in Fig. 4e (see SI I E for details). The parameter *a* represents how much accumulation of Tcb2 there is near the boundary of the network. We find that this parameter directly controls the positive component of the radial velocity profile, as shown in Figs. 4f,g, supporting the hypothesis that the relative accumulation of bound Tcb2 near the boundary underlies the reversal in contraction direction (see Fig. 2a and SI Fig. S26 for experimental observation of accumulation). We further find that as the radius of the Tcb2 network increases beyond the light illumination region, the positive component smoothly decreases to zero in magnitude. This explains why at later cycles in the pulsed protocol, when the network has grown well beyond the illumination zone, the network again contracts uniformly inward. We also find that, as the width of the light activation profile increases, weakening the gradients in rest length, the contractile velocities decrease in magnitude. Finally, increasing the proportionality constant *μ*_0_, which links the shear modulus to the bound Tcb2 density, results in a proportional increase in both positive and negative contraction velocities (Fig. 4g).

Relating these trends to the experiments, during the early pulsed cycles, the contraction direction is inward, as there is little relative accumulation near the boundary. At later cycles, there is appreciable relative accumulation at the boundary, which leads to regions of both inward and outward contraction. Because the network grows less quickly in the pulsed protocol than in the continuous one, it tends to overlap more with the illumination zone (shown schematically as blue circles and black squares in Fig. 4g). At late cycles, though, when the network has grown beyond the illumination zone, the contraction direction is predominantly inward again, as predicted by the model. These complex elastic forces suggest a rich design space in which density gradients and Ca^2+^ profiles can be dynamically sculpted to achieve fine-grained spatiotemporal control over the motion of, for example, particles interacting with the Tcb2 network. We next consider strategies for exerting such control.

### Leveraging Tcb2 network growth and contraction for active particle transport

**Fig. 5:**
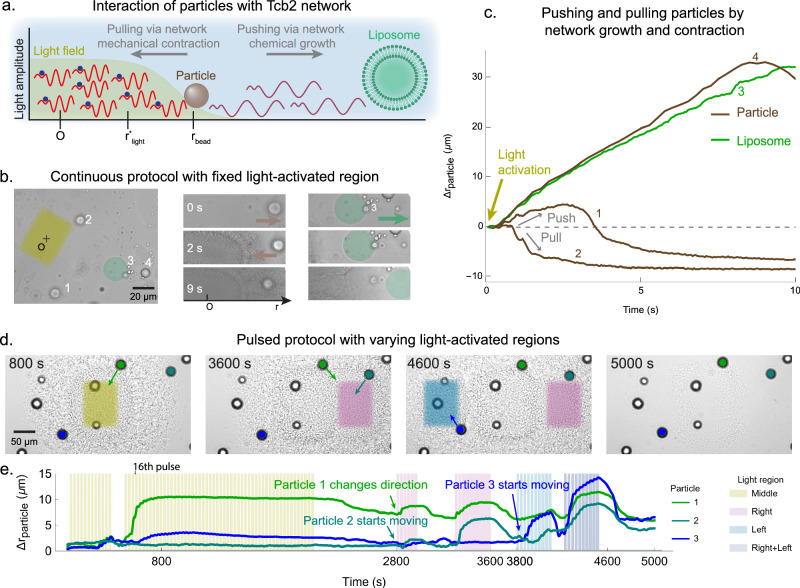
Controlling Tcb2 networks to move external particles. **a** Schematic illustration of light-controlled Tcb2 network interacting with embedded particles and liposomes. **b** Time-series images showing particle and liposome motion during continuous illumination of the yellow rectangular region. Particles 1, 2, and 4 are lipid particles and particle 3 is a liposome. **c** Displacement trajectories of three particles (brown) and one liposome (green) under continuous light activation, achieving outward displacements of up to 30 μm within 10 s. **d** Time-series images demonstrating particle motion as three rectangular regions are sequentially illuminated using a pulsed protocol. **e** Displacement trajectories of three tracked particles from (**d**) over 5000 s. Particle 1 demonstrates two directional changes—moving outward by  ~ 15 μm after the 16th cycle, then reversing rightward at 2800 s and leftward at 3800 s. Colored bands indicate activation periods: yellow (center), purple (right), blue (left), and dark blue (right + left) illumination regions.

To operationalize the pull and push of Ca^2+^-driven Tcb2 networks for active transport, we perform experiments in which the networks interact with liposomes, lipid particles (Figs. 5a,b) and polystyrene beads (SI Fig. S28 and SI Video Part 3 Section VII); see “Methods” for details on the liposome preparation. We conducted two experiments demonstrating active particle transport under different illumination protocols. First, we examine how far and how long the system can transport particles under continuous illumination. Upon illuminating a fixed region, the Tcb2 network grows outward and interacts with lipid particles. Some particles are pulled inward, others pushed outward, and some experience sequential push-pull motion (Fig. 5c and SI Section II L). This behavior depends on each particle’s initial radial distance from the illumination zone: particles near the zone become enmeshed in the rapidly forming Tcb2 network and are pulled inward as it contracts, while distant particles are sterically repelled by the expanding network periphery (SI Video Part 3 Section VII). The liposome moves outward by  ~ 30 μm within 10 s. We note that the rapid steric repulsion of the liposome soon after light activation is consistent with a model prediction of a lower-density peripheral network set up throughout the sample due to rapid initial Ca^2+^ diffusion upon first light exposure (SI II M). This peripheral network, at lower Tcb2 concentration than the primary network, does not give an appreciable DIC pattern. Quantifying its density should be accessible to rhodamine-based assays^72^, but we leave this for future work.

Second, we apply a pulsed illumination protocol to explore the system’s transport behavior across multiple illumination patterns. As shown in Fig. 5d and SI Video Part 2 Section VII, the illumination region is sequentially positioned at the center, right, and left of a sample containing embedded particles. Particle movement directly corresponds to the illumination location, demonstrating precise spatial control. Through reprogrammable light patterns, we transport multiple particles, achieving displacements of 5–15 μm with two directional changes, sustaining pulsed actuation for approximately 5000 s. For example, in Fig. 5e, Particle 1 reverses direction twice in response to shifts in the illuminated region, moving outward by approximately 15 μm after the 16th cycle and then reversing rightward at 2800 s and leftward at 3800 s. This demonstrates tight coupling between particle motion and activation position. Additionally, at these same time points (2800 s and 3800 s), we selectively activated particles 2 and 3, directing them toward the lower left and upper left, respectively. Across the field of view, particle response varied with proximity to the illuminated zone and pulse duration, revealing localized and tunable transport capabilities. By 5000 s, following a 500 s dark period, the system underwent near-complete chemical disassembly while retaining reactivation capacity. We observed small residual particle displacements after illumination cessation, suggesting viscoelastic behavior. Capturing these dynamics in our continuum model will require transitioning in the future from the current damped elastic solid rheology to a viscoelastic fluid description. We show additional particle movement statistics in SI Section II L and SI Fig. S28.

These applications of Tcb2 network growth and contraction to active particle transport suggest a diverse set of options for dynamical control over external objects, although we leave a full exploration of experimental control strategies to future work.

### Using reinforcement learning to control Tcb2 contraction

**Fig. 6:**
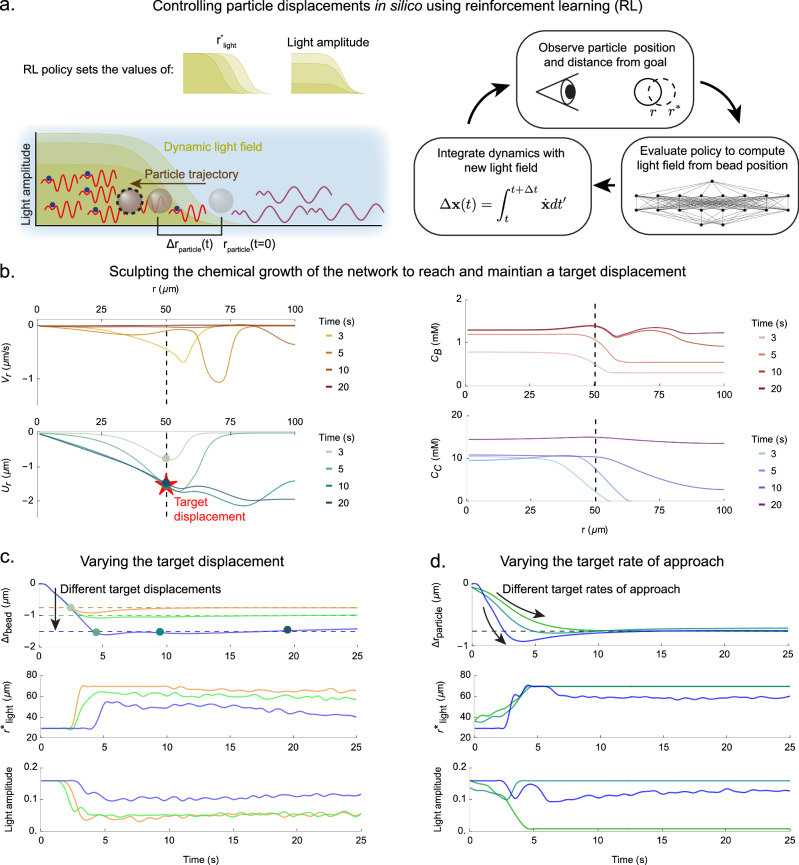
Using reinforcement learning to control displacements. **a** Schematic illustration of the control task considered here and the feedback loop for controlling particle displacements in silico using RL. The position and amplitude of a sigmoidal radial light profile is varied dynamically to cause the displacement at certain point to reach a desired value. **b** Plots of the radial velocity *V*_*r*_, radial displacement *U*_*r*_, bound Tcb2 concentration *C*_*B*_, and diffusing Ca^2+^ concentration *C*_*C*_ at different times during an episode. The trained RL policy shines light to sculpt these fields and cause the displacement at *U*_*r*_(*r*_0_ = 50 μm) to reach its target value of −1.5 μm, indicated by the red star. The dashed line throughout indicates the position *r*_0_ = 50 μm. **c** *Top row*: Trajectories of the displacements over time for three different dynamical control tasks targeting different displacements. *Middle row*: The policy choices for the light position corresponding to the trajectories in the top row. *Bottom row*: The policy choices for the light amplitude corresponding to the trajectories in the top row. **d** Same as (**c**), but for dynamical tasks targeting different rates of approach.

Finally, we test the feasibility in silico of closed-loop control for positioning particles using light-actuated Tcb2 networks. We consider a task inspired by the experimental application in Fig. 5 of displacing a particle at a specified location in the network (assuming cylindrical symmetry) by a target amount and then maintaining the particle’s position (Fig. 6a). Specifically, we target the time-dependent radial displacement *U*_*r*_(*r*_0_, *t*) evaluated at a fixed radial location *r*_0_ and time *t*. Recent work showed that proportional-integral control can stabilize the spatially averaged velocity in chaotic active nematic channel flow using spatially uniform light intensity^53^, illustrating the general feasibility of closed-loop control in active matter at micron scales. Our goal here, however, is particle positioning with multiple inputs and highly nonlinear input–output relationships. To this end, we employ reinforcement learning (RL)^76^, a data-driven approach suited to high-dimensional non-linear systems. Similar RL-based methods have been applied in silico to control dynamics in active nematics, flocking systems, and active crystals^56,77,78^.

As discussed more in the Methods and SI I F, we train an actor-critic policy using the deep deterministic policy gradient algorithm^76,79,80^. At each time step *t* the RL agent observes the radial displacement *U*_*r*_(*r*_0_, *t*) at a chosen location *r*_0_, and selects as actions the radius and amplitude of a sigmoidal, azimuthally symmetric light profile (Fig. 6a). The reward for training is constructed to minimize the deviation from a specified target *U*_goal_ in a manner mimicking an overdamped spring connecting *U*_*r*_(*r*_0_, *t*) to *U*_goal_. Training is performed in silico using our coupled reaction-diffusion-elasticity model. Even though the RL agent observes only a coarse description of the system state, it learns policies that solve different control tasks by sculpting the chemical concentration and velocity fields in time (Fig. 6b), with the effect of driving the displacement at the chosen location to distinct target values (Fig. 6c). The learned strategies typically apply high light amplitude initially to contract the network and move the particle to the target location, after which the light position and amplitude oscillate and drift to compensate for deviations from the target. In SI Fig. S10 we demonstrate that this feedback control is robust to simulated latency of up to at least 3 s between system observation and update of the control fields.

To further probe the capabilities of RL-guided control, we considered a more challenging task: driving the displacement to the target value at different rates of approach, mimicking overdamped springs with different stiffnesses. We studied a similar dynamical control task for active nematic defects in ref. ^56^. As shown in Fig. 6d, RL successfully solves this problem in simulated Tcb2 networks. The learned policies realize distinct approach rates to the same displacement, demonstrating rate-shaping in addition to set-point control. These results suggest that Ca^2+^-driven Tcb2 networks, despite their highly nonlinear and spatiotemporally history-dependent dynamics, are amenable to control using low-dimensional state and input spaces, and may in the near future be harnessed to exert precise mechanical forces at micrometer and second scales.

## Discussion

We have developed an experimental platform and accompanying continuum model to control the Ca^2+^-driven contractile dynamics of Tcb2 protein networks in vitro. By reconstituting these networks outside the ciliates in which they naturally occur, we decouple them from the cellular complexity and directly manipulate the chemomechanical processes of proteins homologous to those driving the fastest molecular motions known in biology^36,47^. Using a light-sensitive Ca^2+^ chelator we locally tune the Ca^2+^ concentration in the solution, allowing us to exert fine-grained spatiotemporal control over the network’s growth and contraction. The disparate timescales of these two processes—slow chemical growth and comparatively fast mechanical response—give rise to rich material dynamics. By periodically pulsing light, we can sustain large spatial areas of contractile response for ~150 cycles through a ratchet-like effect. Moreover, the repeated mechanical contractions reflect the slowly evolving concentration profile of the growing network, leading to surprising reversals of contraction direction due to strong density inhomogeneities. The dynamical malleability of this system presents an excellent opportunity for generating on-demand micron-scale forces, which we have explored both in vitro by moving lipid particles and in silico using reinforcement learning.

This material is different from reconstituted actomyosin or microtubule-kinesin networks in several ways. First, the Tcb2 networks are largely amorphous and isotropic, such that the direction of contraction is not restricted to local filament orientations^19^. Second, our system assembles rapidly (in seconds), compared to cytoskeletally-derived systems (minutes to hours)^71^. The contraction rate of Tcb2 networks is ~0.5 μm/s, which is higher than that of actomyosin networks driven by non-muscle myosin II (~0.05 μm/s)^21,81,82^ and comparable to the rate of muscle myosin II (~0.25 μm/s)^83–85^ and of mixed myosin IIA and myosin IIB networks (~0.4 μm/s)^86^. This system is slower than microtubule-based transport driven by kinesin under light activation, which reaches speeds of ~2.5 *μ*m/s^17,23,29,35^, and slower than synthetic moysin XI system^22,28^. See SI I B for a comparison to specific studies. We note that ultrafast contractile velocities observed in protists such as *Spirostomum ambiguum*, which can reach 0.1 m/s^36,42^, involves a fishnet arrangement of Ca^2+^-binding proteins homologous to Tcb2 with a secondary scaffolding protein^48^. In future work it may be possible to refine the in vitro system to account for these biological details to reach similar speeds. Third, this system has a minimal chemical composition, containing just three key components: Ca^2+^, which acts as an external chemical trigger distinct from ATP, competing chelator molecules, and Tcb2 protein. In contrast, in most reconstituted actomyosin networks there are several additional accessory proteins and crowding agents that are typically used to regulate actin polymerization and confine filaments to a surface^87,88^.

The physics of Tcb2 networks presents several opportunities for model development, unification, and control. The system’s mechanics are tightly coupled to its chemistry: diffusive network growth creates strong density inhomogeneities, and Ca^2+^ binding to Tcb2 alters the elastic rest length, necessitating extensions to standard elasticity theory. The reversal of contractile forces due to these density inhomogeneities is a general physical phenomenon that may explain contractility in other systems like cytoskeletal networks^89^. Additionally, the coupling of stress generation to chemistry in our system is reminiscent of earlier works on periodic swelling in synthetic polymer gels due to pattern-forming chemical reaction networks^2,3^, although these swelling motions are much slower. That pulsing the light activation allows for greater force generation through a recharging mechanism may be relevant to understanding the function of, for example, pulsatile dynamics in excitable RhoA-actin systems^90,91^. Finally, our efforts to control this soft material using reinforcement learning builds on a broader current research thrust of using control theory techniques to design policies that enable precise, on-demand force production in soft active matter systems^49,50,52,53,56^.

**Fig. 7:**
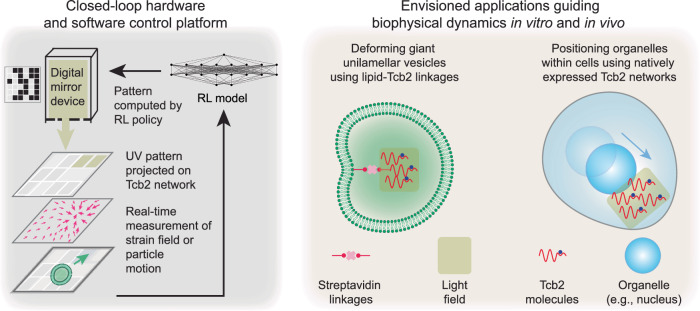
Future applications integrating hardware, software, and biology to control soft active materials. *Left*: Schematic illustration of an integrated experimental platform in which dynamical light patterning is controlled using real-time feedback from imaging of strain fields or particle displacements. A trained RL model or other control architecture maps imaging data to updates in the light field. *Right*: Possible applications of this control platform include deforming lipid vesicles with streptavidin-biotinylated Tcb2 networks, as well as manipulating organelle motion in vivo using light-controlled Tcb2 networks.

This demonstration of light-controlled Tcb2 network dynamics highlights its potential for programmable force generation in synthetic biology. Looking forward, we envision an integrated, closed-loop platform in which live imaging guides feedback control–potentially through reinforcement learning–to precisely manipulate soft active materials in vitro and in vivo (Fig. 7a). One promising direction involves biotinylated Tcb2: via streptavidin-biotin linkage, the network can be anchored to lipid membranes, enabling controllable membrane deformation and mimicking cellular processes such as cytokinesis or endocytosis^83^ (Fig. 7b). The same strategy could also be applied to tether Tcb2 networks to intracellular organelles to facilitate transport or remodeling. Additionally, the AM ester form of DMNP-EDTA can be loaded into live cells by incubation, allowing for localized Ca^2+^ modulation and enabling reconstitution of the system in a cellular context^92^. This offers a feasible approach for studying Tcb2-driven actuation under native-like conditions. The feasibility of light-guided feedback control has also been demonstrated in bulk active nematic flow systems^53^, further supporting potential implementations based on Tcb2 networks.

In parallel with these applications, the Tcb2 platform may also serve as a tool to uncover fundamental insights into how ultrafast contraction occurs in nature. Reconstituting such behavior in a minimal system could provide a mechanistic understanding of contraction in heterotrich protists. Moreover, these supramolecular protein networks could be used to construct synthetic cytoskeletal assemblies with capabilities distinct from those of microtubule- or actin-based systems. For example, they could introduce novel mechanical capabilities, including ultrafast contraction, localized force generation without the need for filamentous tracks, and light-controlled activation that is programmable and decoupled from ATP- or GTP-driven enzymatic pathways. These features open possibilities for intracellular actuation, sensing, therapeutic delivery, and the development of mechanically responsive components in synthetic cells.

## Methods

### Tcb2 construction, storage, and purification

We synthesize a codon-optimized full-length version of the Tcb2 protein and clone it into a pJ411 expression plasmid, which we then transform into the BL21 strain of *E. coli* (see SI I C for sequence details). We synthesize GFP- and mCherry-tagged variants of Tcb2 to facilitate experiments shown in Fig. 1a. To prevent aggregation during storage, we store the synthesized proteins in 4 M urea solution at  − 80 ^∘^C. Prior to experiments, we remove urea by overnight dialysis against a buffer containing 25 mM Tris (pH 7.5) and 1 mM EGTA, using a 3.5–5 kDa MWCO membrane (Spectra-Por Float-A-Lyzer G2, black). After dialysis, we concentrate the protein to approximately 30 mg/mL using a centrifugal filter (Amicon Ultra, UFC500324). We determine the final protein concentration by measuring absorbance at 280 nm with a UV spectrometer.

### Light control technique

To ensure complete chelation of Ca^2+^, we prepare a premixed solution of DMNP-EDTA and Ca^2+^ in a 2:1 molar ratio, with final concentrations of 0.54 mM Tcb2, 6.8 mM DMNP-EDTA and 3.4 mM Ca^2+^. We prepare the sample in a “sandwich structure" with a glass slide, a coverslip and a spacer (9 × 9 mm, 25 μL; BioRad, SLF0201) to maintain precise height. We perform imaging using a Nikon Ti2 Eclipse inverted microscope with a 20 × and 40 × objective. To initiate the release of Ca^2+^ from the DMNP-EDTA-Ca^2+^ complex, we project a 365 nm light pattern through a Mightex Polygon 1000-G DMD (SI I D).

### Liposome formation

We prepare liposomes using the cDICE method^93^ with EggPC (840051P, Avanti Polar Lipids) and an osmotic pressure difference of 10 mOsm/kg between the inner and outer solutions. The inner solution contains 290 mOsm/kg glucose, while the outer solution contains 300 mOsm/kg sucrose in ultrapure MQ water. We centrifuge the resulting aqueous phase containing liposomes at 1000 rpm for 5 min to concentrate the liposomes at the bottom of the sample due to the density difference between the inner and outer solutions. We then transfer the 2 μL aliquot from the concentrated liposome layer to a 30 mg/mL Tcb2 solution in a 10% v/v ratio, resulting in a total solution volume of 20 μL. We place the mixture on a glass slide that has been pretreated with a 10 mg/mL poly-L-lysine hydrobromide solution^25,94^. The lipid particles shown in Fig. 5b are the byproducts of liposome preparation^95^. We then prepare the sample in the same “sandwich structure” as described in Methods “Light control technique”.

### PIV analysis and particle preparation

We segment DIC videos using the AI model Segment Anything^96,97^ to track the growth of the Tcb2 network. We employ the prompt-based initialization model to enhance segmentation speed and accuracy for the Tcb2 network. For PIV analysis, we adapt the PIV MATLAB code to track Tcb2 filaments^98^, with images pre-processed using a Gaussian filter (standard deviation: 12 pixels), tracked using a nearest-neighbor algorithm within a search window grid of 48 pixels and post-validated using a correlation coefficient threshold of 0.6–0.8. We perform tracking and trajectory analysis of liposomes, lipid particles, and fluorescent particles using the AI tool Co-Tracker, with a manually set initial prompt-point and a grid size of 50 pixels^99^. We use 10–22 μm diameter Fluorescent Red Polyethylene Microspheres (cospheric UVPMS-BR-0.995) for SI Video Part 2 Section V.

### Continuum model

Our chemomechanical model uses reaction-diffusion dynamics for the evolution of the chemical concentration fields coupled to an overdamped elastic solid with viscous drag for the evolution of the displacement fields, whose density and rest strain depend on local chemical concentrations (see SI I E for details). To determine the motion of the system, we assume an instantaneous force balance between viscous drag and the local elastic force, which comprises several non-standard terms due to inhomogeneity of the Tcb2 density. The external light field is treated as a non-autonomous source function in the model whose magnitude spatiotemporally sets the rate of Ca^2+^ release by DMNP-EDTA. We distinguish between diffusing and bound Tcb2, as well as between Ca^2+^-bound and unbound Tcb2. As a first approximation, we assume a simple model for nucleation and growth of the Tcb2 network, in which available Tcb2 binding sites depend non-monotonically on the local concentration of bound Tcb2 to capture the competing effects of bound Tcb2 concentration locally creating new binding sites while also contributing to steric interference. In SI Fig. S8, we explore the effect on varying the saturation concentration of Tcb2 networks. Ca^2+^-induced growth of the bound network is modeled by taking the unbinding rate of Ca^2+^-bound Tcb2 from the network to be significantly less than that of Tcb2 not bound to Ca^2+^. We incorporate the degradation of DMNP-EDTA upon photolysis as a fractional contribution to the viable DMNP-EDTA concentration following Ca^2+^ release. For simplicity, we neglect possible advection of diffusing particles by motion of the network; in SI Fig. S9, we show that including advection makes little quantitative difference for this low (0.07−2) Péclet number system. To accelerate calculations for azimuthally symmetric light patterns, we solve the equations of motion in cylindrical coordinates and neglect the angular dependence. We constrain parameterization of the model by the known reaction rates and otherwise determine parameters through exploration to produce dynamics which match the experimental observables. We give parameter values in SI I E.

### Simulation methods

To integrate the dynamical equations, we developed custom Julia code which implements Heun’s method, a finite difference predictor-corrector scheme. We impose no-flux Neumann boundary conditions for the chemical species, preventing them from diffusing away from the simulation volume. We impose Dirichlet conditions **u** = **0** for the displacement field at the boundary. We choose sufficiently fine spatial and temporal discretization to ensure numerical stability and convergence.

### Reinforcement learning

We define the RL learning task in terms of an imposed dynamical law on the displacement *U*_*r*_(*r*_0_, *t*) at a fixed radial coordinate *r*_0_ of the material. We use RL to learn a mapping (called a policy) from *U*_*r*_(*r*_0_, *t*) to the radius of the azimuthally-symmetric sigmoidal light profile and its amplitude. This mapping should mimic the dynamics of an overdamped spring connecting the displacement at position *r*_0_ to the target displacement *U*_goal_. We vary *U*_goal_ and the overdamped spring constant to test the ability to learn different dynamical tasks, as in our previous work on active nematics^56^. We train the system using the deep deterministic policy gradient algorithm (DDPG), which is a variant of the actor-critic method^76,79,80^. For this, we combine our custom numerical integrator of the Tcb2 dynamics with the DDPG implementation provided by the Julia package ReinforcementLearning.jl^100^. Additional details of the RL problem formulation and training algorithm are given in SI I F.

## Supplementary information

Supplementary Information

Description of Additional Supplementary Files

Supplementary Movie 1

Supplementary Movie 2

Supplementary Movie 3

Transparent Peer Review file

## Data Availability

Data supporting this study are available in Zenodo (10.5281/zenodo.18318970^101^).

## Code availability

Code for modeling and reinforcement learning control of Tcb2 network contraction is available via Zenodo (10.5281/zenodo.18394283^102^).
