# Supplementary material for: Light-induced assembly and repeatable actuation in Ca2+-driven chemomechanical protein networks
Source: Nat Commun. 2026 Feb 21;17:3016. doi: 10.1038/s41467-026-69651-2 (PMC13035854; doi:10.1038/s41467-026-69651-2)
Supplement: Supplementary file 1 — Supplementary Information [file 41467_2026_69651_MOESM1_ESM.pdf]

Supplemental Material for:
Light-induced assembly and repeatable actuation in Ca^{2+}-driven chemomechanical protein networks

Xiangting Lei,[1,*] Carlos Floyd,[2,*] Laura Casas-Ferrer,[1] Tuhin Chakrabortty,[1] Nithesh Chandrasekharan,[3] Aaron R. Dinner,[2] Scott Coyle,[3] Jerry Honts,[4] and Saad Bhamla[1,5,†]

[1]*School of Chemical and Biomolecular Engineering, Georgia Institute of Technology, Atlanta, GA 30318*
[2]*Department of Chemistry and James Franck Institute, University of Chicago. Chicago, IL 60637*
[3]*Department of Biochemistry, University of Wisconsin Madison, Madison, WI 53706*
[4]*Department of Biology, Drake University, Des Moines, IA 50311*
[5]*BioFrontiers Institute and Department of Chemical and Biological Engineering, University of Colorado Boulder, Boulder, CO 80303*

CONTENTS

I. Supplementary methods 3
A. Biological background on Tcb2 and its homologs in ciliates 3
B. Comparison with other biomimetic contractile systems 5
C. Tcb2 construction and characterization 5
1. Protein sequence 7
2. Ca^{2+} concentration for assembly 7
D. DMD and optical setup 7
E. Continuum model 8
1. Model motivation 8
2. Equations of motion 10
3. Light field 14
4. User-defined concentration profile 14
5. Parameterization 14
6. Varying the model 15
F. Reinforcement learning 19
1. Problem definition 19
2. Algorithm 20
3. Latency 20
G. Area detection 20
1. Measuring area experimentally 20
2. Measuring area in simulation 21

II. Supplementary results 21
A. Varying the illumination area 21
1. Effect on the growth 22
2. Effect on the contractility 23
B. Pulse repeatability experiments over many cycles 25
C. Simulated concentration fields under continuous and pulsed protocols 26
D. Phases during a light pulse 26
E. Correlation of network and Ca^{2+} concentration profile 28
F. Comparison of mechanical contraction and chemical dissociation 28
G. Network growth requires degradation of chelator 30
H. Comparing the active region in light-on state under pulsed and continuous protocols 31
I. Varying the frequency of light pulses 32

---

* X. L. and C.F. contributed equally to this work.

† Correspondence to Saad.bhamla@colorado.edu

J. Varying the ratio of DMNP-EDTA to Tcb2 32
K. Boundary accumulation depends on diffusion of Tcb2 33
L. Spatially dependent particle transport
M. Formation of a peripheral network 35

References 37

I. SUPPLEMENTARY METHODS

A. Biological background on Tcb2 and its homologs in ciliates

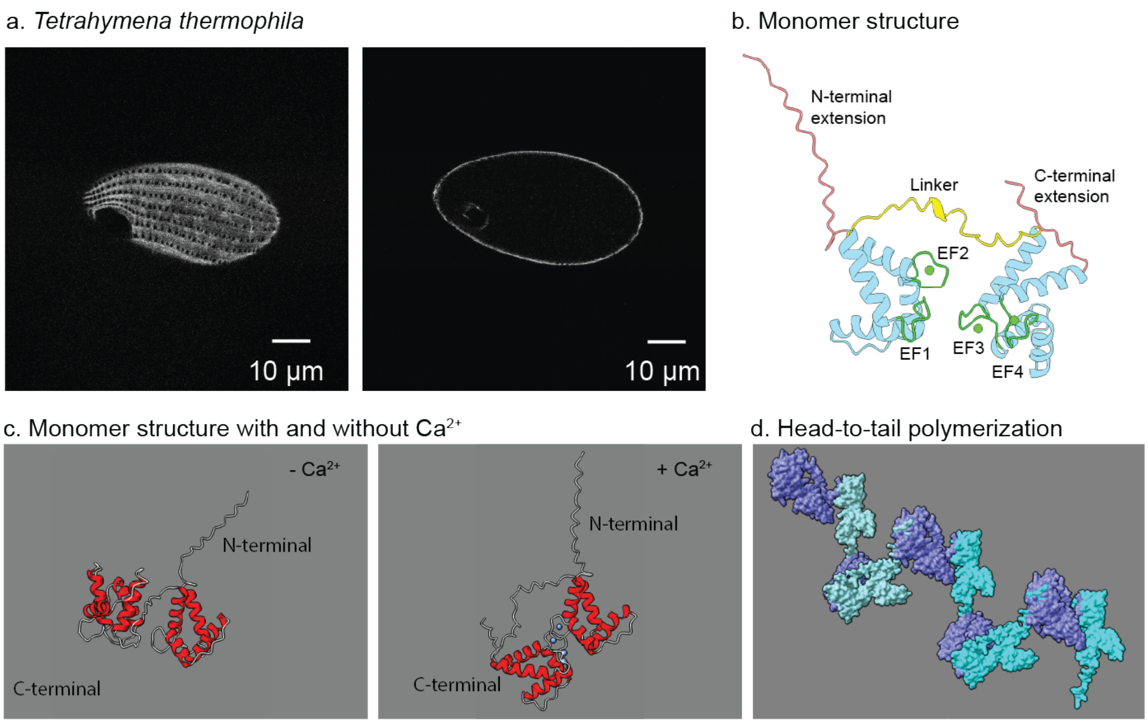

FIG. S1. ***Tetrahymena* and Tcb2 structure.** (a) Confocal laser scanning immunofluorescence microscopy images of *Tetrahymena thermophila* cell expressing a GFP-tagged version of Tcb2. The left panel shows the top plane, while the right panel captures the midway plane, highlighting the specific localization of Tcb2-GFP to the submembranous epiplasmic layer. (b) AlphaFold3 [1] prediction of Tcb2 monomer showing EF-hand domains (EF1 to EF4), disordered N-terminal extension, flexible linker and C-terminal extension. (c) AlphaFold3 prediction of Tcb2 monomer structure in the absence and presence of Ca^{2+}, illustrating structural changes upon Ca^{2+} binding. (d) AlphaFold3 prediction of Tcb2 polymerization with a head-to-tail connection.

Tetrahymena Tcb2, first designated as TCBP-25, is a 25-kDa Ca^{2+}-binding protein [2, 3]. It has been found to localize to the cortical cytoskeleton (epiplasm) of *Tetrahymena thermophila* cells [2, 4], which we also observe through fluorescence imaging *in vivo* in Fig. S1a. Tcb2 has been implicated in Ca^{2+}-mediated gametic pronuclei exchanged during mating and conjugation [3, 5]. It was also identified as a key component of a contractile gel formed from an alkaline low ionic strength extract of the *Tetrahymena* membrane skeleton, along with several other proteins including a high molecular weight protein Epc1 [6, 7]. We expressed a synthetic gene encoding Tcb2 in bacteria and discovered that the purified protein had Ca^{2+}-triggered contractile properties independent of its association with Epc1. Additionally, we found that a Ca^{2+}-responsive network can be reconstituted solely with Tcb2, leading us to focus specifically on the role of this protein.

Tcb2 is reported to have four potential Ca^{2+}-binding sites, along with a longer flexible linker between its N- and C-terminal EF-hand domains [3, 8]. Upon Ca^{2+} binding, the C-terminal domain of Tcb2 undergoes a substantial conformational change, as evidenced by NMR data [7]. Additionally, our predictions using AlphaFold3 suggest that

the C-terminal flips after binding to Ca^{2+} (Figs. S1b,c). Unlike calmodulins, Tcb2 forms filaments. In response to Ca^{2+} ions, Tcb2 assembles into a contractile network, as shown in SI video Part 1, Section I. Structurally, in addition to the longer linker already mentioned, Tcb2 also features an extended N-terminal structure and a shorter C-terminal extension (Fig. S1b). We note that these features are absent in calmodulin, another Ca^{2+} binding protein which is not thought to play a major structural or mechanical role.

Gel filtration chromatography suggests that Tcb2 forms polymers even in the absence of Ca^{2+}. Mass photometry supports this, further revealing that in the presence of Ca^{2+} there is a change in mass distribution indicating the formation of larger polymers. However, more research is required to determine the exact molecular role of Ca^{2+} in these processes. Here we propose two hypotheses on how Ca^{2+} reacts with Tcb2. First, Ca^{2+} binding may cause Tcb2 to assemble into longer filaments through head-to-tail assembly (Fig. S1d). Alternatively, Ca^{2+} may facilitate the aggregation of these filaments, leading to branching. However, it remains unclear whether Ca^{2+} binding drives the elongation of these polymers by adding more subunits or whether it promotes the formation of branching networks, as observed in fluorescence images in the presence of Ca^{2+} (Fig. 1 c,d). It is also possible that both mechanisms are involved.

**Ciliate species**	**Ca^{2+}-binding protein**	**Putative scaffold protein(s)**
Spirostomum [9]	centrin	Sfi1
Paramecium [10]	centrin	Sfi1
Vorticella [11, 12]	spasmin	spaconnectin
Tetrahymena [6, 7]	Tcb2	Epc1/Epa1

TABLE S1. **Comparison of Ca^{2+}-triggered contractile proteins in ciliates.**

Ciliates can utilize ATP-independent Ca^{2+} systems for motility, such as in the contractile stalk of *Vorticella* [11, 12] and the ultra-fast contraction of *Spirostomum* [17]. In these systems, contractility is hypothesized to result from a Ca^{2+}-dependent interaction between a Ca^{2+}-binding protein and an alpha-helical scaffold protein, commonly referred to as the "beads-on-a-string" model [18]. Centrins, EF-hand proteins, and associated alpha-helical scaffold proteins that contain tandem repeats of motifs are identified in Ca^{2+}-triggered ATP-independent contractile systems in *Spirostomum* [9].

Several centrin-like proteins have been identified in ciliates, as shown in Table S1 and Fig. S2. In *Paramecium*, centrin, together with the scaffold protein Sfi1, forms filaments within the infraciliary lattice that are hypothesized to coil or kink in response to Ca^{2+}, leading to contraction [18]. Similarly, the contractile stalk of *Vorticella* contains a related protein, spasmin, which requires an alpha helical scaffold protein called spaconnectin to form Ca^{2+}-sensitive contractile filaments [11, 12]. The centrin, spasmin, and Tcb2 proteins illustrate the diversity of Ca^{2+}-triggered contractility that has evolved in ciliates. In particular, unlike centrin and spasmin, subsequent bacterial expression of a synthetic gene encoding Tcb2 revealed that Tcb2 exhibits Ca^{2+}-triggered contractile properties independently of the Epc1 association.

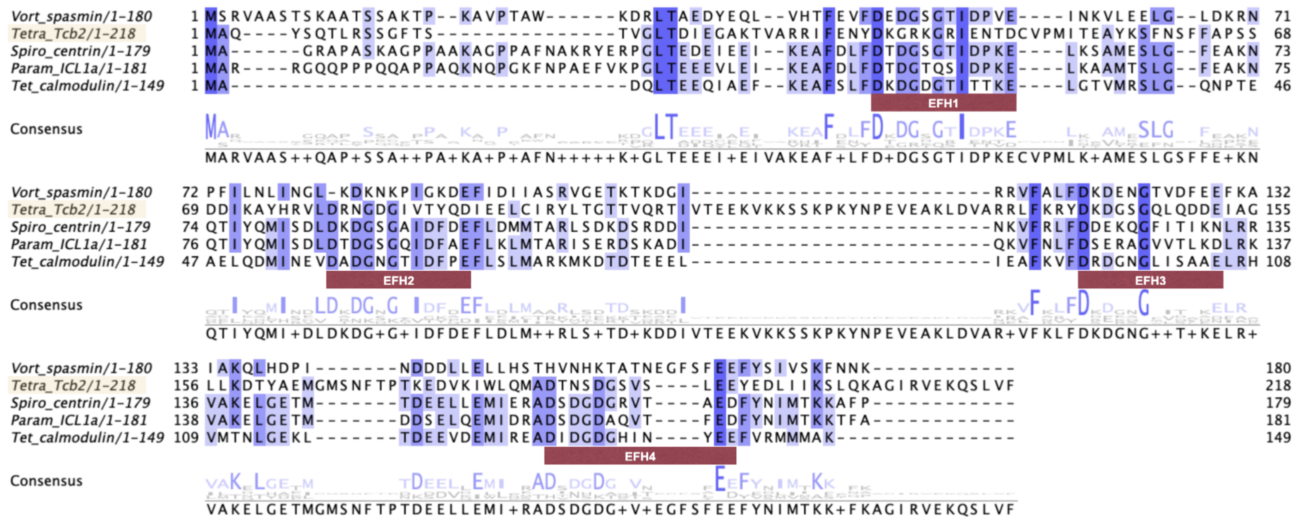

FIG. S2. **Homolog sequence comparison of Tcb2 in *Tetrahymena*.** Protein sequences observed or predicted to be involved in calcium-dependent contractile processes in ciliates and a ciliate calmodulin as a reference were retrieved from the National Center for Biotechnology Information. *Vorticella* spasmin: GenBank: AAD00995.1 [13]; *Tetrahymena* Tcb2: UniProtKB/Swiss-Prot: P09226.2 [14]; *Paramecium* ICL1a: UniProtKB/Swiss-Prot: Q27177.2 [15]; *Tetrahymena* calmodulin: PIR: S28954 [16]. The sequence of *Spirostomum* centrin is taken from our unpublished data. These sequences were aligned in Jalview using default settings for the MAFFT multiple sequence alignment server. Predicted EF-hand calcium-binding sites in Tetrahymena calmodulin, as predicted by PROSITE, are indicated.

B. Comparison with other biomimetic contractile systems

In Table S2, we compare two categories of cytoskeletal reconstitution systems: actomyosin systems (with both natural myosin motors and synthetic light-activated motors), and microtubule-kinesin systems (with both natural kinesin and synthetic light-activated motors). We note that in each case the reported velocity may correspond to slightly different measurements (such as filament motion, motor motion, or contractility rate), and we report these values as rough estimates of the typical system speed.

A key distinguishing feature among these system is whether they can be dynamically regulated. Some systems employ light-activated motors [19, 20] or light-activated inhibitors such as blebbistatin [21–25] that enable external control over contraction, allowing researchers to externally control motor activity. In contrast, many reconstituted systems lack such regulatory mechanisms and exhibit irreversible dynamics: once activated, they proceed uni-directionally though continuous contraction [26, 27], directional migration [22, 28] or turbulent-like flows [29] until their ATP fuel is exhausted.

C. Tcb2 construction and characterization

We cloned a synthetic Tcb2 gene (shown in I C 1), optimized for expression in *E. coli*, into plasmid pJ411 (high copy number, kanamycin resistance plasmid, with expression controlled by a T7 promoter), and transformed it into BL21 strain *E. coli* cells. We grew cells in lysogeny broth (LB) or super broth (SPB) media containing kanamycin, and

System	Maximal flow velocity	Motor	Dynamically regulated	Refs.
Actomyosin	0.06 μm/s	24 nM non-muscle myosin II	Yes (blebbistatin photocontrol)	[21]
Actomyosin	Continuous: 0.42 μm/s, Periodic: 3.33 μm/s	2.0 μM myosin IIA, 0.2 μM myosin IIB	Periodic waves	[26]
Actomyosin	0.3 μm/s	Skeletal muscle myosin II	No (irreversible)	[30]
Actomyosin	0.25 μm/s	Skeletal muscle myosin II	Yes (blebbistatin photocontrol)	[22]
Actomyosin	0.17 μm/s	Skeletal actin from chicken skeletal muscle	No (steady contraction)	[27]
Actomyosin	1 μm/s	Skeletal muscle myosin, or heavy meromyosin from rabbit muscle	Yes (blebbistatin photocontrol)	[23]
Actomyosin	4.8 μm/s	Non-processive heavy meromyosin	No (unidirectional motion)	[28]
Actomyosin	4 μm/s	Actin-intact cytoplasmic extracts from *Xenopus* eggs	Periodic oscillations	[31]
Actomyosin	0.9 μm/s (speed of transported lipid droplet)	Actin-intact cytoplasmic extracts from *Xenopus* eggs	No (irreversible, directional locomotion)	[32]
Actomyosin-rho	0.2 μm/s (speed of wave)	*Xenopus* embryos	Oscillatory waves	[33]
Actomyosin	2.5 μm/s (filament motion)	Synthetic (light-activated) myosin XI	Yes (optogenetic control)	[19]
Actomyosin	10 μm/s (filament motion)	Synthetic (light-activated) myosin XI	Yes (optogenetic control)	[20]
Microtubule-kinesin	2.2 μm/s	Kinesin	No (turbulent-like flows)	[29]
Microtubule-kinesin	1.8 μm/s	Kinesin	No (extensile dynamics)	[34]
Microtubule-kinesin	2.5 μm/s	Synthetic (light-activated) kinesin	Yes (optogenetic control)	[24]
Microtubule-kinesin	1 μm/s	Synthetic (light-activated) kinesin	Yes (optogenetic control)	[25]

TABLE S2. A comparison of flow rates, motor proteins, and dynamical regulation mechanisms across different cytoskeletal systems.

we induced expression by adding IPTG to 1 mM. We grew cells overnight at 18°C and harvested by centrifugation. We lysed cells using B-PER detergent solution and centrifuged the lysate at 15,000 g for 15 minutes. We extracted Tcb2 protein from the post-lysate pellet using 4M urea, 0.25 mM EGTA, 25 mM Tris-HCl, pH 7.5 buffer. We loaded the clarified extract onto a 25 mL Q-Sepharose anion exchange column and eluted with a NaCl step gradient in the presence of 4M urea. We concentrated and stored the Tcb2-containing fractions in urea-containing buffer at -80°C.

1. Protein sequence

We used the sequence of TcBP-25 in the National Center Biotechnology Information (NCBI) database, UniProtKB/Swiss-Prot: P09226.2 [14]. The sequence is (see also Fig. S2):

1 MAQYSQTLRSSGFTSTVGLTDIEGAKTVARRIFENYDKGRKGRIENTDCVPMITEAYKSFNSFFAPSSDD 69

70 IKAYHRVLDRNGDGIVTYQDIEELCIRYLTGTTVQRTIVTEEKVKKSSKPKYNPEVEAKLDVARRLFKRY 139

140 DKDGSGQLQDDEIAGLLKDTYAEMGMSNFTPTKEDVKIWLQMADTNSDGSVSLEEYEDLIIKSLQKAGIR 209

210 VEKQSLVF 218

2. Ca^{2+} concentration for assembly

We conducted two experiments to determine the critical Ca^{2+} concentration for Tcb2 assembly. We first performed a pelleting assay test. We mixed freshly diluted Tcb2 with a gradient of Ca^{2+} concentrations. At high centrifugal speed, the Tcb2 protein network pelleted (bottom sediment), while monomeric Tcb2 remained in the supernatant (top layer solution). We analyzed the concentration of Tcb2 in both the pellet and the supernatant using SDS-PAGE [35]. We observed a critical shift, where the bulk of Tcb2 moved from the supernatant to the pellet. The critical concentration for assembly is roughly 0.47 μM Ca^{2+}. In the second experiment, we monitored the progression of the Tcb2-Ca^{2+} reaction using a UV spectrometer at 280 nm, with a gradient of Ca^{2+} concentrations. We selected this wavelength based on prior determinations of Tcb2 concentration. Higher absorbance values indicate a denser Tcb2 network. We observed rapid and stable assembly at a critical concentration of approximately 4 μM Ca^{2+}.

D. DMD and optical setup

We customized our microscope by integrating a multi-port illuminator to enable 365 nm patterned illumination, while retaining the original light source for fluorescence. The components we use are listed in Table S3. The excitation filter was moved from the microscope's filter cube to the Multi-Port Illuminator. We use two dichroic mirrors (see the light path diagram in Fig. S3): a 450 nm shortpass filter reflects mCherry/green excitation light and transmits UV, allowing a combination of fluorescence and patterned illumination from the polygon projector, and a Multi-band Dichroic Mirror II transmits mCherry/green excitation light and 365 nm light toward the sample, while reflecting mCherry/green emission light from the sample to the camera. With this setup, the DMD pattern projector achieves sub-micron resolution with various patterns, as shown in Fig. S4. The smallest observed assembly size is approximately 20 μm $\times$ 20 μm.

To control Ca^{2+} release using the DMD, we use the photolyzable chelator DMNP-EDTA, whose chemical structure

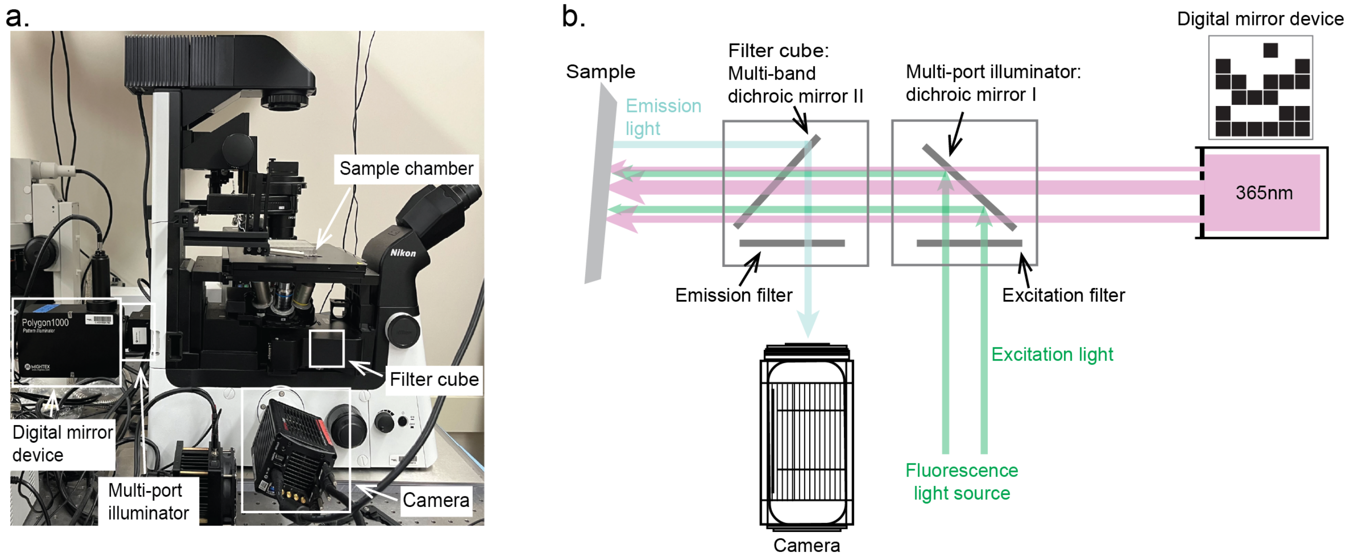

FIG. S3. **Experimental setup for patterned light illumination.** (a) Microscopy system with main components highlighted. (b) Illustration of the optical path with two main modules: the multi-port illuminator and filter cube alignment corresponding to panel a.

is shown in Fig. S5.

Throughout this paper, we performed comparative experiments on Tcb2 network assembly and contraction under a range of conditions. We performed these experiments with three to four replicates for each condition, analyzing distinct regions positioned at least 200-300 μm apart on the sample coverslip to ensure spatial separation for each sample. This ensured that the regions were not interconnected and minimized any potential effects of protein aggregates due to diffusing Ca^{2+}.

**Optical Component**	**Model**
Dichroic mirror I	Mightex FLTR-DCH-450S, Dichroic, 450 nm shortpass
Multi-band dichroic mirror II	Chroma 343448 (Reflection: 365 nm UV, 59022x (450–500 nm, 550–600 nm); Transmission: 59022m (500–550 nm, 600–650 nm)
Emission filter	Chroma 368163 (Transmission: 500-550 nm, 600-650 nm)
Excitation filter	Chroma 369288 (Transmission: 450-500 nm, 550-600 nm)

TABLE S3. **Optical components used in the experimental setup.**

E. Continuum model

1. Model motivation

We develop a physical model for the light-activated Tcb2 networks based on principles of reaction-diffusion chemistry and elasticity theory, with the goal of providing mechanistic explanations for several phenomena observed in our experiments. These include the localization of contractile force to the periphery of a continuously illuminated sample (main text Fig. 2d), a systematic heterogeneity of Tcb2 density along the radial direction (main text Fig. 2a and Fig.

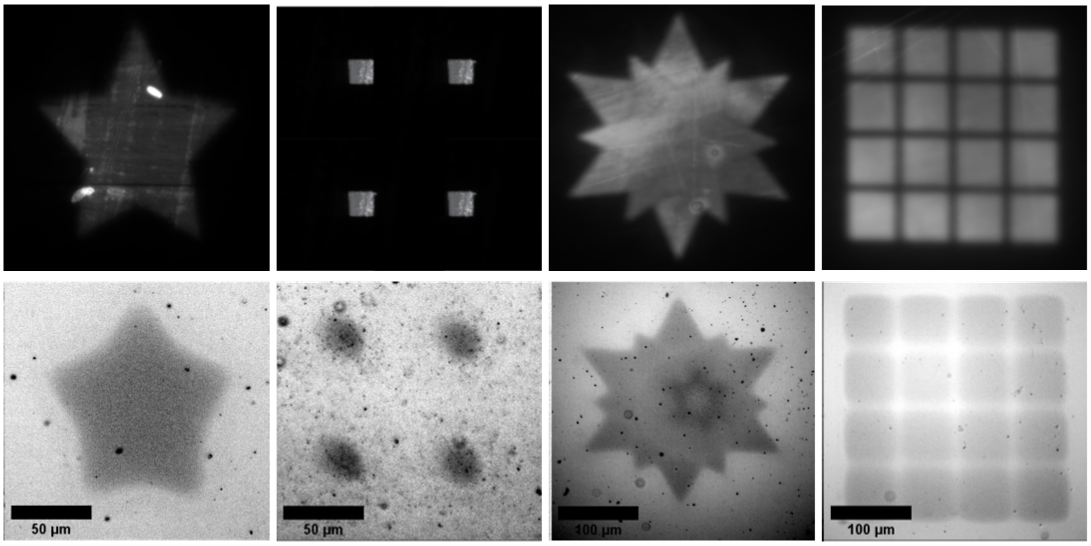

FIG. S4. **Patterned illumination.** *Top row*: A digital mirror device (DMD) projects various patterns onto a highlighter-coated mirror slide to visualize the UV pattern. We note that the uneven distribution of the light pattern is due to the highlighter strokes on the slides and visible scratches on the mirror, not the projector. *Bottom row*: The Tcb2 with DMNP-EDTA-Ca^{2+} system responds to the projected light patterns by forming protein assemblies with sharp boundaries, imaged using DIC.

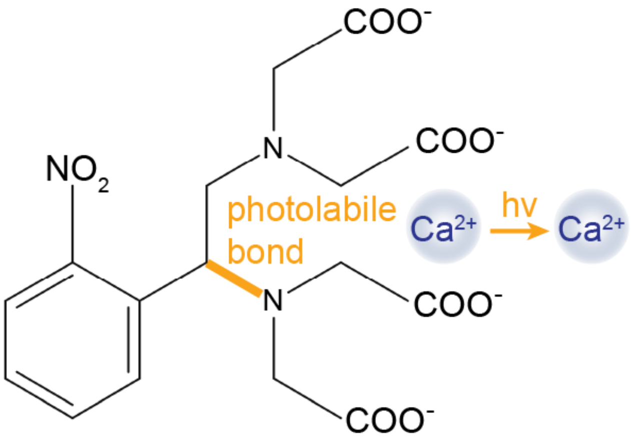

FIG. S5. **Chemical structure of DMNP-EDTA.** DMNP-EDTA has a photolabile bond which releases Ca^{2+} in response to light.

S26 below), the ability to repeatably recharge the network contraction upon pulsing the light rather than shining it continuously (main text Fig. 3d), and the surprising reversal of contraction direction from radially inward to outward at intermediate pulsation cycles (main text Fig. 4a). We find that a model based on first-principles considerations of the chemical reaction-diffusion dynamics and elasticity theory with a simple scaling between stiffness and network density suffice to recapitulate each of these phenomena. The model is not quantitatively accurate, however, largely due to a simplified treatment of network nucleation and growth, unaccounted-for influence of buffers in the solution, lack of treatment of viscoelastic effects and a general lack of experimentally constrained model parameters. We therefore view this model as a first iteration to be refined in subsequent work, but emphasize that it currently reproduces and

explains key experimental phenomenology as discussed in the main text.

2. Equations of motion

Here we outline the model equations of motion for a mixture of Ca^{2+}, Tcb2 molecules, and DMNP-EDTA chelators. We assume that diffusing Tcb2 molecules can bind to a network of bound Tcb2 filaments, and the bound filaments' mechanical rest lengths change upon Ca^{2+} activation. Ca^{2+} ions are sequestered by the available DMNP-EDTA chelators, and their release from these chelators is modulated by an externally applied light field. We track spatiotemporal concentrations of the following chemical species:

- Diffusing Tcb2 molecules in their inactivated (not Ca^{2+}-bound) state, denoted DI.
- Diffusing Tcb2 molecules in their activated (Ca^{2+}-bound) state, denoted DA.
- Bound Tcb2 molecules in their inactivated state, denoted BI.
- Bound Tcb2 molecules in their activated state, denoted BA.
- Diffusing Ca^{2+} ions, denoted C.
- Diffusing DMNP-EDTA molecules which do not contain a Ca^{2+} ion, denoted D^*.
- Diffusing DMNP-EDTA molecules which contain a Ca^{2+} ion, denoted D.

These species participate in the following reversible reactions:

- (In)activation of diffusing Tcb2 by Ca^{2+} (un)binding

$$DI + C \underset{k_{D,inact}}{\overset{k_{D,act}}{\rightleftharpoons}} DA \tag{1}$$

- (In)activation of bound Tcb2 by Ca^{2+} (un)binding

$$BI + C \underset{k_{B,inact}}{\overset{k_{B,act}}{\rightleftharpoons}} BA \tag{2}$$

- Trapping (release) of Ca^{2+} by DMNP

$$D^* + C \underset{k_{rel}(\gamma)}{\overset{k_{trap}}{\rightleftharpoons}} D \tag{3}$$

Here, $\gamma(\mathbf{r}, t)$ is a non-autonomous function representing a field of light that speeds up the released rate of DMNP-EDTA via the dependence $k_{rel}(\gamma)$.

- (Un)binding of DA to an open binding site O in the network

$$DA + O \underset{k_{A,unbind}}{\overset{k_{A,bind}}{\rightleftharpoons}} BA \tag{4}$$

- (Un)binding of DI to an open binding site O in the network

$$DI + O \underset{k_{I,unbind}}{\overset{k_{I,bind}}{\rightleftharpoons}} BI \tag{5}$$

The precise nature of how Tcb2 binds to other Tcb2 monomers to form the bound network is unknown, so we use a simple phenomenological model for how the concentration of available binding sites $O(C_B)$ depends on the concentration of bound Tcb2 $C_B \equiv C_{BA} + C_{BI}$. We take this function to be non-monotonic because of two competing effects: binding of Tcb2 to the network opens up new binding sites, and when there is too much locally bound Tcb2 then binding sites become sterically blocked. This steric hindrance is necessary to prevent a run-away positive feedback of binding, and it causes the concentration of bound Tcb2 to saturate at some level C_{sat}. We take the simple non-monotonic form

$$O(C_B) = \frac{C_{sat}^2}{4} - \left(C_B - \frac{C_{sat}}{2}\right)^2 \tag{6}$$

which is a downward facing parabola that intercepts $O = 0$ at $C_B = 0$ and $C_B = C_{sat}$ and has a maximum at $C_B = C_{sat}/2$.

The concentrations of these seven species are dynamical variables of the model, being functions of position $\mathbf{r}$ and time t. The dynamics of the diffusing molecules (DI, DA, C, D, D^*) have spatial dependence due to their concentration gradients.

To treat the linear elastic dynamics of the bound Tcb2 network, we introduce an additional vector field $\mathbf{U}(\mathbf{r}, t)$ and its derivative $\mathbf{V} = \partial_t \mathbf{U}$ which represent the displacement and velocity vectors of the network. The mechanical degree of freedom $\mathbf{U}$ is coupled to the chemical concentration variables in that the constitutive equation for the elastic deformation depends on the local chemical variables C_B and the fraction of Ca^{2+}-bound Tcb2 in the network $p_A \equiv C_{BA}/C_B$. Introducing the elastic stress tensor σ_{ij} (where the indices run over Cartesian directions x and y), the overdamped elastic dynamics have the force balance

$$\gamma_S V_i = \partial_j \sigma_{ij} \tag{7}$$

where γ_S is the Stokes drag coefficient. Introducing the symmetrized elastic strain tensor $\epsilon_{ij} = (1/2)\left(\partial_i U_j + \partial_j U_i\right)$, we use the isotropic elastic constitutive equation

$$\sigma_{ij} = 2\mu(C_B)\epsilon_{ij} + \lambda(C_B)\epsilon_{kk}\delta_{ij}, \tag{8}$$

where $\mu(C_B)$ and $\lambda(C_B)$ are the first and second Lamé parameters of the Tcb2 network. Because the network should be stiffer where it is denser [36], we assume that these elastic moduli increase linearly with local concentration of bound Tcb2:

$$\mu(C_B) = \mu_0 C_B \tag{9}$$

where μ_0 captures the slope of the dependence on C_B. We use a similar relation for $\lambda(C_B)$.

To account for the fact that Ca^{2+} activation of bound Tcb2 induces a change in the rest length of the Tcb2 molecules, we introduce an autogeneous (rest) strain $g_{ij}(p_A)$ which depends on the local proportion of activated Tcb2 molecules p_A as in our previous work [37]. We assume that this strain is isotropic, that is

$$g_{ij}(p_A) = \frac{g(p_A)}{d}\delta_{ij} \tag{10}$$

where $g(p_A) = g_{kk}(p_A)$ is the trace of g_{ij} in d dimensions. Using this, we update Equation 8 to

$$\sigma_{ij} = 2\mu(C_B)\left(\epsilon_{ij} - \frac{g(p_A)}{d}\delta_{ij}\right) + \lambda(C_B)\left(\epsilon_{kk} - g(p_A)\right)\delta_{ij}. \tag{11}$$

We take $g(p_A)$ to be a linear function between 0 (when $p_A = 0$, no autogeneous strain) and $-(1-g_{\min})$ (when $p_A = 1$):

$$g(p_A) = -\left(1 - g_{\min}\right)p_A. \tag{12}$$

Putting everything together into a closed set of dynamical equations for the variables C_{DA}, C_{DI}, C_C, C_D, C_{D^*},

C_{BA}, C_{BI}, and $\mathbf{u}$, we have

$$\partial_t C_{DI} = - k_{D,act} C_{DI} C_C + k_{D,inact} C_{DA} - k_{I,bind} C_{DI} O + k_{I,unbind} C_{BI} + \partial_i \left(D_{DI} \partial_i C_{DI} \right) \tag{13}$$

$$\partial_t C_{DA} = k_{D,act} C_{DI} C_C - k_{D,inact} C_{DA} - k_{A,bind} C_{DA} O + k_{A,unbind} C_{BA} + \partial_i \left(D_{DA} \partial_i C_{DA} \right) \tag{14}$$

$$\partial_t C_C = - k_{D,act} C_{DI} C_C + k_{D,inact} C_{DA} - k_{D,act} C_{BI} C_C + k_{D,inact} C_{BA} - k_{trap} C_{D^*} C_C + k_{rel}(\gamma) C_D + \partial_i \left(D_C \partial_i C_C \right) \tag{15}$$

$$\partial_t C_D = k_{trap} C_{D^*} C_C - k_{rel}(\gamma) C_D + \partial_i \left(D_D \partial_i C_D \right) \tag{16}$$

$$\partial_t C_{D^*} = - k_{trap} C_{D^*} C_C + \beta k_{rel}(\gamma) C_D + \partial_i \left(D_{D^*} \partial_i C_{D^*} \right) \tag{17}$$

$$\partial_t C_{BI} = - k_{B,act} C_{BI} C_C + k_{B,inact} C_{BA} + k_{I,bind} C_{DI} O - k_{I,unbind} C_{BI} \tag{18}$$

$$\partial_t C_{BA} = k_{B,act} C_{BI} C_C - k_{B,inact} C_{BA} + k_{A,bind} C_{DA} O - k_{A,unbind} C_{BA} \tag{19}$$

$$\partial_t U_i = \gamma_{\mathrm{S}}^{-1} \partial_j \sigma_{ij} \tag{20}$$

In Equation 17 we introduce the parameter β to control the degree of degradation of DMNP-EDTA chelators upon light-induced cleavage. If $\beta = 1$, then every molecule of DMNP-EDTA which release Ca^{2+} after photolysis is available again to bind a new Ca^{2+} ion. If $\beta = 0$, then every such DMNP-EDTA molecule is destroyed upon photolysis and hence does not enter the pool of available chelators.

The derivative $\partial_j \sigma_{ij}$ is

$$\begin{aligned}
\partial_j \sigma_{ij} =& \partial_j \left(2\mu_0 C_B \left(\epsilon_{ij} - \frac{g(p_A)}{d} \delta_{ij} \right) + \lambda_0 C_B \left(\epsilon_{kk} - g(p_A) \right) \delta_{ij} \right) \\
=& 2\mu_0 \left(\partial_j C_B \right) \left(\epsilon_{ij} - \frac{g(p_A)}{d} \delta_{ij} \right) + 2\mu_0 C_B \left(\partial_j \epsilon_{ij} - \frac{1}{d} \partial_i g(p_A) \right) \\
&+ \lambda_0 \left(\partial_j C_B \right) \left(\epsilon_{kk} - g(p_A) \right) \delta_{ij} + \lambda_0 C_B \left(\partial_i \epsilon_{kk} - \partial_i g(p_A) \right) \\
=& 2\mu_0 \left(\left(\partial_j C_B \right) \left(\epsilon_{ij} - \frac{g(p_A)}{d} \delta_{ij} \right) + C_B \left(\partial_j \epsilon_{ij} - \frac{1}{d} \partial_i g(p_A) \right) \right) \\
&+ \lambda_0 \left(\left(\partial_i C_B \right) \left(\epsilon_{kk} - g(p_A) \right) + C_B \left(\partial_i \epsilon_{kk} - \partial_i g(p_A) \right) \right).
\end{aligned} \tag{21}$$

The derivative $\partial_i g(p_A)$ is

$$\begin{aligned}
\partial_i g(p_A) =& \partial_i \left(1 - (1 - g_{\min}) \frac{C_{BA}}{C_{BA} + C_{BI}} \right) \\
=& (g_{\min} - 1) \frac{C_{BI} \partial_i C_{BA} - C_{BA} \partial_i C_{BI}}{C_B^2} \\
=& (g_{\min} - 1) \frac{(1 - p_A) \partial_i C_{BA} - p_A \partial_i C_{BI}}{C_B}.
\end{aligned} \tag{22}$$

For azimuthally symmetric light protocols, we convert these equations to cylindrical coordinates and solve for only the radial dependence.

Unless otherwise specified we use the spatially uniform initial conditions with $\mathbf{u}(\mathbf{r}, 0) = \mathbf{0}$. We set all concentrations to zero except $C_{DI}(\mathbf{r}, 0) = 1.25$ mM, $C_D(\mathbf{r}, 0) = C_{D^*}(\mathbf{r}, 0) = 20$ mM, and we uniformly nucleate the first growth of the bound Tcb2 network by setting $C_{BI}(\mathbf{r}, 0) = 0.05$ mM.

3. Light field

We decompose the non-autonomous function $\gamma(\mathbf{r}, t) = \gamma_r(\mathbf{r})\gamma_t(t)$, which mimics spatiotemporal light fields from the DMD, into a part depending only on position and a part depending only on time (Fig. S6). For circular profiles, the spatial part is a decreasing sigmoid function of the radial distance $r = ||\mathbf{r}||$,

$$\gamma_r(r; r^*, w) = \frac{1}{2}\left(1 - \tanh\left(\frac{r - r^*}{w}\right)\right), \tag{23}$$

having an offset r^* (the illumination radius) and a width w (the spread of light at the cutoff). Unless otherwise noted we set $w = 4$ μm throughout. The star pattern is implemented in 2D using a tunable parametric formula for the offset r^* as a function of polar angle. The time-dependent part $\gamma_t(t)$ is also constructed using sigmoidal curves with finite width of 0.5 s. In the pulse protocol we chain several sigmoidal bumps with pulse length of 1 s and a cycle length of 30 s, while in the continuous protocol we use a single bump with duration 100 s.

4. User-defined concentration profile

In Fig. 4 of the main text we show results using a user-defined concentration profile for $C_B(r)$ to systematically explore the role of boundary accumulation on reversing the contraction direction. The user-defined function is (cf. Equation 23)

$$C_B(r) = \gamma_r(r; r^*_{Tcb2}, 1)\left(a + (1 - a)\left(\frac{r}{r^*_{Tcb2}}\right)^2\right) + 0.2. \tag{24}$$

During simulations with this imposed concentration profile, we mimic the effect of light by directly setting the ratio $C_{BA}/C_B = \gamma_r(r; r^*_{light}, w)$ using Equation 23, and we then solve for the radial displacement fields $U_r(r, t)$ (cf. Equation 20) after a 1 s pulse of light.

5. Parameterization

In Tables S4, S5, and S6 we provide the default parameters used in simulation. We set the initial concentrations in Table S4 according to the experimental conditions. We constrained the reaction rates in Table S5 where possible

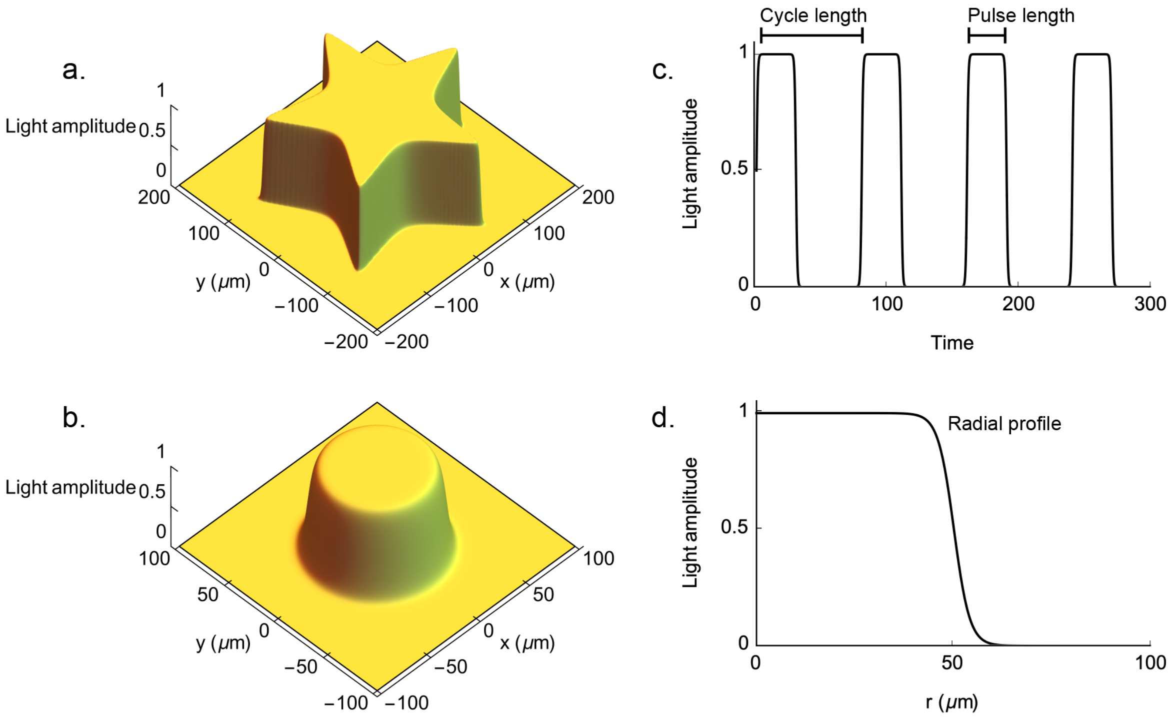

FIG. S6. **Light protocol.** (a) Spatial pattern $\gamma_r(\mathbf{r})$ of the star-shaped illumination used in simulation. (b) Spatial pattern $\gamma_r(\mathbf{r})$ of the circular illumination used in simulation, here with a radius of 50 μm. (c) Illustration of a pulsed temporal protocol $\gamma_t(t)$, with the cycle length and pulse length labeled. (d) For circularly symmetric illumination patterns, cylindrical coordinates are used and only the radial part of $\gamma_r(r)$ is considered. This profile corresponds to Equation 23 with $r^* = 50$ μm and $w = 4$ μm.

by available literature data, such as the DMNP-EDTA release rate of Ca^{2+} under light exposure [38, 39]. We set the unknown values by exploring parameter space to find reasonable agreement with experimental results. We estimated diffusion constants in Table S6 in order of magnitude using approximate knowledge of the size of the different molecules.

Parameter	Value	Units
C_C (diffusing Ca^{2+})	0	mM
C_{DI} (diffusing inactivated Tcb2)	1.25	mM
C_{BI} (bound inactivated Tcb2)	0.05	mM
C_{DA} (diffusing activated Tcb2)	0.0	mM
C_{BA} (bound activated Tcb2)	0.0	mM
C_D (DMNP-EDTA with Ca^{2+})	20.0	mM
C_{D^*} (DMNP-EDTA without Ca^{2+})	20.0	mM

TABLE S4. **Initial concentrations used in simulation.**

6. *Varying the model*

The continuum model is under-constrained by the available data. While future work will focus on kinetic parameter estimation, rheological measurements, and other characterizations to better constrain it, our present goal is not quantitative accuracy but rather to capture the key dynamical features observed experimentally, providing qualitative

Parameter	Value	Units
$k_{D,act}$ (binding of Ca to diffusing Tcb2)	50	$(\mathrm{mM}\cdot\mathrm{s})^{-1}$
$k_{D,inact}$ (unbinding of Ca to diffusing Tcb2)	20	s^{-1}
$k_{B,act}$ (binding of Ca to bound Tcb2)	50	$(\mathrm{mM}\cdot\mathrm{s})^{-1}$
$k_{B,inact}$ (unbinding of Ca to bound Tcb2)	20	s^{-1}
k_{trap} (trapping of Ca by DMNP-EDTA when light is on or off)	30*	$(\mathrm{mM}\cdot\mathrm{s})^{-1}$
$k_{rel}(0)$ (release of Ca by DMNP-EDTA when light is off)	0	s^{-1}
$k_{rel}(1)$ (release of Ca by DMNP-EDTA when light is on)	700*	s^{-1}
$k_{I,bind}$ (binding of inactivated Tcb2 to bound Tcb2)	0.2	$(\mathrm{mM}\cdot\mathrm{s})^{-1}$
$k_{I,unbind}$ (unbinding of inactivated Tcb2 from bound Tcb2)	0.5	s^{-1}
$k_{A,bind}$ (binding of activated Tcb2 to bound Tcb2)	0.2	$(\mathrm{mM}\cdot\mathrm{s})^{-1}$
$k_{A,unbind}$ (unbinding of activated Tcb2 from bound Tcb2)	0.5	s^{-1}
β (fraction of DMNP-EDTA returning to available pool after release)	0.2	–
C_{sat} (maximum concentration of bound Tcb2)	5	mM

TABLE S5. **Chemical reaction rates used in simulation.**
* - These values are based on Refs. 38, 39. To avoid stiff numerical integration steps, both the binding and unbinding rates have been decreased by the same factor (preserving their ratio) and keeping this process fast compared to others in the system.

Parameter	Value	Units
D_C (Ca^{2+})	300	$\mu\mathrm{m}^2/\mathrm{s}$
D_D (DMNP-EDTA with Ca^{2+})	100	$\mu\mathrm{m}^2/\mathrm{s}$
D_{D^*} (DMNP-EDTA without Ca^{2+})	100	$\mu\mathrm{m}^2/\mathrm{s}$
D_{DA} (activated Tcb2)	10	$\mu\mathrm{m}^2/\mathrm{s}$
D_{DI} (inactivated Tcb2)	10	$\mu\mathrm{m}^2/\mathrm{s}$

TABLE S6. **Diffusion constants used in simulation.**

Parameter	Value	Units
$g_{\min}$ (fractional change in rest length)	0.5	–
μ_0 (first Lamé parameter scaling factor)	15	$\mu\mathrm{m}^2/(\mathrm{mM}\cdot\mathrm{s})$
λ_0 (second Lamé parameter scaling factor)	15	$\mu\mathrm{m}^2/(\mathrm{mM}\cdot\mathrm{s})$

TABLE S7. **Mechanical parameters used in simulation.**

agreement that yields physical insight. In particular, the model recapitulates the localization of contractile force near the network periphery, the accumulation of bound Tcb2 near the periphery, and the occasional reversal of contraction direction toward the periphery from the inside. In this section we alter certain assumptions of our minimal model to verify that they do not change these qualitative results.

We first consider how the mechanical contraction dynamics depend on the manner in which stiffness (the Lamé parameters μ, λ) depends on Tcb2 density. Based on typical Ashby plots relating stiffness to material density [36], our simple assumption in the default model is that $\mu(C_B) \propto C_B$ and similarly for $\lambda(C_B)$. We generalize this to a power law dependence $\mu(C_B) \propto C_B^n$ such that at the saturation density $C_B = C_{sat}$ all stiffness models coincide at μ_{sat}. We thus consider the form

$$\mu(C_B) = \mu_{sat}\left(\frac{C_B}{C_{sat}}\right)^n, \tag{25}$$

and similarly for $\lambda(C_B)$. See SI Figure S7a for an illustration of this curve. We note that in the elastic equations of motion the spatial gradients of stiffness become more complicated using these power law models than in the default case when $n = 1$. We find different powers of n yield quantitatively different contraction dynamics, yet the qualitative features of the radial velocity curves (such as peaks near the boundary and presence of radially outward components) are preserved for each choice.

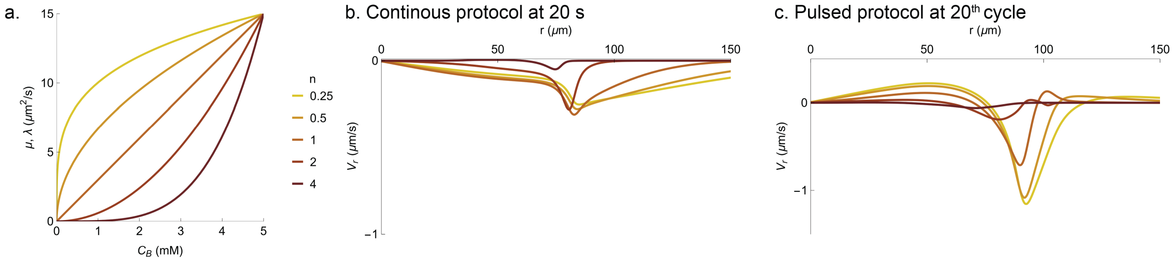

FIG. S7. **Comparison of simulated network dynamics for different values of n in the elastic model.** (a) Plots of the Lamé parameters μ and λ as a function of bound Tcb2 concentration C_B as n is varied among the values $0.25,\ 0.5,\ 1,\ 2,$ and 4. (b) Radial profiles of radial velocity V_r for simulations with different values of n for the continuous light protocol at 20 s. Colors correspond to the legend in panel a. (c) Same as panel b, but for the 20th cycle of the pulsed protocol.

Next, we test the effect of different values of the saturating bound Tcb2 concentration C_{sat}. Above this threshold, we assume that remaining Tcb2 binding sites are sterically inaccessible, causing the network binding rate to drop to zero. Although our phenomenological model for Tcb2 network nucleation and growth is simplified and will require refinement with improved data constraints, it is sufficient here to capture the essential features of growth via self-limiting aggregation. In SI Figure S8 we show that variations in this parameter around its default value of 5 mM yield negligible differences in the diffusing Ca^{2+} profiles (as Ca^{2+} binding by Tcb2 is taken to not depend on Tcb2-Tcb2 binding), and only minor quantitative differences in the profiles of bound Tcb2 and radial velocity.

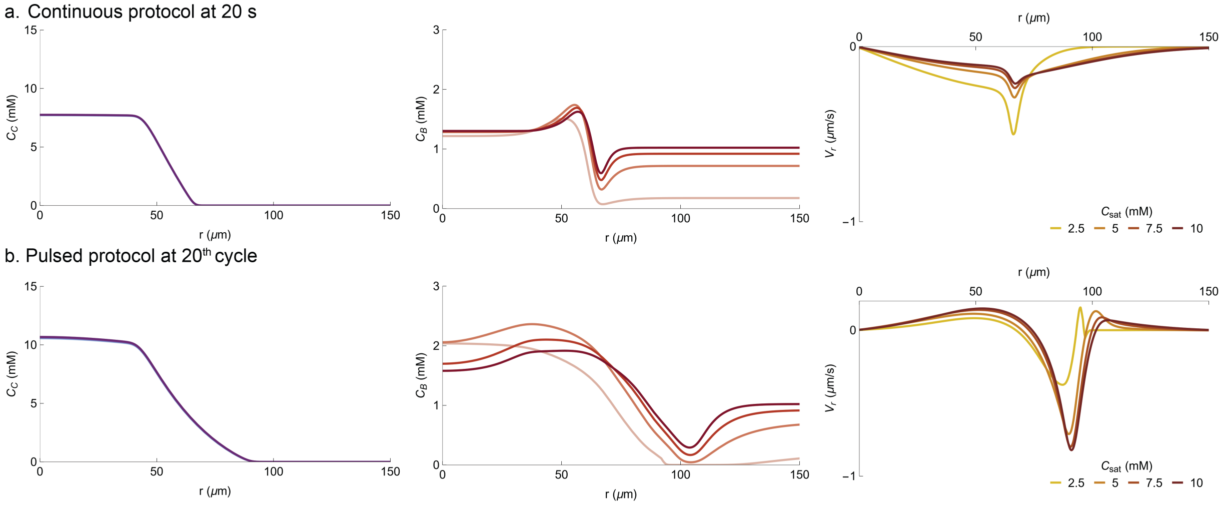

FIG. S8. **Comparison of simulated network dynamics for different values of C_{sat} in the chemical model.** (a) Radial profiles of diffusing Ca^{2+} concentration C_C, bound Tcb2 concentration C_B, and radial velocity V_r for simulations at 20 s during the continuous light protocol for different values of the parameter C_{sat}. (b) Same as panel a, but for the 20th cycle of the pulsed protocol.

Finally, we test whether advection of chemical species, which we neglect in the default model, has a significant effect. We run simulations with advective terms in the equations of motion for all chemical species and compare the resulting behavior with the default model which neglects advection. The results indicate that advection produces a small systematic correction to the diffusing Ca^{2+} and radial velocity profiles and negligible correction to the bound Tcb2 profiles, for both the continuous and pulsed light protocols; see SI Figure S9. Advection thus appears to not qualitatively affect the conclusions of the model.

To understand this, we estimate the Péclet number for the different diffusing species in our system (Ca^{2+}, DMNP, and Tcb2) using their diffusion constants of 300, 100 and 10 $\mu m^2/s$ respectively (these order-of-magnitude values are obtained based on molecular weights and are used in our simulations). The maximum contractile velocity is approximately 1 $\mu m/s$, and as a length scale we take the typical CAR width, which is roughly 20 μm. We thus have Péclet estimates of 1/15, 1/5, and 2 for Ca^{2+}, DMNP, and Tcb2. These values suggest that advection may introduce minor quantitative corrections to the observed dynamics but should not overwhelm the physics. Indeed this is what we observe when we include advection in the dynamics.

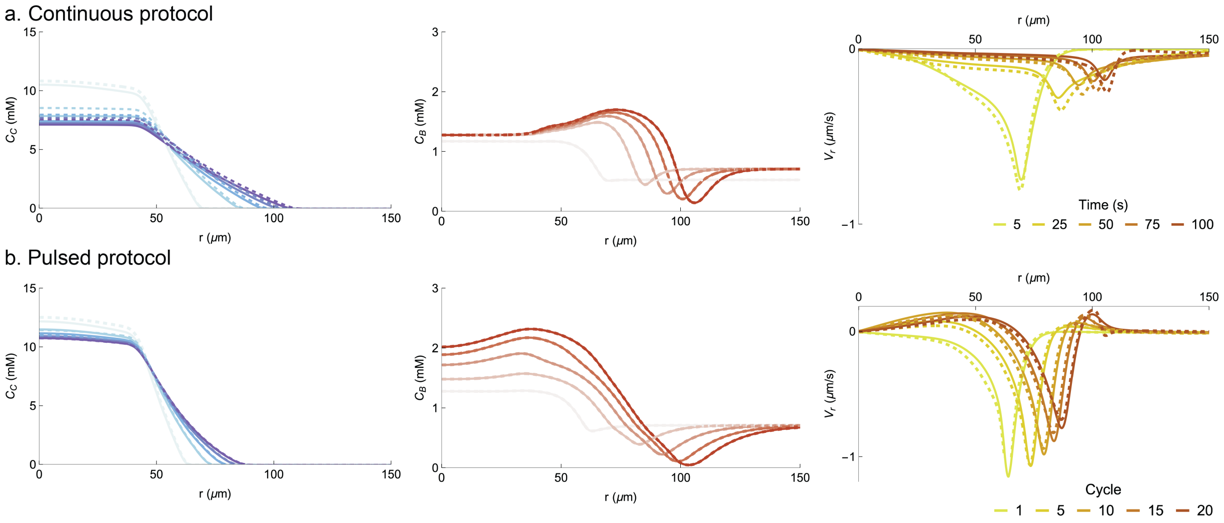

FIG. S9. **Comparison of simulated network dynamics with and without advection.** (a) Radial profiles of diffusing Ca^{2+} concentration C_C, bound Tcb2 concentration C_B, and radial velocity V_r for simulations with (dotted lines) and without (solid lines) the inclusion of advection in the dynamics for the chemical species. The colors range from light to dark as time increases during the simulation of the continuous light protocol, as indicated by legend on the right. (b) Same as panel a, but for the pulsed protocol.

We conclude that while the model lacks quantitative accuracy, its qualitative features of boundary accumulation, contraction localization, and contractile direction reversal are fairly robust to the modeling choices made here.

F. Reinforcement learning

1. Problem definition

Reinforcement learning (RL) is a set of techniques for training an agent to take actions in an environment so that it can optimize a reward function [40]. For us, the agent has control over the spatial light pattern which illuminates a Tcb2 network, and its goal is to move a certain spot in the network to a given position and then hold it there. Specifically, in radial coordinates we pick a position r_0 and imagine attaching a "spring" between the radial displacement at this point and a goal displacement U_{goal}. The "spring force" experienced at r_0 is $F_s = k_s(U_{\text{goal}} - U(r_0, t))$, where $U(r_0, t)$ is the displacement at coordinate r_0. Throughout this section all displacements are radial displacements but we drop the subscript r to simplify notation. We want the agent to shine light in a way that mimics the effect of this imaginary spring, thereby causing the point to move to position r^* and stay there.

The light field's spatial pattern is a sigmoidal decreasing function parameterized as in Equation 23. The width is fixed at $w = 4$ μm, and the agent has control over the amplitude $\gamma_t \in [0.01, 0.16]$ and offset $r^* \in [r_0 - 20, r_0 + 20]$ μm.

An episode of training is set as a fixed duration of time $T = 25$ s. The numerical integration time step is $dt = 2\times10^{-4}$ s, and the agent updates the light field parameters every 0.5 s. It chooses new parameters according to a neural network function which maps the state of the system, which it sees as $(U(r_0, t) - U_{\text{goal}})/U_{\text{goal}}$, into values of γ_t and r^*. We divide by U_{goal} so the result takes values of roughly order 1.

During an increment Δt, under the newly set of parameters chosen by the policy, the displacement will change to $U(r_0, t + \Delta t)$. If the point were really attached to a spring, then the change in U during this time would be

$$U_{\text{spring}}(r_0, t + \Delta t) - U(r_0, t) \approx \Delta t k_s(U_{\text{goal}} - U(r_0, t)) \tag{26}$$

where we have assumed overdamped dynamics for the spring with a drag coefficient that we absorb into the definition of k_s, which we set as $k_s = 0.01$ s^{-1} throughout. Because the agent is not perfectly imitating the imaginary spring, the real displacement will be $U(r_0, t + \Delta t) \neq U_{\text{spring}}(r_0, t + \Delta t)$. To encourage the agent to better imitate the spring, we thus provide rewards after each Δt as

$$\text{reward}(t) = -(U(r_0, t + \Delta t) - U_{\text{spring}}(r_0, t + \Delta t)^2. \tag{27}$$

This reward is used to provide updates to the function which maps the state $U(r_0)$ into actions, sigmoidal light amplitude $\gamma_t(t)$ and offset $r^*(t)$, so that the reward is eventually maximized. See Ref. 41 for additional details on our similar application of RL to control active nematic defects [42].

We emphasize that the agent only sees the current displacement $U(r_0, t)$ when deciding what light parameters to use, which is a very coarse view of the system state. That the agent nonetheless learns good policy functions given this information, at least for moderately long simulation trajectories, suggests that despite the complicated physics of

the full system, a coarse projection of the system state provides enough information to guide the system's dynamics.

2. Algorithm

To train the RL agent, i.e., learn a policy which maps $u(r_0)$ into light field parameters γ_t and r^*, we use a variant of the actor-critic algorithm [40] called deep deterministic policy gradient (DDPG) [43, 44], which is well-suited for continuous actions in deterministic environments. We combined our custom numerical integrator of the Tcb2 dynamics with the DDPG implementation provided by the Julia package ReinforcementLearning.jl [45]. The actor and critic neural networks each have 1 hidden layer with 32 neurons, which are trained using stochastic batches of 128 (state, action, reward, termination bool) tuples that are collected during training. The agent first chooses actions stochastically for $\sim 9,600$ steps after which it begins to use its learned policy. The neural networks are updated using stochastic batches of 128 tuples every 10th step. We use a discount factor $\gamma = 0.99$ and weight transfer factor $\tau = 0.995$ [44]. The neural networks are trained using the ADAM optimizer with a learning rate of 0.005 and a weight norm clip of 0.5.

3. Latency

To address robustness of the RL algorithm against common experimental challenges, we include *in silico* a latency between the current state of the system (when the actions are applied) and the RL agent's view of it. This mimics the finite processing time required to do image processing and evaluate the control policy in real time in our envisioned hybrid experimental and software platform (see Fig. 7 of the main text and Ref. 46). In Fig. S10 we show trajectories of trained policies for four levels of latency, including a severe latency of 3 s. In all cases the RL algorithm is able to learn a policy which brings the displacement to its target value, indicating that RL is a flexible enough approach to counteract latencies and learn effective feedback control.

G. Area detection

1. Measuring area experimentally

To segment the protein network from the background in the differential interference contrast (DIC) microscopy videos, we implemented the AI model Segment Anything [47] and used a modified version of the code from GitHub for video tracking [48]. To enhance both segmentation accuracy and speed for Tcb2 network (single-object) detection in DIC videos, we employed a prompt-based initialization method. We initialized the model with a prompt point at the center of the image, which is aligned with the center of the light activation area and the protein network. In the Supplementary Videos using this analysis, the red curve indicates the AI-detected protein boundary. We manually reviewed the videos to ensure the accuracy of the segmentation.

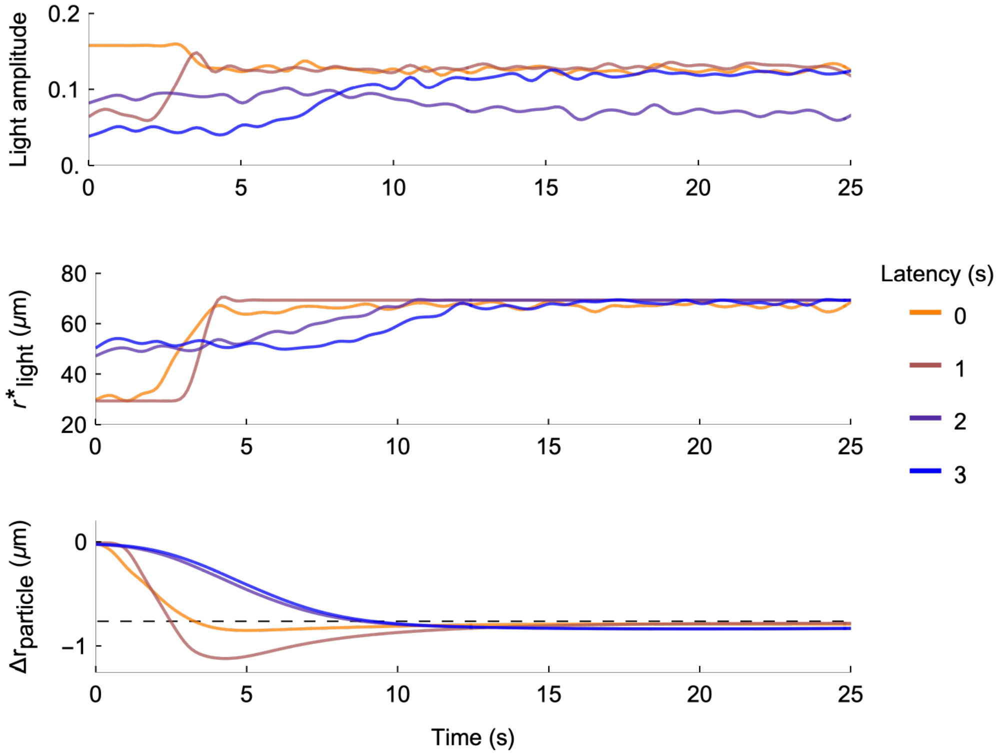

FIG. S10. **Including latency in the feedback control.** Trajectories of the light amplitude, light position, and displacement at the target location in the system for four levels of latency between the current state of the system and the RL agent's view of it.

2. Measuring area in simulation

To track the area of simulated Tcb2 networks, we defined a threshold on the simulated function $C_B(r,t) = C_{BA}(r,t) + C_{BI}(r,t)$ of total bound Tcb2. Using thresholds in the range $0.75 - 0.9$ μM, we define the radius of the network as the most distant value of r which exceeds the threshold. We note that this approach is different from the approach for tracking area in experiments, which does not have direct access to the value of $C_B(r,t)$ and relies on an adaptive AI method for segmentation. Despite these differences in approach, we find semiquantitative agreement between simulation and experimental results for the areal growth of the network.

II. SUPPLEMENTARY RESULTS

A. Varying the illumination area

Here we describe how properties of the network growth and contraction depend on the size of the circular illumination area, in both pulsed and continuous light protocols.

1. Effect on the growth

In Fig. S11 we show experimental and simulation results for how the network's growth in time varies with the illumination size using the continuous light protocol. We represent the network size using its effective radius R, obtained from its measured area A as $\sqrt{A/\pi}$. The networks grow faster with a larger illumination size in both the experiments and simulations.

We note that the simulations do not fully capture the degree to which the growth rate increases with the illumination size. Extensive parameter tuning failed to alleviate this disagreement, which we speculate may be due to an overly simplified model of the bound Tcb2 network's nucleation dynamics as well as unaccounted-for contributions from chemical buffers in the experimental solution. We leave resolution of this quantitative discrepancy as an avenue for future work, which may benefit from more dedicated measurements of the initial network formation.

The curves of effective radius $R(t)$ suggest that the growth of the network beyond the illuminated region is primarily determined by diffusion. The area covered by molecules diffusing from a source grows in time as $A(t) \propto Dt$ with D the effective diffusion constant. The corresponding radius thus grows as $R(t) \propto \sqrt{Dt}$. Accounting for an initial radial size (coming from the finite illumination region), we expect a diffusion-limited network's radius to grow in time according to the functional form

$$R(t) = a + b\sqrt{t}. \tag{28}$$

This functional form fits both the experimental and simulated growth curves reasonably well. In the experiments, the value a matches roughly with the initial illumination radius, although in simulations the growth occurs so rapidly in the beginning that the fitted value of a is larger than this illumination radius. Despite this difference between experiments and simulations, the fitted values of a both scale linearly as a function of illumination diameter and with similar slopes. The fitted values of b for experiments and simulations similarly differ in numerical values but scale in the same way with the illumination diameter.

In Fig. S12 we show experimental and simulation results for how the network's growth in time varies with the illumination size using the pulsed light protocol. As with the continuous protocol, the rate at which the network grows increases with illumination diameter. The growth rate averaged over cycles is approximately linear in both the experimental and simulated results, although we find that the simulations do not quantitatively match the slopes of these linear growth dynamics. For the initial cycles, the network does not accumulate enough bound Tcb2 to leave a detectable area, either through the adaptive AI-based segmentation method used to analyze experiments or through the fixed concentration threshold-based method to analyze simulation results. For the smallest illumination diameter in experiments, the network fails to nucleate a detectable area after many repeated cycles, suggesting that insufficient Ca^{2+} is released by the light illumination to persistently grow the network. We note that we repeated each experiment two to four times per condition but for clarity we only plot one trial per condition in Figs. S11, S12.

In Fig. S13 we show simulation kymographs of both the bound Tcb2 and Ca^{2+} radial profiles as a function of

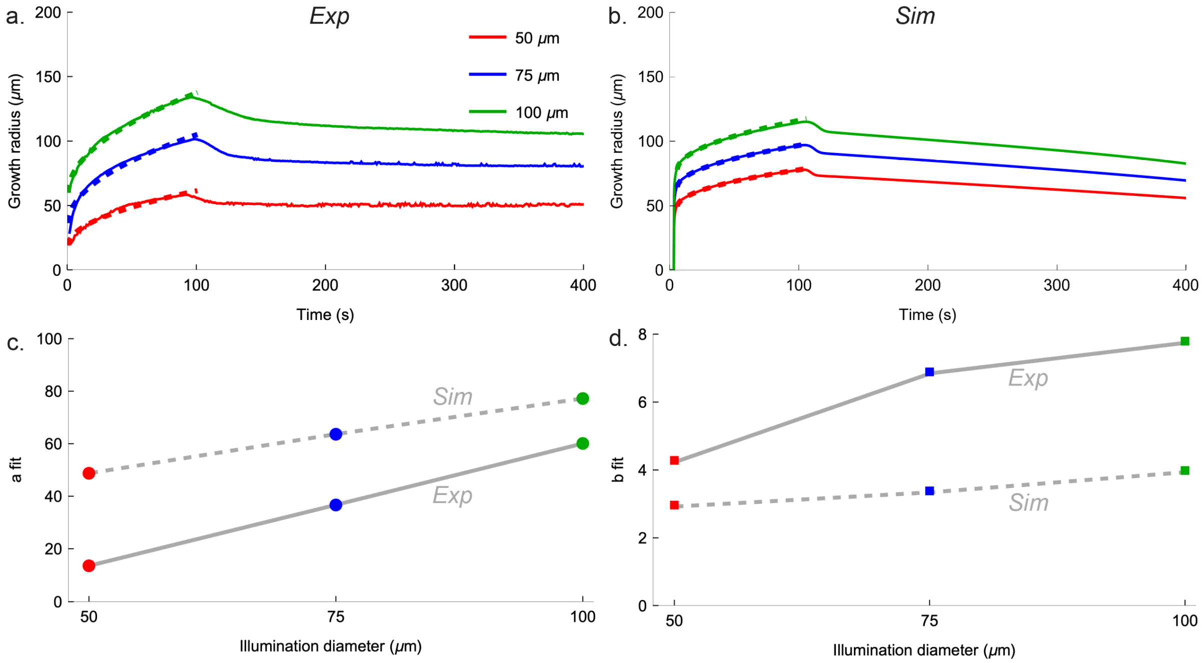

FIG. S11. **Network growth dynamics with varying illumination diameters for the continuous protocols.** (a) Experimentally measured growth radius as a function of time for different illumination diameters. Dashed curves indicate fits of Equation 28 to the 100 s of light being held on. We repeat each experiment 2-4 times but only plot one here for visibility. (b) Simulated growth radius as a function of time for different illumination diameters. (c) Fitted values of a in Equation 28. (d) Fitted values of b.

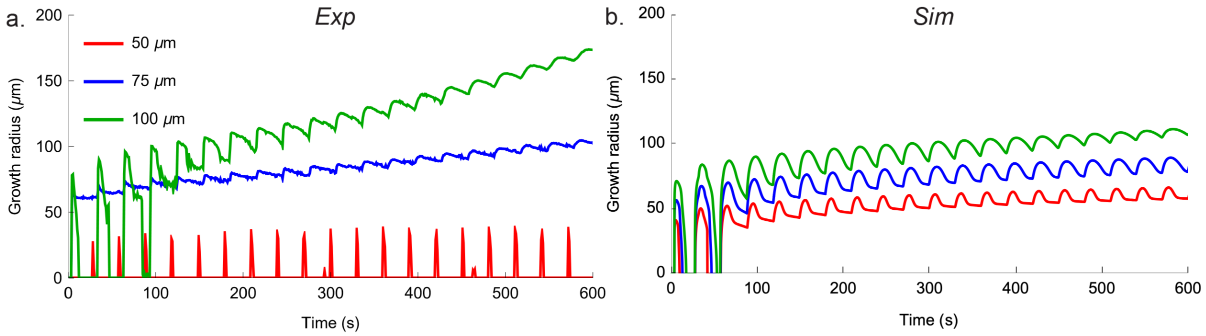

FIG. S12. **Network growth dynamics with varying illumination diameters for the pulsed protocols.** (a) Experimentally measured growth radius as a function of time for different illumination diameters. (b) Simulated growth radius as a function of time for different illumination diameters.

time across conditions of illumination diameter and light protocol. We observe qualitatively similar behavior as the illumination diameter is varied.

2. *Effect on the contractility*

In Fig. S14 we show experimental and simulation results for the network's radial velocity as a function of time, as we vary the illumination diameter and the light protocol. In experiments we observe qualitatively similar behavior of the network as the illumination size increases, with the exception of the smallest illumination size which fails to

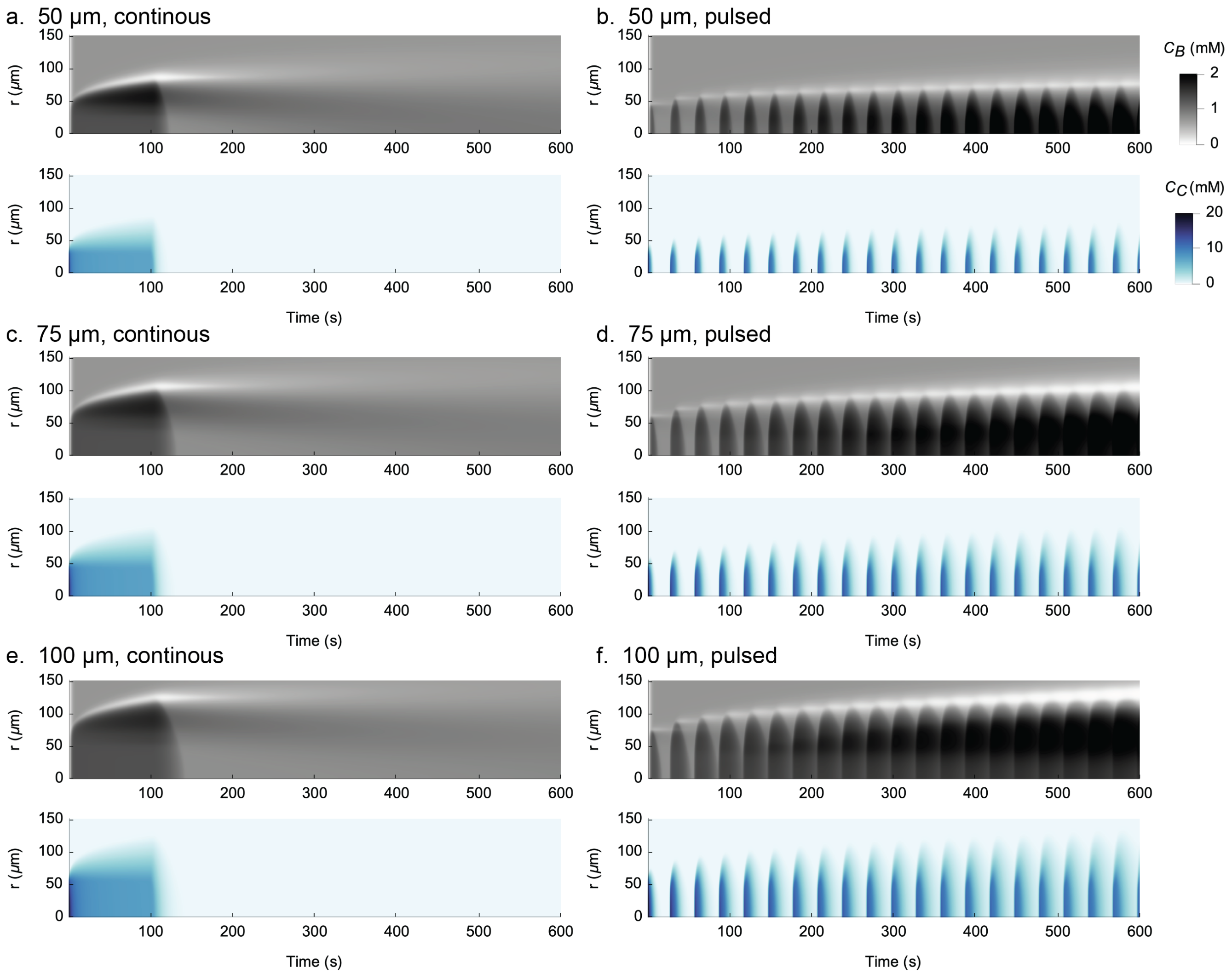

FIG. S13. **Network concentration dynamics with varying illumination diameters and light protocols.** (a) Simulated kymographs for the bound Tcb2 and diffusing Ca^{2+} concentrations as a function of radial position for the continuous light protocol, and with an illumination diameter of 50 μm. (b) Same as panel a, for the pulsed light protocol. (c) Same as panel a, for an illumination diameter of 75 μm. (d) Same as panel c, for the pulsed light protocol. (e) Same as panel a, for an illumination diameter of 100 μm. (f) Same as panel e, for the pulsed light protocol.

nucleate fully. The radial velocity data for this condition is noisy and we do not discern any clear trends.

We find slight discrepancies between the experimental measurement and simulation results for this data. We noted above that the network growth dynamics are not in complete alignment, leading to a difference in the spatial extent of radial velocity. In addition, we find in the experiments that during the continuous protocol the interior of the network has a small radially outward velocity for illumination diameters of 75 and 100 μm, while this feature is not observed in the simulations. We do observe radially outward velocities in the network's interior during the pulsed protocols, which we explain in Fig. 4 of the main text as resulting from an accumulation of bound Tcb2 concentration near the network periphery. Although this accumulation also exists in simulations of the continuous protocol, it does not give rise to an outward velocity. This suggests that further refinement of the model assumptions, such as the assumptions underlying nucleation and chemical growth, isotropy of the autogeneous strain due to Ca^{2+} activation,

linear growth rate of the Lamé parameters with C_B, or parameter choices are needed in the future to achieve more quantitative agreement with experiments. We leave these issues to future work, and note that the current model suffices to semi-quantitatively explain several other features of the contraction dynamics as described in the main text.

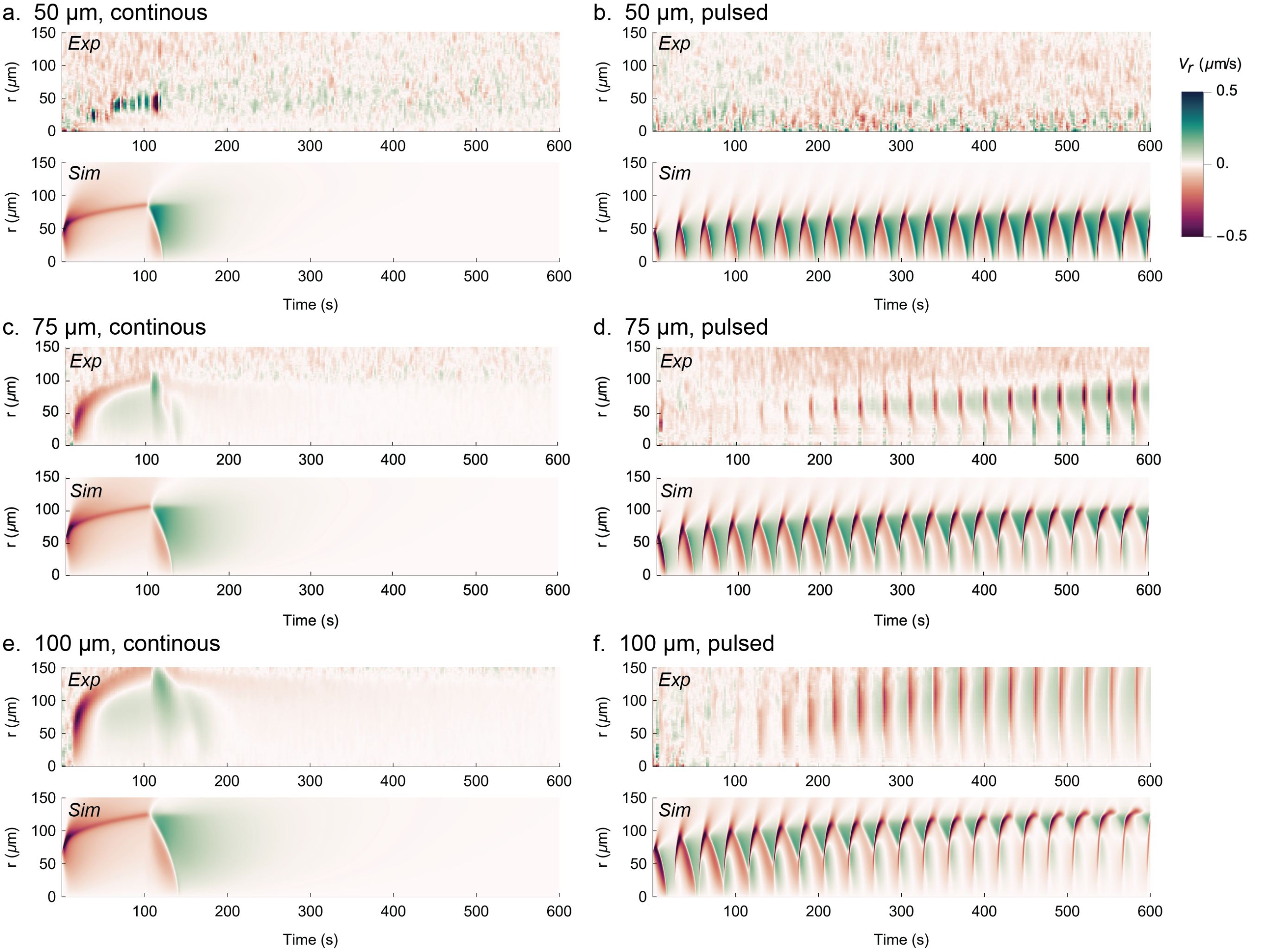

FIG. S14. **Network contraction dynamics with varying illumination diameters and light protocols.** (a) Experimental and simulated kymographs for the radial velocity as a function of radial position for the continuous light protocol, and with an illumination diameter of 50 μm. (b) Same as panel a, for the pulsed light protocol. (c) Same as panel a, for an illumination diameter of 75 μm. (d) Same as panel c, for the pulsed light protocol. (e) Same as panel a, for an illumination diameter of 100 μm. (f) Same as panel e, for the pulsed light protocol.

B. Pulse repeatability experiments over many cycles

To test the degree of repeatability of light-induced mechanical contraction, we performed experiments with large numbers of pulsation cycles. Figure S15 presents DIC kymographs (left) displaying chemical assembly and disassembly dynamics, alongside corresponding radial velocity-colored kymographs (right) that visualize contraction speed and amplitude. We performed cycling tests using illumination regions of three diameters: 50 μm ($\sim$150 cycles; SI Video

Part 2 Section VI), 75 μm ($\sim$70 cycles; SI Video Part 2 Section V), and 100 μm ($\sim$30 pulses). The maximum number of observable cycles was constrained by our 20$\times$ objective's 300 $\times$ 300 μm field of view, as contracting regions eventually extended beyond the imaging area. Figure S16 quantifies these dynamics through measurements of both the active contracting radius (defined as regions with velocities $>$0.2 μm/s) and average radial velocity within active regions. Following 150 cycles with 50 μm illumination, the active contracting radius stabilized at $\sim$30 μm at the periphery, maintaining a contraction speed of $\sim$0.4 μm/s. With 75 μm illumination, the radius expanded to $\sim$70 μm after 70 cycles while preserving a comparable speed of $\sim$0.4 μm/s. For 100 μm illumination, the radius reached $\sim$50 μm after 30 cycles, again demonstrating a consistent average speed of $\sim$0.4 μm/s.

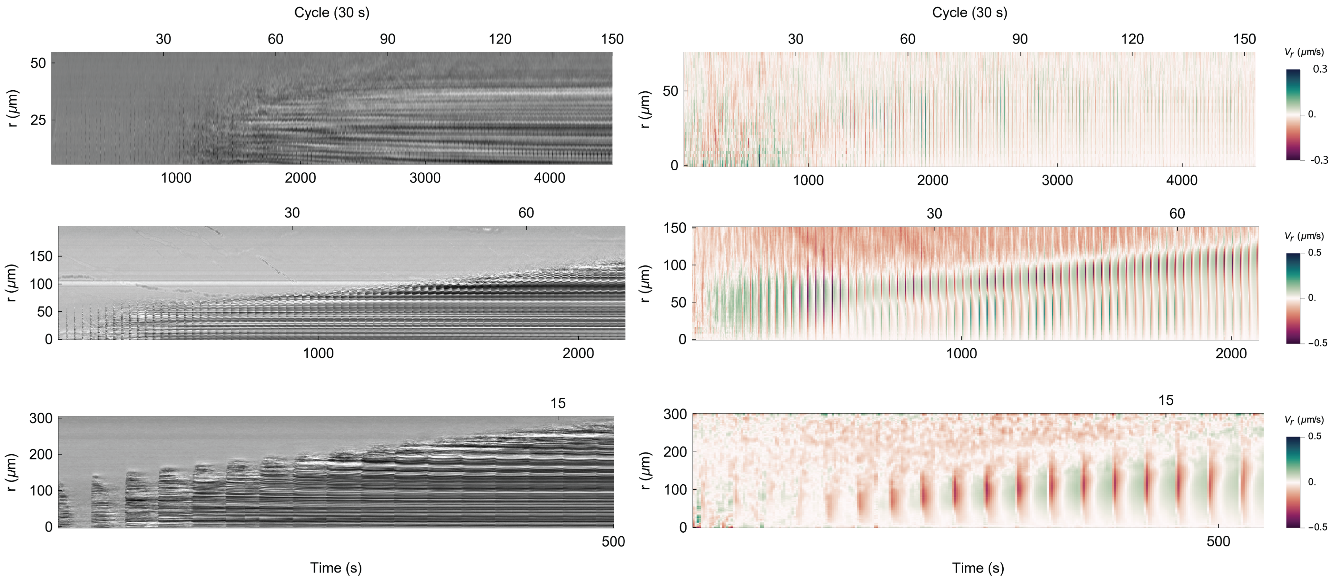

FIG. S15. **Repeatability tests under the pulsed protocol.** *Left*: DIC kymographs for illumination regions of 50 μm (150 cycles), 75 μm (70 cycles), and 100 μm (30 cycles) in diameter. *Right*: Corresponding radial velocity V_r kymographs.

C. Simulated concentration fields under continuous and pulsed protocols

In Figs. S17 and S18, we show the radial profiles of the concentrations for each chemical species tracked in simulation, for the continuous and pulsed protocols.

D. Phases during a light pulse

In Figure S19 we plot various physical fields during different phases of the 10th simulated pulse of light.

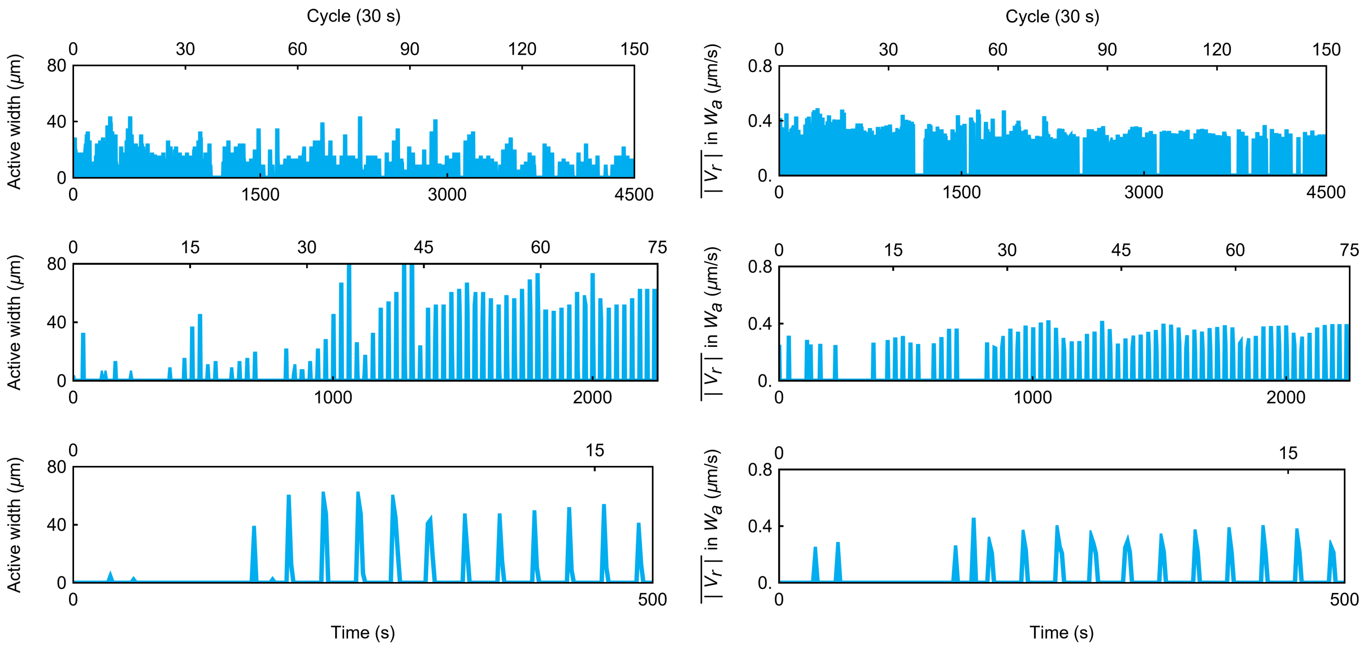

FIG. S16. **Repeatability of active width generation under the pulsed protocol.** *Left*: active width W_a for illumination regions of 50 μm (150 cycles), 75 μm (70 cycles), and 100 μm (30 cycles), defined as the radial extent where $|V_r| > 0.2$ μm/s. *Right*: mean radial velocity $\overline{|V_r|}$ within W_a across cycles.

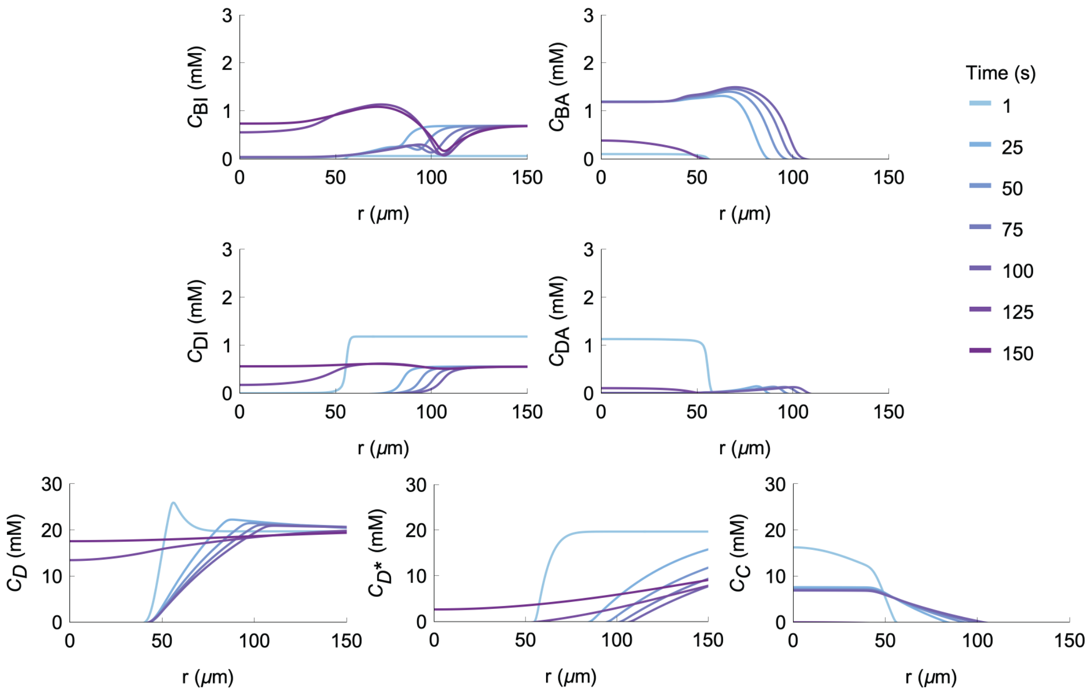

FIG. S17. **Concentration profiles during the continuous protocol.** Simulated concentration profiles for the various chemical species at different time points during the continuous protocol. The light is held on for 100 s and then turned off. The illumination diameter is 75 μm.

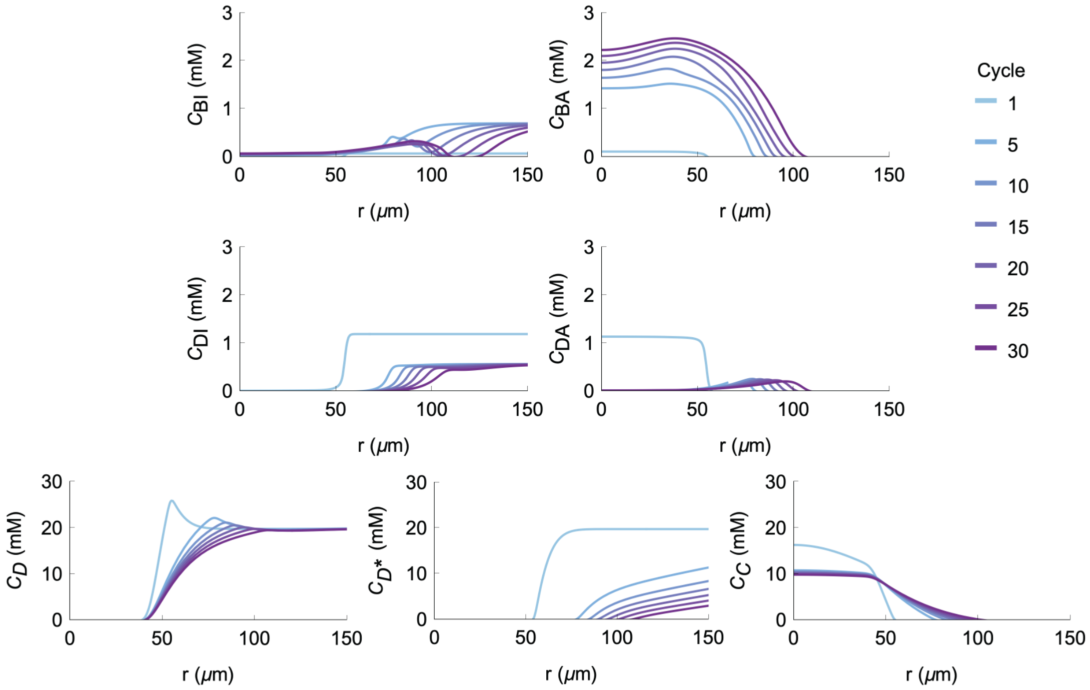

FIG. S18. **Concentration profiles during the pulsed protocol.** Simulated concentration profiles for the various chemical species at different time points during the pulsed protocol. The light is held on for 1 s and then turned off for 29 s for each cycle. The profiles are illustrated at the end of each 1 s light-on pulse. The illumination diameter is 75 μm.

E. Correlation of network and Ca^{2+} concentration profile

Ca^{2+} underlies network growth and contraciton in the Tcb2 network. Unfortunately in this study we are not equipped to perform quantitative calibration using the ratiometric method to measure Ca^{2+} concentration, and we leave this measurement for future work. Instead, we conducted a quantitative characterization of the spatial distribution at 340 nm using Ca^{2+} indicator rhodamine-2. In Fig. S20, we present experimental fluorescence images immediately after light activation and after 30 s of activation. A persistent bright spot is visible in the illuminated region at both time points. By the later time point, a more diffuse halo of fluorescence is also apparent, representing the diffusion of Ca^{2+} away from the illuminated region. Simulation results, colored to match the grayscale of the experimental images, resemble the experimental images.

F. Comparison of mechanical contraction and chemical dissociation

In Fig. S21 we highlight the disparate timescales of fast mechanical contraction at the active boundary and slow chemical dissociation of the network.

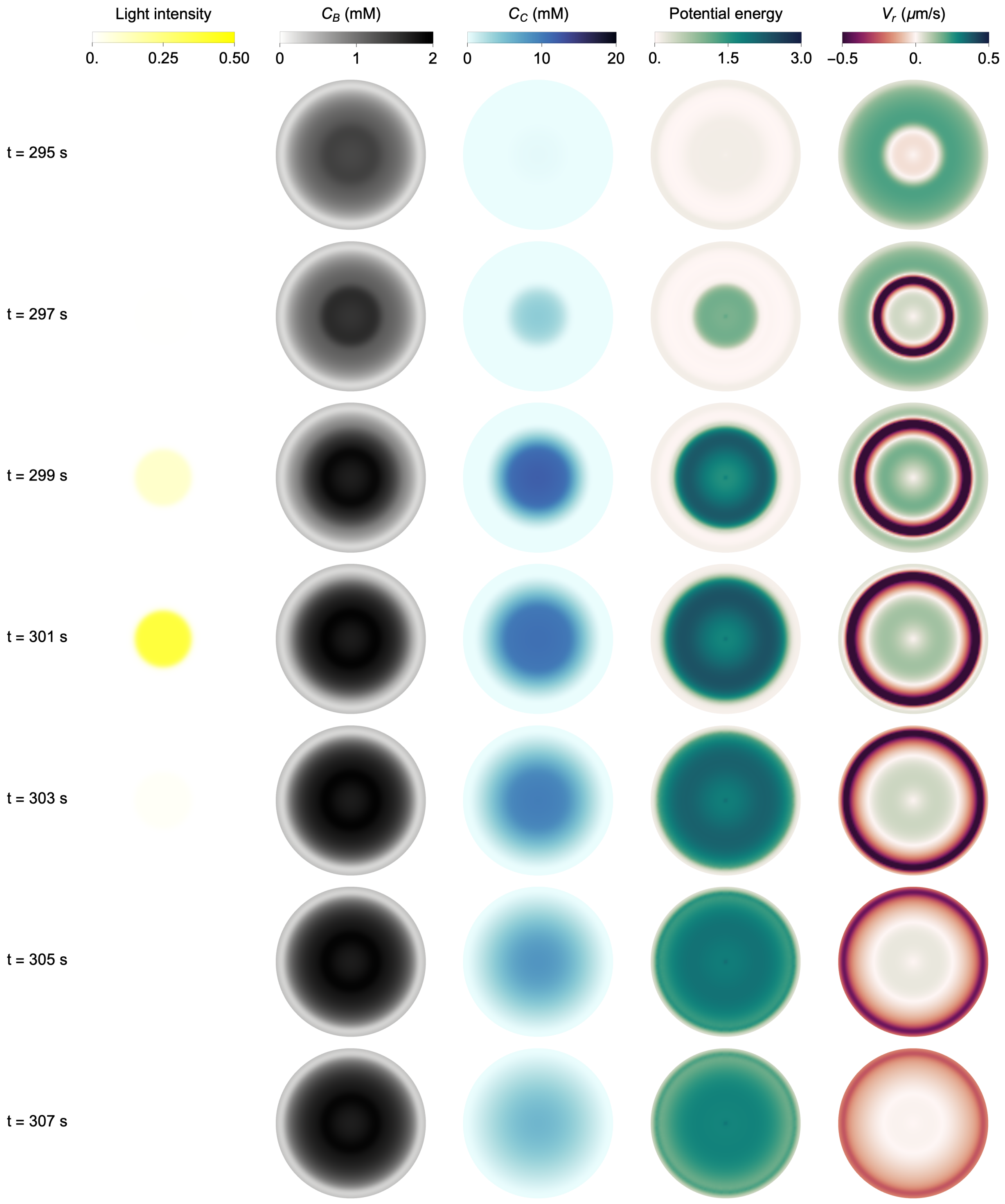

FIG. S19. **Phases of various fields during a pulse of light.** During the 10th simulated pulse of light, various physical fields are plotted at different phases. The mechanical potential energy density is computed as $\mu_0 C_B(\mathbf{r})\,(\partial_r u(\mathbf{r}) - g(\mathbf{r})/2)^2 + \frac{1}{2}\lambda_0 C_B(\mathbf{r}))\,(\partial_r u(\mathbf{r}) - g(\mathbf{r}))^2$. The diameter of each visualization domain is 200 μm, and the diameter of the light pulse is 75 μm.

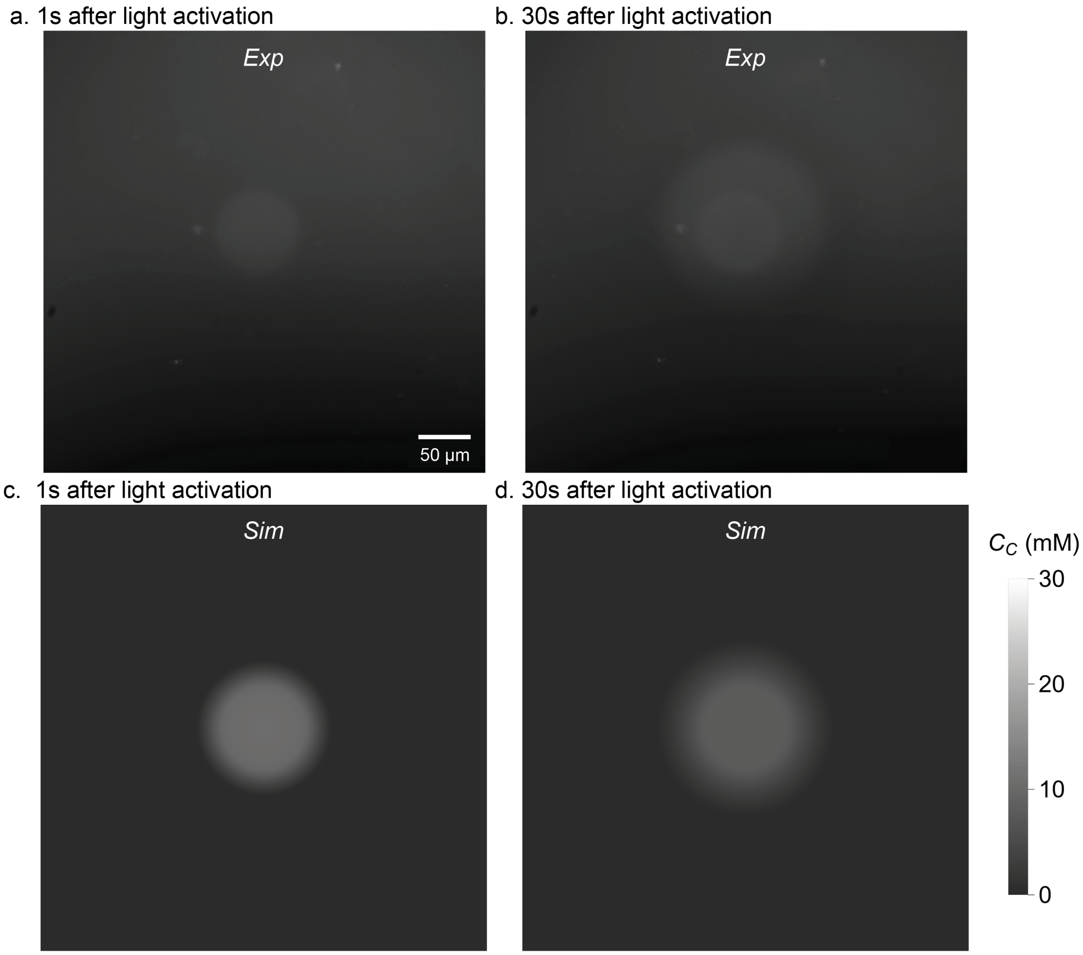

FIG. S20. **Visualization of light-induced Ca^{2+} release with Ca^{2+} indicator dye rhodamine-2.** (a) Image taken immediately after 1 s of light activation, showing a faint fluorescence signal indicating Ca^{2+} release. The illumination diameter is 75 μm. See also SI video Part 3, Section VIII . (b) Image taken after 30 s of light illumination, showing a further spread or diffusion of Ca^{2+}. (c) and (d) Corresponding simulation images of the diffusing Ca^{2+} concentration, colored to match the experimental images.

G. Network growth requires degradation of chelator

In Fig. S22, we show simulation results indicating that for the network to grow in size over the course of both the continuous and pulsed light protocols, it is necessary that the DMNP-EDTA chelators degrade upon photolysis and release of Ca^{2+}. If the chelators are perfectly able to re-uptake Ca^{2+} after photolysis, then the total available pool of Ca^{2+} does not increase in time indefinitely and the system reaches a steady state at finite size. Chelators thus provide a controllable, but not infinitely rechargeable, way to release free Ca^{2+} to the system.

The experimental value of β is not known. However, we note that the precise value of the degradation parameter β does not appear to have a strong effect on the growth rate of the network, provided that it is not equal to 1, as other steps presumably become rate limiting.

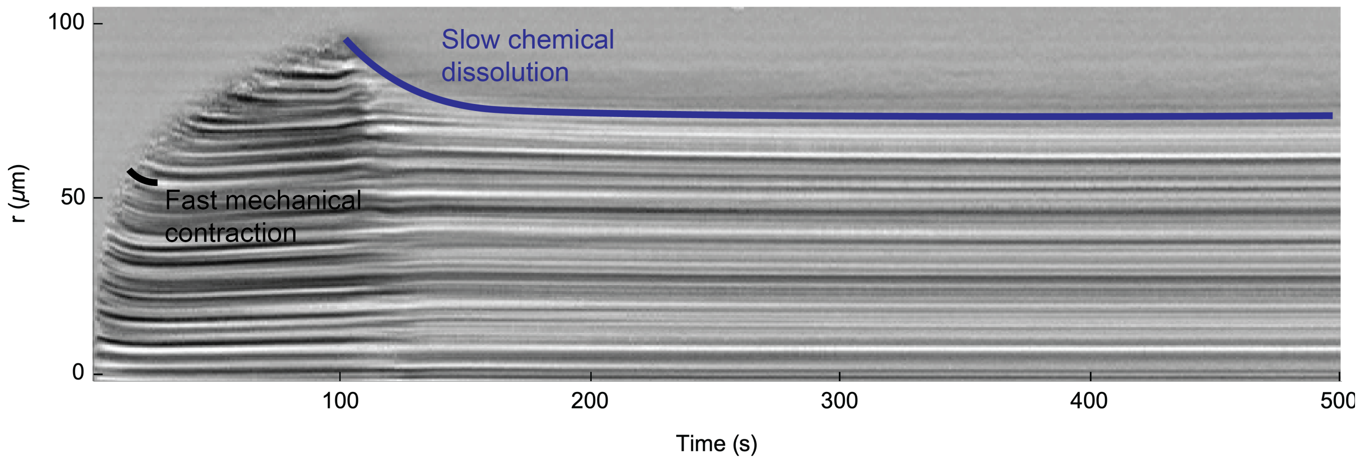

FIG. S21. **Comparison of mechanical contraction and chemical dissociation.** Blow-up of Fig. 2b of the main text, highlighting the kymograph features corresponding to fast mechanical contraction at the active boundary and slow chemical dissolution after the light is turned off.

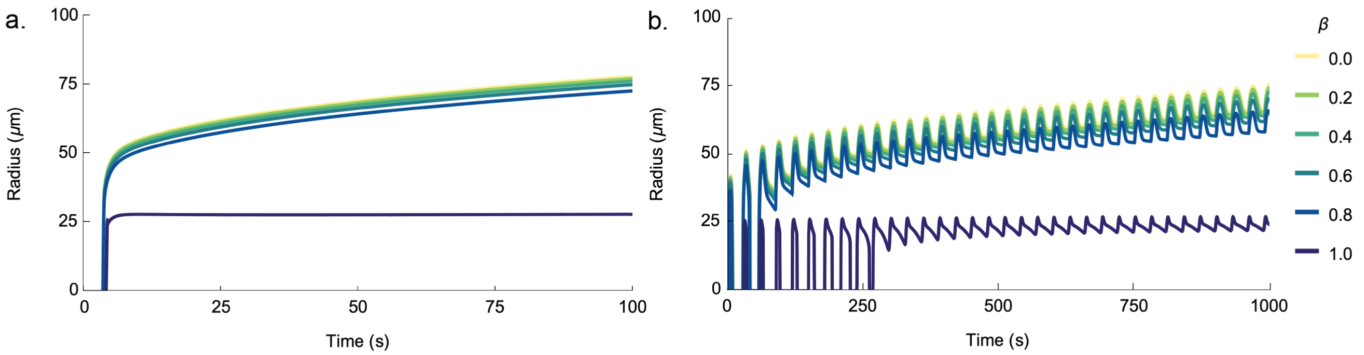

FIG. S22. **Effect of chelator degradation on network growth.** (a) Simulated growth radius as a function of time for various values of the degradation parameter β, for the continuous light protocol. For $\beta = 1$, there is no degradation of chelator upon photolysis and Ca^{2+} release, and for $\beta = 0$ there is complete degradation. (b) Same as panel a, for the pulsed light protocol.

H. Comparing the active region in light-on state under pulsed and continuous protocols

In Fig. 3 of the main text we show how the size of the Ca^{2+}-active region remains (CAR) larger for the pulsed protocol than for the continuous light protocol. In Fig. S23 we quantify this by measuring the radial size of the CAR $|W_a|$, which is the radial extent of the part of the system in which the radial velocity V_r is greater than 0.2 μm/s in magnitude. We also average the radial velocity magnitude $\overline{|V_r|}$ over the CAR. We average over the time that the light is on time for each protocol, capturing 100 s of light activation in the continuous protocol and 30 pulses (30 s of light-on time) in the pulsed protocol. Simulations semi-quantitatively match the experimental data (S23b).

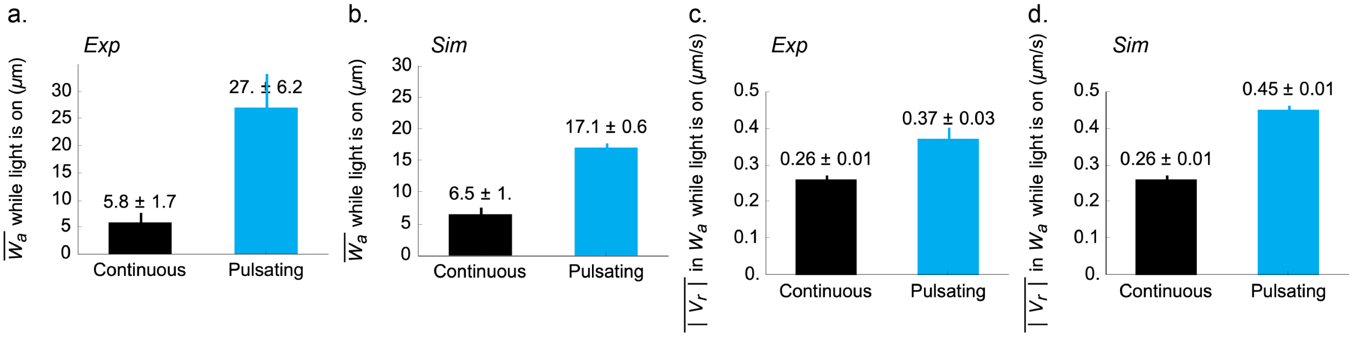

FIG. S23. **Active widths and mean radial velocities under continuous and pulsed conditions.** (a) Measurements of the active width $\overline{W_a}$ (i.e., radial spatial extent for which $|V_r| > 0.2$ μm) for the continuous and pulsed protocols, using an illumination diameter of 75 μm. Error bars represent the standard deviation aggregated over time and space of the averaging window. The data for the continuous protocol was sampled every second and averaged over the 100 s duration the light was on. The data for the pulsed protocol was sampled every 30 seconds during each 1-second light-on interval, at times 1 s, 31 s, 61 s, ..., up to 601 s. (b) Same as panel a, for the simulation. (c) Same as panel a, for the average magnitude of the radial velocity in the active region. (d) Same as panel c, for the simulation.

I. Varying the frequency of light pulses

Here we explore the effect of varying the frequency of light pulses in the pulsed protocol. In experiments, we carry out a protocol in which the pulse length and cycle length (cf. Fig. S6 above) are scaled by the same factor in a fixed ratio of 1/30, shown in Fig. S24a, which we also do in simulations, shown in Fig. S24b. Despite a quantitative difference in the extent of growth between simulation and experiments (discussed above), we observe qualitative agreement on two key features. First, the size of the initial network growth increases with the pulse length. Additionally, following the end of a pulse, the network radius decreases in an apparent biphasic manner, with a quick drop in size followed by a slower decay. The slopes of these two phases of network shrinking do not seem to depend strongly on the pulse length, reflecting instead the chemical kinetics of Tcb2 inactivation and unbinding from the network.

We also test in simulation a protocol in which we fix the pulse length at 1 s and increase the cycle length, shown in Fig. S24c. This makes apparent that the growth of the network using the pulse protocol decreases with increasing cycle length. The network size decays in a roughly linear manner following the cessation of light, and the network will grow faster if this period of decay has a shorter duration. The increase in network size upon each cycle of light is roughly, but not exactly, the same across cycle number and cycle length conditions. Given these results, our choice of default pulse length and cycle length as 1 s and 30 s respectively is a practical choice that makes the experimental run time more manageable without qualitatively changing the behavior we expect with longer cycle lengths.

J. Varying the ratio of DMNP-EDTA to Tcb2

Here we discuss the effects of varying the ratio of DMNP-EDTA to Tcb2 in the experimental solution. We expect that increasing the concentration of free DMNP, which are available to take up the Ca^{2+} ions released by Ca^{2+}-bound DMNP-EDTA upon photolysis, reduces the available supply of Ca^{2+} to bound to Tcb2 and thereby hinders

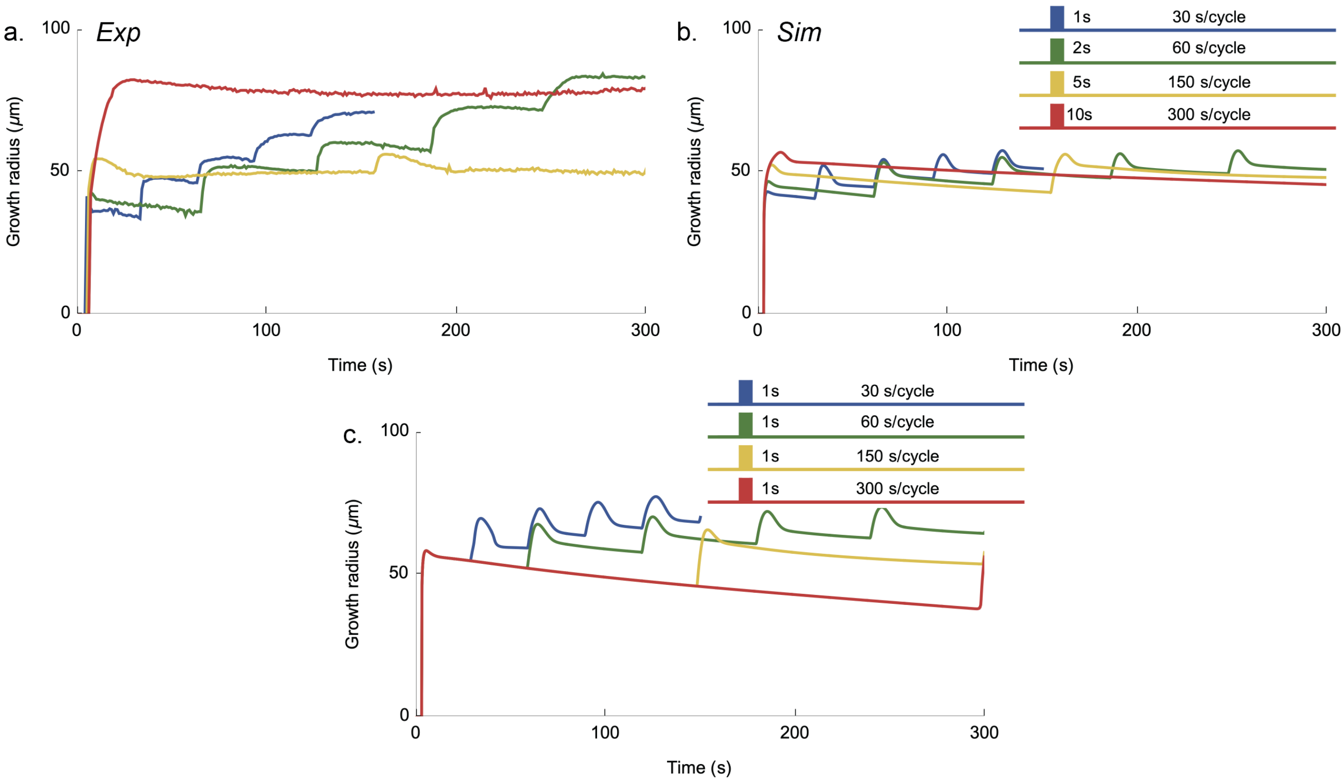

FIG. S24. **Network growth dynamics with varying light pulse frequency.** (a) Experimental growth radius as a function of time for various pulse protocols. See panel b for the color legend. (b) Simulated growth radius as a function of time for various pulse protocols. The color legend indicates the pulse length and cycle length for each protocol. (c) Simulated growth radius as a function of time for various pulse protocols in which the pulse length is fixed at 1 s.

growth of the network. Indeed, we find from repeated trials that networks fail to nucleate and grow even with the large illumination diameter (100 μm) when we double the concentration of free DMNP-EDTA in solution. One such example is shown in Fig. S25, which we compare to the default concentrations used elsewhere. Simulations qualitatively support this result, showing a reduced growth rate with greater DMNP-EDTA concentrations than at normal conditions. However, in simulations the nucleation is not prevented from occurring at high DMNP-EDTA concentrations, indicating that further refinement of the nucleation model is needed in future work. These results suggest that a somewhat precise balance of chemical concentrations is needed to prevent the system from being overwhelmed with chelators that soak up the available Ca^{2+}.

K. Boundary accumulation depends on diffusion of Tcb2

Here we study the accumulation of bound Tcb2 near the periphery of the network. We used 1.01 μM Fluoresbrite Polychromatic Red Microspheres (PolyScience 18660-5) for boundary accumulation experiment. In Fig. S26 we show experimental images of embedded fluorescent particles which report on the local density of the system. After light activation, we observe that the formed network has radially structured density profile, with signatures of accumulation and depletion near the network boundary.

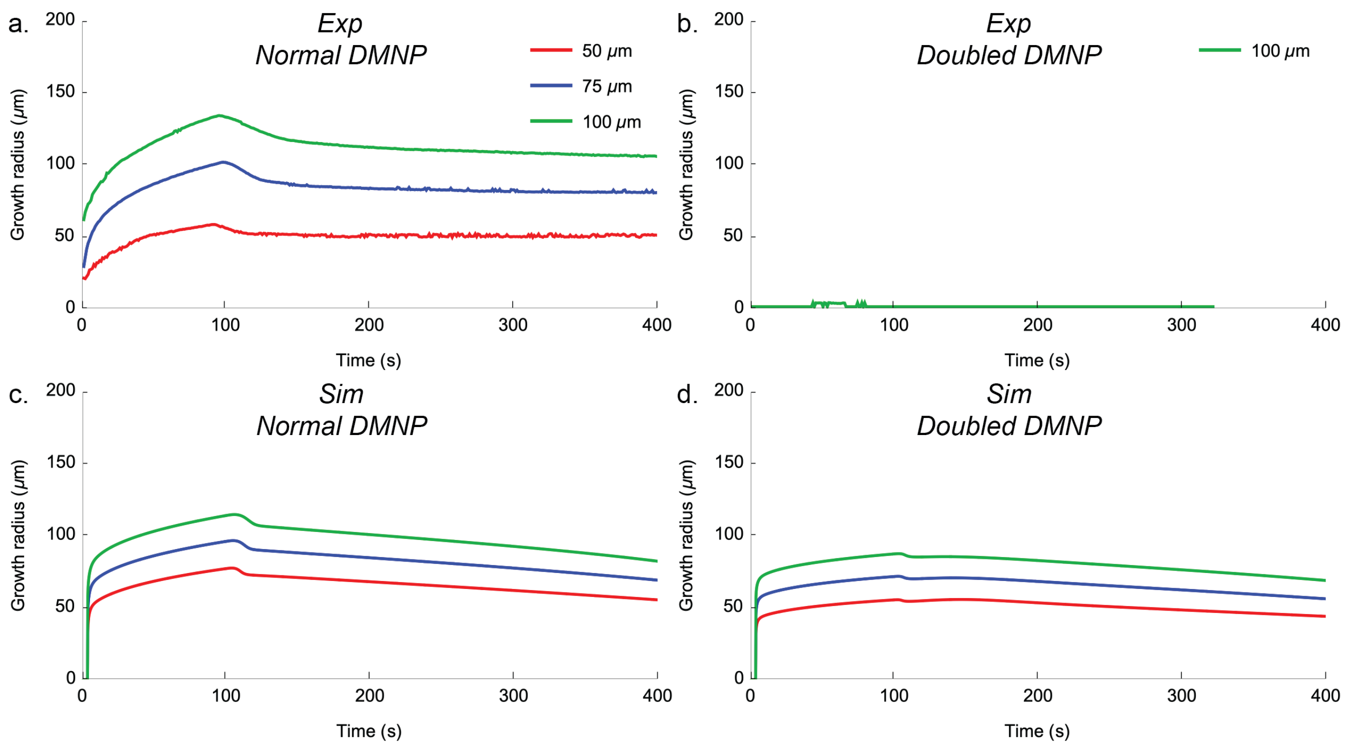

FIG. S25. **Network growth dynamics with varying concentrations of Tcb2 and DMNP.** (a) and (b) Experimental growth radius as a function of time for various illumination diameters, at a normal (used elsewhere in the paper) and doubled DMNP-EDTA concentrations. (c) and (d) Same as panels a and b, for simulations.

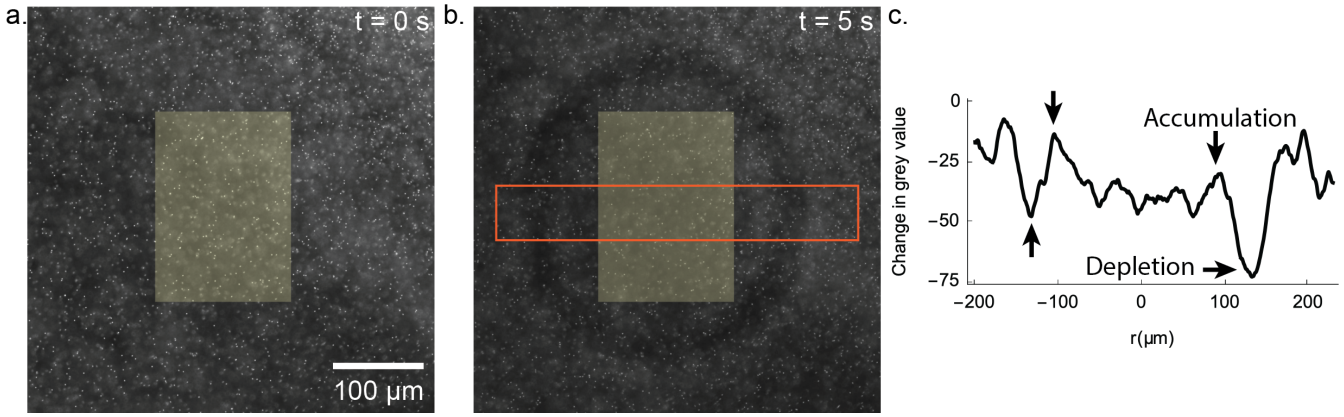

FIG. S26. **Boundary effects.** (a) Fluorescent image of a Tcb2 network before light activation with embedded 1 μm beads. (b) Same as panel a, after 5 seconds of light activation in the labeled yellow region. (c) Average change after 5 s in grey values within the horizontal orange strip in the middle of panel b, with a 70 μm height and 400 μm width.

In Fig. S27 we demonstrate in simulation that the radial density structure of bound Tcb2 near the periphery of the network is largely controlled by the diffusion of Tcb2. In the network interior, there is little diffusing Tcb2, setting up a concentration gradient compared with the exterior. Tcb2 then diffuses down the gradient and binds to the network. As it encounters the peripheral region of the network, it starts to bind first there before it diffuses to further inside. This leads to a bump of density of bound Tcb2 at the periphery. Additionally, there is a trough in density just exterior to the network because of local depletion via diffusion towards the network. As we turn down the diffusion constant

of Tcb2, we see that the profile becomes more homogeneous as expected.

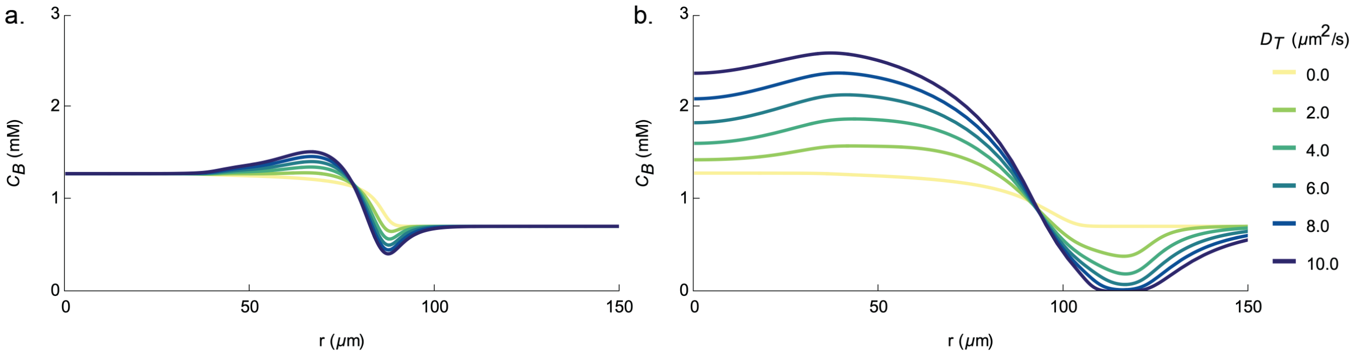

FIG. S27. **Effect of Tcb2 diffusion on boundary accumulation.** (a) Simulated profile of the bound Tcb2 concentration after 30 s of the continuous light protocol for varying values of the Tcb2 diffusion constant D_T. (b) Same as panel a, for the 30th cycle of the pulsed light protocol.

L. Spatially dependent particle transport

In Fig. 5a and b of the main text, we demonstrate that lipid particles are displaced in the presence of a growing Tcb2 network, exhibiting complex dynamics: some particles are pulled inward, others are pushed outward, and some experience sequential push-then-pull motion. This behavior primarily depends on each particle's initial radial distance from the illumination zone. Particles close to the zone are predominantly pulled inward, while those at greater distances are predominantly pushed outward. We illustrate this radial dependence in Fig. S28a, where we plot the radial motion of each particle including its initial offset. We successfully transported three types of particles: polystyrene beads (diameter: 10–20 μm), liposomes, and lipid particles, with their displacement profiles shown in Fig. S28. The pulling effect arises from mechanical contraction of the Tcb2 network, while the pushing effect likely results from steric interactions between particles and the expanding network periphery. Particles closer to the illumination zone become trapped within the forming network and experience primarily pulling forces, whereas those at greater distances encounter the slowly expanding network front and experience primarily pushing forces. Note that current simulations do not incorporate steric interactions between the Tcb2 network and particles, thus modeling only the pulling effect via network contraction.

M. Formation of a peripheral network

Although the Tcb2 network grows at high density primarily within the illumination region, the rapid diffusion of Ca^{2+} outward from the illumination region also sets up a lower-density peripheral network. This rapidly forming network can then sterically interact with embedded particles, such as the liposome in Fig. 5c of the main text, on the time scale of seconds. We highlight the growth of this peripheral network in Fig. S29.

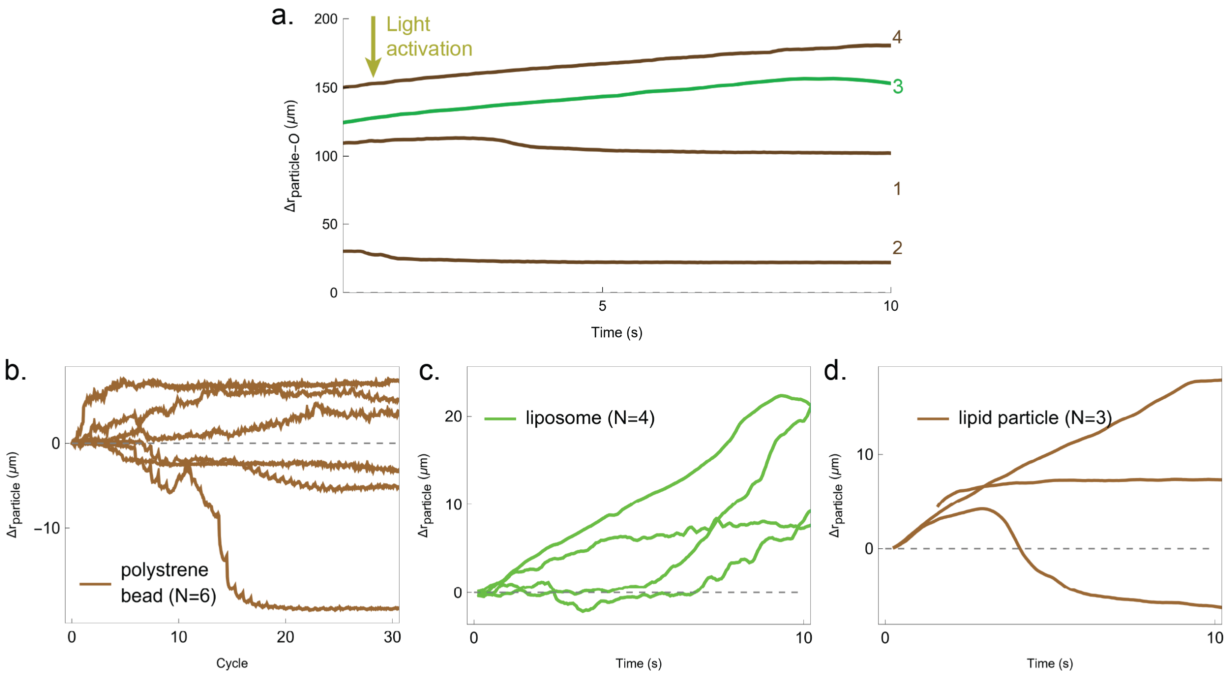

FIG. S28. **Dependence of pushing and pulling on initial radial distance.** (a) This plot corresponds to Fig. 5c of the main text, replotted so that the initial radial distance of each object from the origin is shown. Displacements of (b) polystyrene beads (N=6) under pulsed light (1s on, 29s off), (c) liposomes (N=4), and (d) lipid particles (N=3) under continuous light.

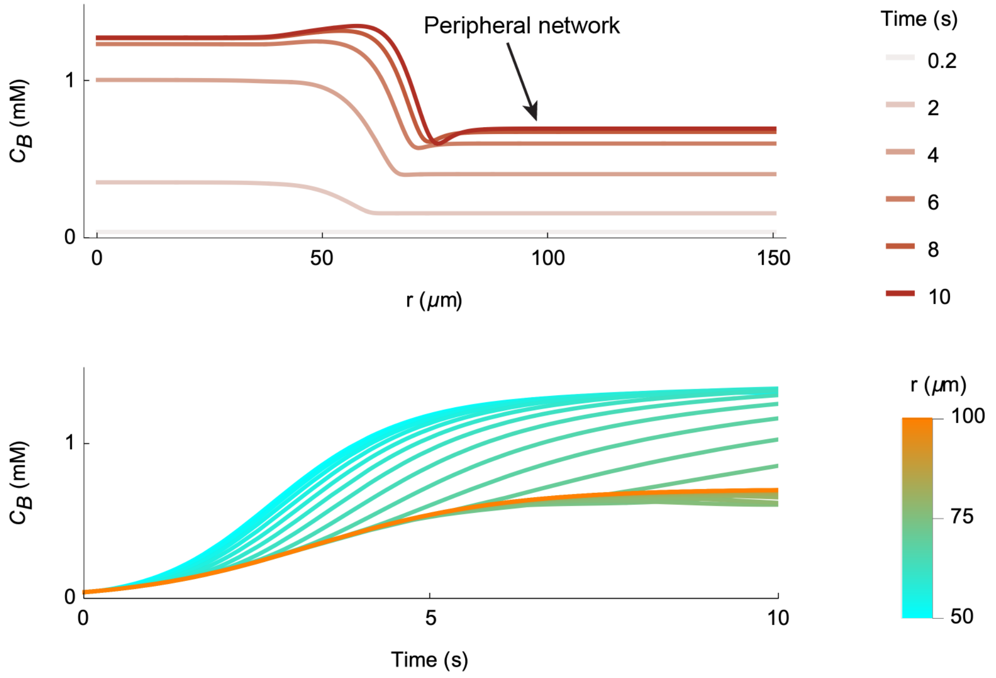

FIG. S29. **Growth of the peripheral network.** *Top*: The radial profile of bound Tcb2 concentration at different times during the early part of a continuous light protocol with illumination diameter of 75 μm. The peripheral network, at lower density and further from central illuminated zone, is indicated. *Bottom*: An alternate view showing time profiles at different radial slices, highlighting that the main and peripheral networks grow on similar timescales, though to different densities.

[1] Josh Abramson, Jonas Adler, Jack Dunger, Richard Evans, Tim Green, Alexander Pritzel, Olaf Ronneberger, Lindsay Willmore, Andrew J Ballard, Joshua Bambrick, Sebastian W Bodenstein, David A Evans, Chia-Chun Hung, Michael O'Neill, David Reiman, Kathryn Tunyasuvunakool, Zachary Wu, Akvilė Žemgulytė, Eirini Arvaniti, Charles Beattie, Ottavia Bertolli, Alex Bridgland, Alexey Cherepanov, Miles Congreve, Alexander I Cowen-Rivers, Andrew Cowie, Michael Figurnov, Fabian B Fuchs, Hannah Gladman, Rishub Jain, Yousuf A Khan, Caroline M R Low, Kuba Perlin, Anna Potapenko, Pascal Savy, Sukhdeep Singh, Adrian Stecula, Ashok Thillaisundaram, Catherine Tong, Sergei Yakneen, Ellen D Zhong, Michal Zielinski, Augustin Žídek, Victor Bapst, Pushmeet Kohli, Max Jaderberg, Demis Hassabis, and John M Jumper. Accurate structure prediction of biomolecular interactions with AlphaFold 3. *Nature*, 630(8016):493–500, June 2024.

[2] K Ohnishi and Y Watanabe. Purification and some properties of a new Ca2+-binding protein (TCBP-10) present in tetrahymena cilium. *J. Biol. Chem.*, 258(22):13978–13985, November 1983.

[3] T Takemasa, K Ohnishi, T Kobayashi, T Takagi, K Konishi, and Y Watanabe. Cloning and sequencing of the gene for tetrahymena calcium-binding 25-kda protein (TCBP-25)*. *Journal of Biological Chemistry*, 264(32):19293–19301, November 1989.

[4] K Hanyu, T Takemasa, O Numata, M Takahashi, and Y Watanabe. Immunofluorescence localization of a 25-kda tetrahymena EF-hand ca(2+)-binding protein, TCBP-25, in the cell cortex and possible involvement in conjugation. *Exp. Cell Res.*, 219(2):487–493, August 1995.

[5] Osamu Numata, Kazuko Hanyu, Tetsuya Takeda, and Yoshio Watanabe. Chapter 24 tetrahymena Calcium-Binding proteins, TCBP-25 and TCBP-23. In David J Asai and James D Forney, editors, *Methods in Cell Biology*, volume 62, pages 455–465. Academic Press, January 1999.

[6] Jerry E Honts. Purification of tetrahymena cytoskeletal proteins. *Methods Cell Biol.*, 109:379–391, 2012.

[7] Adina M Kilpatrick, Jerry E Honts, Heidi M Sleister, and C Andrew Fowler. Solution NMR structures of the C-domain of tetrahymena cytoskeletal protein Tcb2 reveal distinct calcium-induced structural rearrangements. *Proteins*, 84(11):1748–1756, November 2016.

[8] T Takemasa, T Takagi, T Kobayashi, K Konishi, and Y Watanabe. The third calmodulin family protein in tetrahymena. cloning of the cdna for tetrahymena calcium-binding protein of 23 kda (tcbp-23). *Journal of Biological Chemistry*, 265(5):2514–2517, 1990.

[9] R B Hawkes and D V Holberton. Myonemal contraction of spirostomum. i. kinetics of contraction and relaxation. *J. Cell. Physiol.*, 84(2):225–236, October 1974.

[10] Françoise Ruiz, Nicole Garreau de Loubresse, Catherine Klotz, Janine Beisson, and France Koll. Centrin deficiency in paramecium affects the geometry of basal-body duplication. *Curr. Biol.*, 15(23):2097–2106, December 2005.

[11] L Mahadevan and P Matsudaira. Motility powered by supramolecular springs and ratchets. *Science*, 288(5463):95–100, April 2000.

[12] Gaurav Misra, Richard B Dickinson, and Anthony J C Ladd. Mechanics of vorticella contraction. *Biophys. J.*, 98(12):2923–2932, June 2010.

[13] spasmin [vorticella convallaria] - protein - NCBI. https://www.ncbi.nlm.nih.gov/protein/AAD00995.1. Accessed: 2024-11-7.

[14] RecName: Full=25 kDa calcium-binding protein; AltName: Full=TCBP-25; C - protein - NCBI. https://www.ncbi.nlm.nih.gov/protein/P09226.2?report=fasta. Accessed: 2024-10-21.

[15] RecName: Full=Caltractin ICL1a; AltName: Full=Centrin-1 - protein - NCBI. https://www.ncbi.nlm.nih.gov/protein/Q27177.2. Accessed: 2024-11-7.

[16] calmodulin - tetrahymena thermophila - protein - NCBI. https://www.ncbi.nlm.nih.gov/protein/7441480. Accessed: 2024-11-12.

[17] Arnold J T M Mathijssen, Joshua Culver, M Saad Bhamla, and Manu Prakash. Collective intercellular communication through ultra-fast hydrodynamic trigger waves. *Nature*, 571(7766):560–564, July 2019.

[18] Jeffrey L Salisbury. Centrosomes: Sfi1p and centrin unravel a structural riddle. *Curr. Biol.*, 14(1):R27–9, January 2004.

[19] Paul V Ruijgrok, Rajarshi P Ghosh, Sasha Zemsky, Muneaki Nakamura, Rui Gong, Lin Ning, Robert Chen, Vipul T Vachharajani, Alexander E Chu, Namrata Anand, Raphael R Eguchi, Po-Ssu Huang, Michael Z Lin, Gregory M Alushin, Jan T Liphardt, and Zev Bryant. Optical control of fast and processive engineered myosins in vitro and in living cells. *Nat. Chem. Biol.*, 17(5):540–548, May 2021.

[20] Steven A Redford, Jonathan Colen, Jordan L Shivers, Sasha Zemsky, Mehdi Molaei, Carlos Floyd, Paul V Ruijgrok, Vincenzo Vitelli, Zev Bryant, Aaron R Dinner, and Margaret L Gardel. Motor crosslinking augments elasticity in active nematics. *Soft Matter*, 20(11):2480–2490, 2024.

[21] Ian Linsmeier, Shiladitya Banerjee, Patrick W Oakes, Wonyeong Jung, Taeyoon Kim, and Michael P Murrell. Disordered actomyosin networks are sufficient to produce cooperative and telescopic contractility. *Nature communications*, 7(1):12615, 2016.

[22] Ryota Sakamoto and Michael P Murrell. Mechanical power is maximized during contractile ring-like formation in a biomimetic dividing cell model. *Nature communications*, 15(1):9731, 2024.

[23] Camelia G Muresan, Zachary Gao Sun, Vikrant Yadav, A Pasha Tabatabai, Laura Lanier, June Hyung Kim, Taeyoon Kim, and Michael P Murrell. F-actin architecture determines constraints on myosin thick filament motion. *Nat. Commun.*, 13(1):7008, November 2022.

[24] Tyler D Ross, Heun Jin Lee, Zijie Qu, Rachel A Banks, Rob Phillips, and Matt Thomson. Controlling organization and forces in active matter through optically defined boundaries. *Nature*, 572(7768):224–229, August 2019.

[25] Zijie Qu, Dominik Schildknecht, Shahriar Shadkhoo, Enrique Amaya, Jialong Jiang, Heun Jin Lee, David Larios, Fan Yang, Rob Phillips, and Matt Thomson. Persistent fluid flows defined by active matter boundaries. *Communications Physics*, 4(1):1–9, August 2021.

[26] Ashwini Krishna, Mariya Savinov, Niv Ierushalmi, Alex Mogilner, and Kinneret Keren. Size-dependent transition from steady contraction to waves in actomyosin networks with turnover. July 2022.

[27] Maya Malik-Garbi, Niv Ierushalmi, Silvia Jansen, Enas Abu-Shah, Bruce L Goode, Alex Mogilner, and Kinneret Keren. Scaling behaviour in steady-state contracting actomyosin networks. *Nat. Phys.*, 15(5):509–516, May 2019.

[28] Volker Schaller, Christoph Weber, Christine Semmrich, Erwin Frey, and Andreas R Bausch. Polar patterns of driven filaments. *Nature*, 467(7311):73–77, 2010.

[29] Tim Sanchez, Daniel T N Chen, Stephen J DeCamp, Michael Heymann, and Zvonimir Dogic. Spontaneous motion in hierarchically assembled active matter. *Nature*, 491(7424):431–434, November 2012.

[30] Marina Soares e Silva, Martin Depken, Björn Stuhrmann, Marijn Korsten, Fred C MacKintosh, and Gijsje H Koenderink. Active multistage coarsening of actin networks driven by myosin motors. *Proc. Natl. Acad. Sci. U. S. A.*, 108(23):9408–9413, June 2011.

[31] Ryota Sakamoto, Masatoshi Tanabe, Tetsuya Hiraiwa, Kazuya Suzuki, Shin'ichi Ishiwata, Yusuke T Maeda, and Makito Miyazaki. Tug-of-war between actomyosin-driven antagonistic forces determines the positioning symmetry in cell-sized confinement. *Nat. Commun.*, 11(1):3063, June 2020.

[32] Ryota Sakamoto, Ziane Izri, Yuta Shimamoto, Makito Miyazaki, and Yusuke T Maeda. Geometric trade-off between contractile force and viscous drag determines the actomyosin-based motility of a cell-sized droplet. *Proc. Natl. Acad. Sci. U. S. A.*, 119(30):e2121147119, July 2022.

[33] William M Bement, Marcin Leda, Alison M Moe, Angela M Kita, Matthew E Larson, Adriana E Golding, Courtney Pfeuti, Kuan-Chung Su, Ann L Miller, Andrew B Goryachev, et al. Activator–inhibitor coupling between rho signalling and actin assembly makes the cell cortex an excitable medium. *Nature cell biology*, 17(11):1471–1483, 2015.

[34] Linnea M Lemma, Minu Varghese, Tyler D Ross, Matt Thomson, Aparna Baskaran, and Zvonimir Dogic. Spatio-temporal patterning of extensile active stresses in microtubule-based active fluids. *PNAS nexus*, 2(5):pgad130, 2023.

[35] Actin pelleting assay. `https://puresoluble.com/protocols/actin-pelleting-assay/`, September 2019. Accessed: 2024-10-9.

[36] Michael F Ashby and David Cebon. Materials selection in mechanical design. *Le Journal de Physique IV*, 3(C7):C7–1, 1993.

[37] Carlos Floyd, Suriyanarayanan Vaikuntanathan, and Aaron R Dinner. Simulating structured fluids with tensorial viscoelasticity. *The Journal of Chemical Physics*, 158(5), 2023.

[38] Guido C Faas, Kinga Karacs, Julio L Vergara, and Istvan Mody. Kinetic properties of dm-nitrophen binding to calcium and magnesium. *Biophysical journal*, 88(6):4421–4433, 2005.

[39] Kerry R Delaney and Vahid Shahrezaei. Uncaging calcium in neurons. *Cold Spring Harbor Protocols*, 2013(12):pdb–top079491, 2013.

[40] Richard S Sutton and Andrew G Barto. *Reinforcement Learning: An Introduction.* MIT press, 2018.

[41] Carlos Floyd, Aaron R Dinner, and Suriyanarayanan Vaikuntanathan. Tailoring interactions between active nematic defects with reinforcement learning. *arXiv preprint arXiv:2411.09588*, 2024.

[42] Carlos Floyd, Aaron R Dinner, and Suriyanarayanan Vaikuntanathan. Learning to control non-equilibrium dynamics using local imperfect gradients. *arXiv preprint arXiv:2404.03798*, 2024.

[43] David Silver, Guy Lever, Nicolas Heess, Thomas Degris, Daan Wierstra, and Martin Riedmiller. Deterministic policy gradient algorithms. In *International conference on machine learning*, pages 387–395. Pmlr, 2014.

[44] Timothy P Lillicrap, Jonathan J Hunt, Alexander Pritzel, Nicolas Heess, Tom Erez, Yuval Tassa, David Silver, and Daan Wierstra. Continuous control with deep reinforcement learning. *arXiv preprint arXiv:1509.02971*, 2015.

[45] Jun Tian and other contributors. Reinforcementlearning.jl: A reinforcement learning package for the julia programming language, 2020.

[46] Katsu Nishiyama, John Berezney, Michael M Norton, Akshit Aggarwal, Saptorshi Ghosh, Michael F Hagan, Zvonimir Dogic, and Seth Fraden. Closed-loop control of active nematic flows. *arXiv preprint arXiv:2408.14414*, 2024.

[47] Alexander Kirillov, Eric Mintun, Nikhila Ravi, Hanzi Mao, Chloe Rolland, Laura Gustafson, Tete Xiao, Spencer Whitehead, Alexander C. Berg, Wan-Yen Lo, Piotr Dollár, and Ross Girshick. Segment anything. *arXiv:2304.02643*, 2023.

[48] Kadir Nar. segment-anything-video: MetaSeg: Packaged version of the segment anything repository.
