# Supplementary material for: Light-induced assembly and repeatable actuation in Ca2+-driven chemomechanical protein networks
Source: Nat Commun. 2026 Feb 21;17:3016. doi: 10.1038/s41467-026-69651-2 (PMC13035854; doi:10.1038/s41467-026-69651-2)
Supplement: Supplementary file 2 — Description of Additional Supplementary Files [file 41467_2026_69651_MOESM2_ESM.pdf]

Description of Additional Supplementary Files

Supplementary Movies

Supplementary Movie Part 1

- Section I video: Ca^{2+} diffuses into a sandwich-structured chamber from left to right, inducing a gel-like transition in the Tcb2 solution and contraction is happening along the boundary.
- Section II video: Tcb2 network formation, imaged using DIC, for a star-shaped DMD pattern.
- Section II video: Dynamic Tcb2 network formation by a 0.125 Hz clockwise cyclic light pattern.
- Section III video: Tcb2 network formation under illumination with a 75 µm diameter for 100 seconds. Right: Corresponding experimental analysis PIV image.

Supplementary Movie Part 2

- Section III video: Corresponding Tcb2 simulation showing light intensity, Tcb particle radial velocity v_r, bound inactivated Tcb2 (CBI), bound activated Tcb2 (CBA), diffusing Ca^{2+} (CC), DMNP with Ca^{2+} (CD), DMNP-EDTA without Ca^{2+} (CD*), diffusing inactivated Tcb2 (CDI) and diffusing activated Tcb2 (CDA) under illumination with a 75 µm diameter for 100 seconds.
- Section III video: Concentration profiles of all compounds as a function of radial position with a 75 µm diameter for 100 seconds.
- Section IV Video: Tcb2 network formation under illumination with a 75 µm diameter, using a 1-second light-on and 29-second light-off cycle. Right: Corresponding experimental PIV analysis image.
- Section IV Video: Simulation of Tcb2 dynamics under a 1-second light-on and 29-second light-off cycle.
- Section IV Video: Concentration profiles of all compounds as a function of radial position under a 1-second light-on and 29-second light-off cycle.
- Section V video: Embedded fluorescent 1 µm beads within the Tcb2 network under light activation in the labeled yellow region.
- Section VI Video: 150 cycles repeatability test with 50 µm diameter under a 1-second light-on and 29-second light-off cycle.

Supplementary Movie Part 3

- Section VII Video: Tcb2 network facilitates the movement of liposomes and lipid particles with a designed light pattern.
- Section VII Video: Tcb2 network facilitates the movement of 10–20 µm polystyrene beads with multi-pattern light activation.
- Section VIII Video: Rhodamine-2 Ca^{2+} indicator showing the Ca^{2+} profile during Tcb2 network formation.
