# Supplementary material for: Light-induced assembly and repeatable actuation in Ca2+-driven chemomechanical protein networks
Source: Nat Commun. 2026 Feb 21;17:3016. doi: 10.1038/s41467-026-69651-2 (PMC13035854; doi:10.1038/s41467-026-69651-2)
Supplement: Supplementary file 6 — Transparent Peer Review file [file 41467_2026_69651_MOESM6_ESM.pdf]

nature portfolio

Peer Review File

Light-induced assembly and repeatable actuation in Ca^{2+}-driven chemomechanical protein networks

Corresponding Author: Dr Saad Bhamla

This manuscript has been previously reviewed at another journal. This document only contains information relating to versions considered at Nature Communications.

This file contains all reviewer reports in order by version, followed by all author rebuttals in order by version.

Version 0:

Reviewer comments:

Reviewer #2

(Remarks to the Author)
I have revised the original version of this manuscript for [redacted] as Reviewer 2. I carefully checked Authors' answers to my comments and questions. I feel that they addressed all of my concerns and comments well, and the quality of the manuscript has been improved significantly. I recommend publication in Nature Communications.

(Remarks on code availability)

Open Access This Peer Review File is licensed under a Creative Commons Attribution 4.0 International License, which permits use, sharing, adaptation, distribution and reproduction in any medium or format, as long as you give appropriate credit to the original author(s) and the source, provide a link to the Creative Commons license, and indicate if changes were

made.
In cases where reviewers are anonymous, credit should be given to 'Anonymous Referee' and the source.
The images or other third party material in this Peer Review File are included in the article’s Creative Commons license, unless indicated otherwise in a credit line to the material. If material is not included in the article’s Creative Commons license and your intended use is not permitted by statutory regulation or exceeds the permitted use, you will need to obtain permission directly from the copyright holder.
To view a copy of this license, visit https://creativecommons.org/licenses/by/4.0/
